# Continuous adaptive chemotherapy dosing by using planet-formation-inspired dynamics: analytical and *in silico* insights

**DOI:** 10.3389/fonc.2026.1761791

**Published:** 2026-05-11

**Authors:** Marco P. Soares dos Santos, Sérgio X. F. Santos, Rodrigo M. C. Bernardo, João V. Vidal, Gil Gonçalves

**Affiliations:** 1 Center for Mechanical Technology and Automation (TEMA), Department of Mechanical Engineering, University of Aveiro, Aveiro, Portugal; 2 Intelligent Systems Associate Laboratory (LASI), Guimarães, Portugal; 3 Department of Physics and Aveiro Institute of Materials (CICECO), University of Aveiro, Aveiro, Portugal; 4 Department of Physics and Institute for Nanostructures, Nanomodelling and Nanofabrication (I3N), University of Aveiro, Aveiro, Portugal

**Keywords:** cancer treatment, chemotherapy control, continuous chemotherapy dosing, dynamic control, intermittent chemotherapy dosing, planet formation

## Abstract

**Background:**

Current therapeutic planning of chemotherapy is based on empirical dosimetry and intermittent dose administration, which can cause poorly defined therapeutic timepoints due to tumor heterogeneities and related highly uncertain tumor dynamics. Dynamic control strategies have been investigated to overcome these limitations, but their dependence on reliable mathematical models predicting tumor dynamics and/or their inherent intuition have hampered their use in clinical practice. This study proposes a control method for administering chemotherapeutic drugs using planet-formation-like dynamics to ensure high adaptability and versatility to uncertain tumor dynamics, taking advantage of the evolutive, deterministic, adaptive, convergent, and robust behavior of planetary systems.

**Methods:**

The planet-formation-inspired controller was designed using accretion and gravitational attraction that occur during planet formation, such that chemotherapeutic trajectories are attracted in an analogous pattern as planetesimal bodies (masses/particles) are attracted to protoplanets. The necessary conditions to guarantee stability are provided, as well as extensive simulation results for cyclophosphamide, different tumor volumes (200 and 2,000 mm^3^), and different modeling characterizations (therapy with and without chemoresistance). The Sobol indices were also computed to analyze the influence of controller parameters on planet-formation-inspired administration as well as to find the influence of astrophysical-like dynamics on mechanobiological tumor dynamics.

**Results:**

This controller ensured either tumor remission or retention states, regardless of the initial tumor volume, tumor growth dynamics, and the potential impact of chemoresistance. Its non-linear drug administration resulted in highly robust, stable, and adaptive behaviors, with detumorization represented as an effective response metric that does not explicitly incorporate inter-individual biological variability and is associated with a uniformization of treatment times. Such planet-formation-inspired administration forces detumorization dynamics defined by geometro-pharmacokinetics that significantly outperform both non-adaptive and adaptive intermittent administrations. We also found that drag forces inside protoplanets are usually the most dominant astrophysical phenomenon influencing mechanobiological dynamics in resistance-free chemotherapy, while accretion is usually the most dominant one in cancer therapies with chemoresistance.

**Conclusion:**

This study provides a computationally promising proof-of-concept highlighting that planet-formation-inspired dynamics can effectively overcome the limitations of tumor heterogeneity and uncertain tumor dynamics, presenting significant improvements compared to conventional chemotherapy administration.

## 1 Introduction

Even though remarkable advances have been achieved in cancer treatment, the global mortality-to-incidence ratio is ≈48.5% (1, 2). The number of new cancer cases per year is expected to exceed 28 million by 2040, a burden corresponding to 47% growth over 20 years (2, 3). Chemotherapy remains the mainstay treatment for most cancer types, currently applied in clinical practice in approximately 58% of cancer treatments, a demand that is estimated to remain unchanged until 2040 (country-dependent range between 51% and 62%) (4, 5). This therapeutic modality has been administered to approximately 10 million patients per year but is estimated to reach 15 million patients by 2040, which will require an estimated increase in the global workforce of 65k–99k oncologists (4).

Although numerous chemotherapeutic drugs and agents are currently used in clinical practice, tested in clinical trials, and explored in cutting-edge research, including combinations of chemotherapeutic agents and therapies (5, 6), the chemotherapy secondary effects, mainly related to treatment-related toxicity and adverse events, cause considerable morbidity and mortality (7). Indeed the 30-day mortality after chemotherapy usually ranges between 2% and 50% (8, 9). Additionally, more than 25% of the patients still experience life-threatening toxicities that induce cardiac, renal, and neurological dysfunction, among many other side effects (7, 10, 11). The dosage remains a critical factor for chemotherapeutic effectiveness (12). On the one hand, high doses enhance apoptosis but can induce adverse chemotoxicity effects, both in the short and long term; on the other hand, low doses enhance chemoresistance and may not reduce early mortality (13, 14). Therefore, developing effective drug delivery strategies is crucial to minimizing the effects of both high and low doses.

Chemotherapeutic drugs are dose dependent, but intermittent dose administration regimens remain the most common clinical therapeutic approach, even though clear evidence highlights that continuous dosing has the potential to lengthen survival rates and mitigate long-term adverse events (15–17). Poor therapeutic indexes are obtained in many tumor scenarios as current dosimetry is empirically defined and intermittently administered. Complex tumor dynamics, intratumor variability, and unpredictability strongly affect disease progression and therapeutic response, which can hardly be managed by intermittent administration, even following dosage escalation and de-escalation schemes (recommendations can be scarce) (18, 19). In addition, the mechanisms underlying the synergetic effects of pharmacodynamics and tumor dynamics remain unclear (20). Recently, McGehee and Mori (16) showed that continuous administration provides superior performance responding to chemotherapy resistance compared to intermittent therapy (both adaptive and fixed dose) in terms of robustness to uncertain tumor dynamics and cumulative toxicity. Several adaptive drug delivery methods have been proposed to address this major challenge. Non-adaptive intermittent therapy responds less robustly to uncertainty in initial conditions than adaptive intermittent therapy (using bang-bang control) (16). The most sophisticated dynamic dosing strategies developed so far to explore the adaptability potential use optimal control and fuzzy logic control (21–23). Although many studies have been conducted in the last two decades within this scope (24, 25), they remain within the academic realm of theoretical control. Indeed they have not achieved the confidence level required for their translation into clinical practice because of (i) their strong dependence on the accuracy of mathematical models predicting tumor dynamics, (ii) their reduced personalized controllability in intermittent dosing, even with established frequent administration schedules, and/or (iii) their inherent intuition. These are “artificial” control approaches, as they neglect the rationality and effectiveness expressed in nature. Artificial neural network control has emerged as a highly promising alternative to model-based approaches to improve adaptability to tumor heterogeneity (26, 27). However, their limitations to perform (intermittent or continuous) adaptive administration include a certain degree of non-determinism and a limited adaptability compared to other natural systems, which can result in unsatisfactory performances due to uncertainties and non-linearities of many realistic scenarios. Therefore, one of the major challenges in cancer treatment is to find a dosing strategy with the ability to respond with stable and robust performance under high tumor heterogeneities and related highly uncertain tumor dynamics.

**Figure 1:**
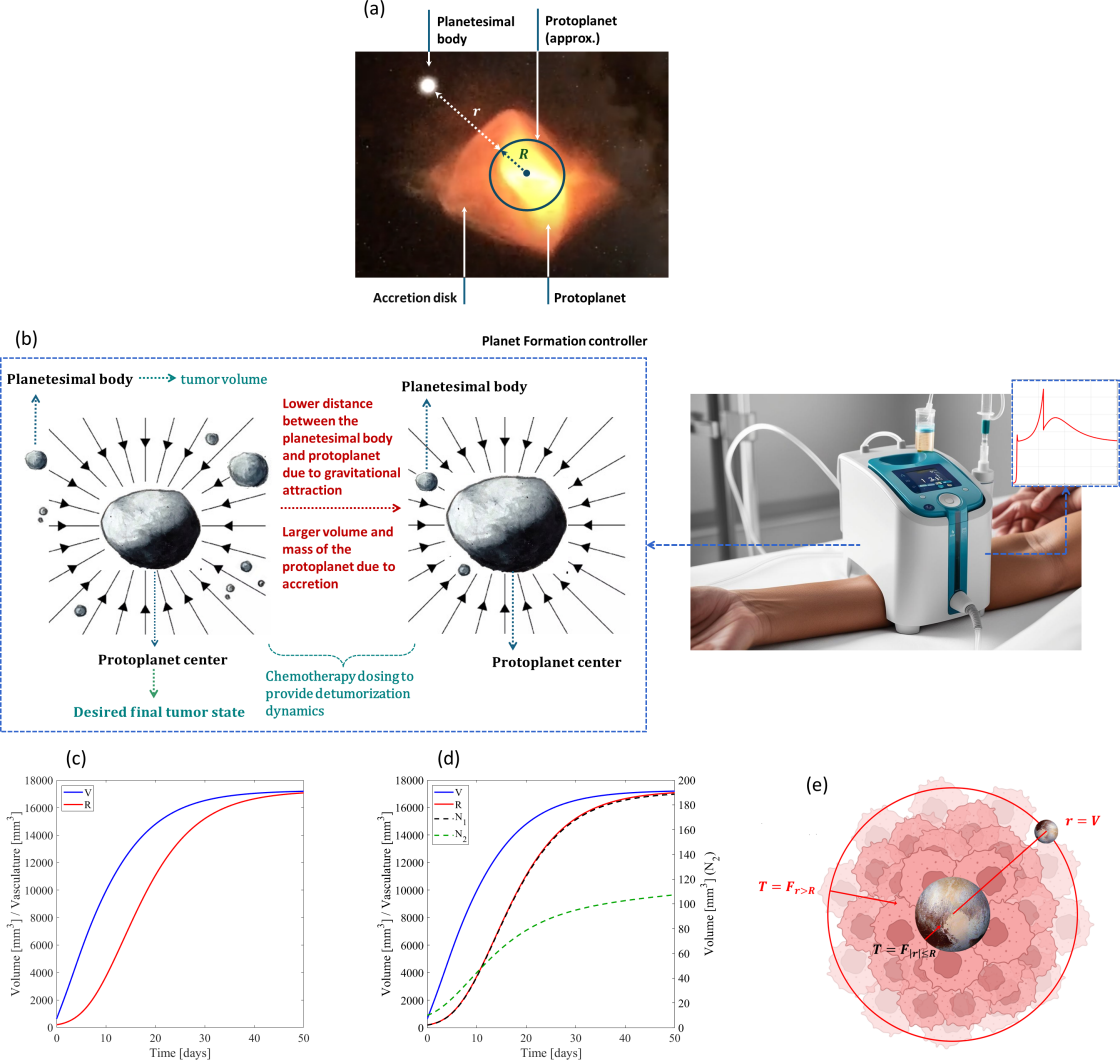
**(A)** Planet forming scenario considering a planetary system composed of a protoplanet (as a uniform sphere), a planetesimal body, and an accretion disk (image created using a picture from AstroPix). **(B)** Chemotherapy using PF-like dynamics, including the proposed analogy between a PF system and a tumor system. Tumor growth dynamics without PF control **(C)** for resistance-free chemotherapy and **(D)** considering chemoresistance. **(E)** Proposed analogy between a PF system and a tumor system, including the regions where planetesimal bodies, related to protoplanets, may be located (image created using pictures from AstroPix and BioRender, Gonçalves, G (2026). https://BioRender.com/v4ne6p2).

In this study, we developed a new chemotherapeutic dosing strategy based on planet formation (PF) dynamics. Planetary systems can exhibit evolutive, deterministic, adaptive, and convergent behaviors, which are critical characteristics to design highly adaptable and robust controllers (28). Besides that, astrophysics-inspired controllers can ensure energy minimization compared to non-inspired dynamic control methods when controlling highly non-linear dynamic systems (29), which suggests that such nature-inspired approaches hold potential to significantly reduce the cytotoxicity of chemotherapy. Therefore, we hypothesized that the use of physical laws expressing PF dynamics could ensure high adaptability and versatility to highly uncertain tumor dynamics. Even though PF dynamics are significantly different from dynamics to be controlled in real-world problems, the rational of planetary-inspired control allows their use in drug delivery systems for cancer chemotherapy. Indeed PF is ruled by gravitational attraction as well as by migration of masses (particles in orbit around protoplanets), accretion (accumulation of particles, increasing the mass and volume of planetary objects) and erosion of both protoplanets and planetesimal bodies, and planetesimal–protoplanet and protoplanet–protoplanet collisions (30). On the one hand, the gravitational pull of a planetary formation system can be used to “trap” desired detumorization trajectories; on the other hand, accretion mechanism mimicking realistic pebble accretion can provide sophisticated error integration, which can be used to significantly improve the controller responses to errors in desired detumorization trajectories. Tumor geometrodynamics were then established using accretion and gravitational attraction that occur during PF, such that chemotherapeutic trajectories are attracted in an analogous pattern as planetesimal bodies (masses/particles) are attracted to protoplanets (**Figures 1A, B**). Tumors were analogically considered as masses inside a planetary formation system, in which the protoplanet center represents the cancer remission, and the chemotherapy drugs represent the gravitational attraction pulling the tumor mass toward the center (where a complete remission is reached). We show, through *in silico* tests, that computational decision-making using control algorithms expressing PF-like dynamics results in highly effective treatments for different tumor volumes and mechanobiological characterizations as well as for highly uncertain tumor dynamics. Such findings required the following tasks:

1. Find suitable tumor growth models with the ability to accurately express general tumor dynamics, including chemoresistance.
2. Design the PF controller, establishing a bridge between planetary formation dynamics and tumor dynamics.
3. Investigate if the PF controller can effectively force stable detumorization until remission or if retention states are achieved. Detumorization stability demands to demonstrate the uniqueness of detumorization dynamics toward equilibrium tumor states (remission or retention) as well as to show that these equilibrium tumor states are stable.
4. Robustness analysis must then be conducted to show the superiority performance of this controller responding to significant tumor heterogeneity.
5. Compare the detumorization dynamics defined by the FP controller with the ones provided by both non-adaptive and adaptive intermittent administrations.
6. Investigate the influence of PF controller parameters on drug administration as well as the influence of astrophysical dynamics (integrated into the PF controller) on mechanobiological tumor dynamics.

Our ultimate goal is to propose a new anticancer treatment methodology that overcomes the current survival probabilistic paradigm. Classic approaches only offer patients survival probabilities (e.g., 5-year survival of 6% in glioblastoma (31); differently, astrophysical-inspired control offers treatment effectiveness, even though the treatment duration is not known prior to treatments. Our promising findings, supported by our new conceptual advance, go beyond those treatment concepts supporting current administration methods, ultimately leading to significantly improved patient outcomes.

## 2 Methods

### 2.1 Tumor growth models

Tumor dynamics have been modeled by two main modeling approaches: deterministic modeling and stochastic modeling (32). This study uses two deterministic models to obtain tumor growth dynamics (33, 34), as they are widely used due to their ability to predict highly non-linear tumor dynamics. These analytical models were validated using experimental data from Lewis lung tumors in mice (35), but they have been effectively used to clinically predict the growth of multiple tumor types (34). These are not cancer-specific, such that the use of geometrodynamics forced by PF controllers (see Section 2.2) can be established as a highly promising methodology for administering chemotherapy drugs. A Gompertzian model was firstly used to analyze the performance of the PF controller, disregarding chemotherapy resistance. It expresses the dynamic behavior of the primary tumor volume $V[{\text{mm}}^{3}]$, carrying capacity of the vasculature $R[{\text{mm}}^{3}]$, and drug concentration C[mg/kg] as a function of the antiangiogenic dynamics and pharmacodynamics resulting from a drug administration T[mg/kg],

(1) $$\begin{array}{c} \dot{V}=-\xi \ln(\frac{V}{R})V-\varphi VC\\ \dot{R}=bV-(\mu +dV^{\frac{2}{3}})R-\eta RC\\ \dot{C}=-\lambda C+T. \end{array}$$

Concerning the tumor volume dynamics, the term $\xi \ln(\frac{V}{R})V$ represents the tumor growth dynamics (limited by vasculature), and φVC expresses the cytotoxic effect of chemotherapy on the tumor volume; concerning the vasculature dynamics, the term bV corresponds to the stimulation of angiogenesis by the tumor, $(\mu +dV^{\frac{2}{3}})R$ expresses the inhibition of vasculature by the tumor, and ηRC is the anti-angiogenic effect due to chemotherapy (33, 34). **Table 1** shows the model parameters and related primarily parametrization (prior robustness analyses); **Figure 1C** presents the tumor growth dynamics for such parametrization, considering the initial tumor volume $V_{0}=200\;{\text{mm}}^{3}$ and initial vasculature capacity $R_{0}=625\;{\text{mm}}^{3}$. The Gompertzian biphasic model developed by Bodzioch, Bajger, and Foryś (35, 36) was also used to analyze the primary tumor geometrodynamics imposed by the PF controller for clinical scenarios expressing chemotherapy resistance. It includes the dynamic behavior of sensitive tumor tissue $N_{1}$ $\left[{\text{mm}}^{3}\right]$ and resistant tumor tissue $N_{2}$ $\left[{\text{mm}}^{3}\right]$, as formulated in (Equation 2). and parametrized in **Table 1**. Its parametrization considers experimental data from animal models and theoretical predictions, but it is also able to clinically predict tumor growth (34, 36–38). **Figure 1D** highlights the dynamic behavior of $N_{1}$, $N_{2}$, and R for $V_{0}=200\;{\text{mm}}^{3}$ and $R_{0}=625\;{\text{mm}}^{3}$.

**Table 1:** Model parameters of tumor growth models with/without chemotherapy resistance, primary parametrization, and range of variation for performance analyses.

Parameter	Description	Value ^a^	Range of variation^b^
$\varphi \;\left[{\text{conc}}^{-1}{\text{ day}}^{-1}\right]$	Cytotoxic effect on the tumor volume V	4	4≤φ≤12
$\eta \;\left[{\text{conc}}^{-1}{\text{ day}}^{-1}\right]$	Cytotoxic effect on the vasculature R	1	0.3≤η ≤3
$\xi \;\left[{\text{day}}^{-1}\right]$	Tumor growth related to V	0.192	0.0576≤ξ≤0.576
$b\;\left[{\text{day}}^{-1}\right]$	Angiogenic stimulation	5.85	1.755≤b≤17.55
$d\;\left[{\text{mm}}^{-2}{\text{day}}^{-1}\right]$	Angiogenic inhibition	0.00873	0.0026≤d≤0.0262
$\mu \;\left[{\text{day}}^{-1}\right]$	Loss of endothelial support	0.02	0.006≤μ≤0.06
$\lambda \;\left[{\text{day}}^{-1}\right]$	Drug elimination within the body	4.16 ^b^	---
$\xi_{1}\left[{\text{day}}^{-1}\right]$	Tumor growth related to sensitive $N_{1}$	0.192	$0.0576\leq \xi_{1}\leq 0.576$
$\xi_{2}\left[{\text{day}}^{-1}\right]$	Tumor growth related to sensitive $N_{2}$	0.096	$0.0288\leq \xi_{2}\leq 0.288$
$\tau_{1}\;\left[{\text{day}}^{-1}\right]$	Cellular mutation rate (from sensitive cells to resistant cells)	$2\times 10^{-5}$	$6\times 10^{-6}\leq \tau_{1}\;\leq 6\times 10^{-5}$
$\tau_{2}\;\left[{\text{day}}^{-1}\right]$	Cellular reverse mutation rate (from resistant cells to sensitive cells)	$1\times 10^{-5}$	$3\times 10^{-6}\leq \tau_{2}\;\leq 3\times 10^{-5}$
$V_{0}$ $\left[{\text{mm}}^{3}\right]$	Initial tumor volume V	200 | 2000	---
$R_{0}\;\left[{\text{mm}}^{3}\right]$	Initial vasculature capacity R	625 | 6250	---
$N_{10}$ $\left[{\text{mm}}^{3}\right]$	Initial sensitive tumor tissue	$0.95\;V_{0}$	---
$N_{20}$ $\left[{\text{mm}}^{3}\right]$	Initial resistant tumor tissue	$0.05\;V_{0}$	---
$T_{max}\;\left[\text{mg}/\text{kg}\right]$	Maximum dosing	3 | 75 ^c^	---

^a^ Refs (15, 36–44).

^b^ Range of variation defined as 0.3 parameter≤parameter ≤3 parameter, excluding 1φ≤φ≤3φ.

^c^ Drug-specific parameter: λ=4.16 for cyclophosphamide (38).

^d^ Maximum dosing without/with chemoresistance.

(2) $$\begin{array}{c} {\dot{N}}_{1}=-(\tau_{1}+\xi_{1}\ln(\frac{N_{1}+N_{2}}{R}))N_{1}+\tau_{2}N_{2}-\varphi N_{1}C\\ {\dot{N}}_{2}=-(\tau_{2}+\xi_{2}\ln(\frac{N_{1}+N_{2}}{R}))N_{2}+\tau_{1}N_{1}\\ \dot{R}=b(N_{1}+N_{2})-(\mu +d{\left(N_{1}+N_{2}\right)}^{\frac{2}{3}})R-\eta RC\\ \dot{C}=-\lambda C+T. \end{array}$$

Concerning the sensitive tumor dynamics, the term $(\tau_{1}+\xi_{1}\ln(\frac{N_{1}+N_{2}}{R}))N_{1}$ corresponds to the growth of the sensitive tumor tissue, $\tau_{2}N_{2}$ to the mutation from resistant cells to sensitive cells, and $\varphi N_{1}C$ to the cytotoxic effect of chemotherapy on the sensitive tumor tissue; concerning the resistant tumor dynamics, the term $(\tau_{2}+\xi_{2}\ln(\frac{N_{1}+N_{2}}{R}))N_{2}$ corresponds to the growth of the resistant tumor tissue and $\tau_{1}N_{1}$ to the mutation from sensitive cells to resistant cells; finally, the term $b(N_{1}+N_{2})$ from the vasculature dynamics corresponds to the stimulation of angiogenesis by both sensitive and resistant tumor tissues, $(\mu +d{\left(N_{1}+N_{2}\right)}^{\frac{2}{3}})R$ to the inhibition of vasculature by both sensitive and resistant tumor tissues, and ηRC to anti-angiogenic effect due to chemotherapy (36, 37).

### 2.2 Planet formation controller

Our new drug administration methodology can be achieved by defining a tumor geometrodynamics according to an analogy-based bridge between control principles and PF phenomena (**Figures 1A, B, E**), in which required tumor volume trajectory $\left(u\in {\mathbb{R}}^{+}\right)$ ↦ protoplanet center; chemotherapy dosing $\;(T\in {\mathbb{R}}^{+}\;)\mapsto$ attraction exhibited by the protoplanet; current tumor volume $\left(V\in {\mathbb{R}}^{+}\right)$ ↦ position of the planetesimal body attracted to a protoplanet; difference between V and u, i.e., the therapeutic error (r=V−u≥0, r∈ℝ) ↦ distance between the planetesimal body and the protoplanet center ( r=V if a complete remission ( u=0) is required). The PF control law defines a chemotherapy dosing $T_{PL}\;$given by

(3) $$T_{PL}=\{\begin{array}{c} \frac{G(M_{0}+e^{k_{1}{\left(\int_{0}^{t}r\;d\tau \right)}^{2}})}{{\left(r+\delta \right)}^{2}}\;,\;\;\;\;\;\;\;\;\;\;\;\;\;\;\;\;\;\;\;\;\;\;\;\;\;\;\;\;\;\;\;\;\;\;\;r>R,\\ \frac{G(M_{0}+e^{k_{1}{\left(\int_{0}^{t}r\;d\tau \right)}^{2}})}{R^{4}}({\left(r+\delta \right)}^{2}(1-k_{0})+k_{0}R(r+\delta ))\;,\;\;\;\;\;|r|\leq R, \end{array}$$

where G represents the gravitational constant ( $G\in {\mathbb{R}}^{+}$), R is the protoplanet radius ( $R\in {\mathbb{R}}^{+}$), $M_{0}$ denotes the initial protoplanet mass ( $M_{0}\in {\mathbb{R}}^{+}$), and M is the protoplanet mass ( $M\in {\mathbb{R}}^{+}$) given by

(4) $$\text{M}=M_{0}+e^{k_{1}{\left(\int_{0}^{t}r\;d\tau \right)}^{2}},$$

which models the accretion dynamics of protoplanets as an exponential increase (45), where $k_{1}\in {\mathbb{R}}^{+}$ is a constant that can be used to define different accretion dynamics. This accretion phenomenon was integrated into the control law to eradicate small-scale tumoral states, as it provides sophisticated integral dynamics. An accretion rate parameter $k_{1}=k_{m}t_{m}$ ( $k_{1}\in {\mathbb{R}}^{+}$) was introduced to establish the “time” component as the key parameter to ensure effective treatments under tumor resistance to chemotherapy ( $t_{m}=t$). $k_{1}$ was defined independent of the time for tumor dynamics without chemotherapy resistance ( $t_{m}=1$). Equation 3 was obtained as follows. The attraction force $F_{r>R}\;$between a desired tumor volume throughout time t (the protoplanet center by analogy) and the current tumor volume (the position of the planetesimal body by analogy), when the planetesimal body is outside the protoplanet ( r>R), is then obtained by using Newton’s law of universal gravitation (Equation 5) (46),

(5) $$F_{r>R}=\frac{GM}{r^{2}}.$$

This attraction was integrated into the control law to significantly reduce large tumor volumes. The protoplanet was considered a uniform sphere (**Figure 1A**) (47) with a linear distribution of mass (48, 49). To avoid numerical issues ( R may be too small, requiring $T_{PL}\to \infty$ as r→0) and mathematical indeterminations (which do not allow for an effective analysis of the potential of the PF controller to provide effective cancer treatments), a sufficiently small δ ≳0 ( δ∈ℝ) was introduced. The term $\left({\left(r+\delta \right)}^{2}(1-k_{0})+k_{0}R(r+\delta )\right)$ refers to a drag force $F_{D}\;$inside the protoplanet (Equation 6) (50),

(6) $$F_{D}=-6\pi R\zeta \text{Φ}v,$$

which was integrated to control the approximation dynamics to remission states. Such drag force is considered as a viscous force at low Reynolds numbers, where ζ is the kinematic viscosity ( $\zeta \in {\mathbb{R}}^{+}$), v is the relative planetesimal body velocity ( $v\in {\mathbb{R}}^{+}$), and Φ is the protoplanet density ( $\text{Φ}\in {\mathbb{R}}^{+}$), which can be obtained by the density–mass relationship establishing how the protoplanet attracts planetesimal bodies on regions inside the protoplanet (Equations 7, 8) (48, 50),

(7) $$\text{Φ}=(\frac{{\text{Φ}}_{|r|=R}-{\text{Φ}}_{r=0}}{R})r+{\text{Φ}}_{r=0},$$

(8) $$F_{|r|\leq R}=-\frac{4}{3}G\pi \text{Φ}r,$$

where ${\text{Φ}}_{|r|=R}$ is the minimum Φ, ${\text{Φ}}_{r=0}$ is the maximum Φ, and ${\text{Φ}}_{r=0}=k_{0}$ ${\text{Φ}}_{|r|=R}$, ( $k_{0}\in {\mathbb{R}}^{+}$), i.e., the maximum protoplanet density was considered proportional to the minimum protoplanet density (48, 51). Thus, the following control law can be formulated as

(9) $$T_{PL}=\{\begin{array}{c} \frac{GM}{{\left(r+\delta \right)}^{2}}\;,\;\;\;\;\;\;\;\;\;\;\;\;\;\;\;\;\;\;\;\;\;\;\;\;\;r>R\\ \frac{4}{3}G\pi \left(r+\delta \right)\Phi \;,\;\;\;\;\;\;\;\;\;\;\left|r\right|\leq R, \end{array}$$

to establish a region-dependent control law as a function of R and *r*, in which (i) large therapeutic errors are associated with regions outside the protoplanet, triggering a controller response based on gravitational attraction found in protoplanet-planetesimal system and (ii) regions inside the protoplanet are associated with small therapeutic errors, causing a controller response computed according to attraction and drag forces. The continuity of must be ensured when r=R, where ${\text{Φ}}_{r=0}=0$. So,


$$\frac{GM}{R^{2}}=\frac{4}{3}G\pi R\text{Φ}\Leftrightarrow \text{Φ}=\frac{3M}{4\pi R^{3}}.$$

**Figure 2:**
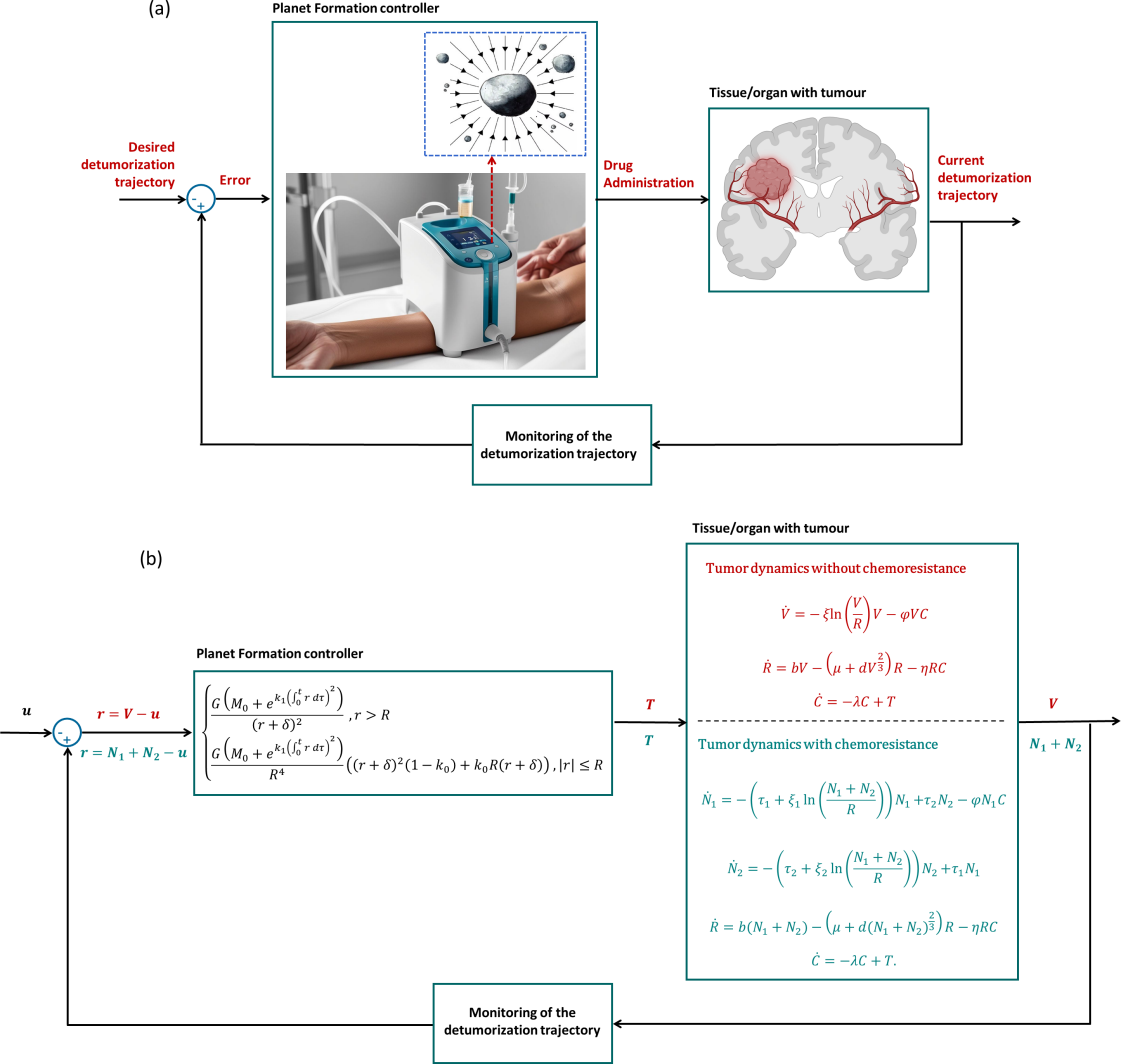
**(A)** Representation of the closed-loop framework for adjusting drug administration (image created using picture from BioRender. Gonçalves, G (2026); https://BioRender.com/l0i7xeo). **(B)** Closed-loop control system designed to test the PF-induced geometrodynamics in cancer treatments. It includes the control law, tumor dynamics, both with and without chemoresistance, and the main control variables (desired detumorization u, control error r, drug administration T, and treatment outcomes V and ( $N_{1}+N_{2}$)).

Inserting $\text{Φ}={\text{Φ}}_{|r|=R}$, expressed by (Equation 7), with ${\text{Φ}}_{r=0}=0$, in (Equation 9), for |r|≤R, we obtain


$$\frac{4}{3}G\pi (r+\delta )\text{Φ}=[(\frac{\frac{3M}{4\pi R^{3}}-k_{0}\frac{3M}{4\pi R^{3}}}{R})(r+\delta )+k_{0}\frac{3M}{4\pi R^{3}}](\frac{4}{3}G\pi (r+\delta ))=\frac{GM}{R^{4}}({\left(r+\delta \right)}^{2}(1-k_{0})+k_{0}R(r+\delta )),$$

as expressed in (Equation 3). **Figure 2** illustrates the closed-loop control system designed to test the PF-induced geometrodynamics in cancer treatments.

### 2.3 Other drug administration approaches

Two additional drug administration approaches used in clinical practice were considered for comparative purposes. Non-adaptive fixed-dose intermittent administration $\;T_{INT}$ was as described in Equation 10,

(10) $$T_{INT}(t)=\{\begin{array}{c} TD,\;\;\;\;\;\;\;0\leq t\leq t_{i}\\ 0\;,\;\;\;\;\;\;t_{i}\leq t\leq T_{INT}, \end{array}$$

where TD is a tolerated dose, $t_{i}$ is the infusion time, and $T_{INT}+t_{i}$ is the intermittency period. Adaptive intermittent administration $\;T_{BB}$ was defined as a bang-bang controller with deadband (16). Concerning tumor dynamics without chemoresistance (Equation 11),

(11) $$T_{BB}(t)=\{\begin{array}{c} TD,\;\;\;\;\;\;\;\;\;\;\;\;\;\;\;\;\;\;V>L_{Sup}\\ 0\;,\;\;\;\;\;\;\;\;\;\;\;\;\;\;\;\;\;\;\;V<L_{Inf}\\ T_{BB}(t-1),\;L_{Inf}\leq V\leq L_{Sup},\; \end{array}$$

where $T_{BB}(t-1)$ is the previous control value, $L_{Sup}$ and $L_{Inf}$ are upper and lower limits, respectively, and $\left(V_{Sup}-V_{Inf}\right)$ is the deadband threshold. When chemoresistance is considered, $T_{BB}$ was defined as described in Equation 12:

(12) $$T_{BB}(t)=\{\begin{array}{c} TD,\;\;\;\;\;\;\;\;\;\;\;\;\;\;\;\;\;\;(N_{1}+N_{2})>L_{Sup}\\ 0\;,\;\;\;\;\;\;\;\;\;\;\;\;\;\;\;\;\;\;\;(N_{1}+N_{2})<L_{Inf}\\ T_{BB}(t-1),\;L_{Inf}\leq (N_{1}+N_{2})\leq L_{Sup\;.}\; \end{array}$$

### 2.4 *In silico* tests

The PF dosing controller was tested under multiple tumor-treatment scenarios (**Table 1**), namely:

- Chemotherapy control with and without chemotherapy resistance.
- Different initial volumes and initial vasculature capacities of primary tumors (33, 44): smaller tumors with $V_{0}=200\;{\text{mm}}^{3}$ and $R_{0}=625\;{\text{mm}}^{3}$; larger tumors with $V_{0}=2,000\;{\text{mm}}^{3}$ and $R_{0}=6250\;{\text{mm}}^{3}$. These tumor volumes ( $V_{0},R_{0})$ are commonly used to analyze the performance of dynamic controllers for the administration of chemotherapy drugs (33, 36, 44). Besides that, they are neither too small nor too large, which usually improves chemotherapy outcomes (52–54). Even though very large tumors are also used to test dynamic controllers (22, 33, 44), large tumors are often surgically removed/debulked to rapidly reduce tumor states and avoid the secondary effects due to aggressive chemotherapy administration (55–57).
- Different pharmacokinetics expressed by cyclophosphamide: This chemotherapy drug was selected as a representative cytotoxic chemotherapeutic agent due to its well-characterized pharmacokinetics, widespread clinical use across multiple cancer types, and extensive documentation of dose–response relationships, including effects related to resistance (5). Systemic drug delivery is assumed, consistent with standard clinical routes for cyclophosphamide (e.g., including intravenous, intramuscular, and intraperitoneal). As such chemotherapy drug is used to treat multiple cancer types and can be administered according to different methods, it strongly supports our new drug administration methodology. Curative dosing of cyclophosphamide has been found between 25 and 150 mg/kg (38–40), although low dose treatments have not exceeded 3 mg/kg (42–44). In this study, maximum dosage administration T=3 mg kg^-1^ and T=75 mg kg^-1^ were considered, respectively, for resistance-free chemotherapies and treatments with chemoresistance. The parameter related to drug elimination within the body ( λ) was found by calculating $\lambda =\ln2/t_{1/2}$, where $t_{1/2}$ is the half-life elimination (15, 38).
- Uncertain tumor dynamics: The robustness of the PF dosing controller was tested using $5\times 10^{3}$ random combinations of model parameters (Equations 1, 2) in a wide range of variation (0.3 parameter ≤ parameter ≤3 parameter, excluding 1 *φ* ≤ *φ* ≤3 *φ*) (40), ensuring the same controller parameters (**Table 2**). As we are assuming that the models defined in Equations 1, 2 consistently represent tumor dynamics, even though the exact values of their parameters may be unknown and inherently random, both parametric and stochastic uncertainty are considered here. As models’ parameters combine multiple underlying biological effects, their variability was used to analyze parametric and stochastic uncertainties as global uncertainties (not specific sources of biological uncertainties) that inherently include intratumor heterogeneity and or inter-patient variability.
- Only when primary tumor volumes $V<10\;{\text{mm}}^{3}$ were tumor states considered as remission states (58) (maximum 200 days of treatment).

**Table 2:** Parameters of both the PF dosing controller (Equation 3) and detumorization trajectories (Equation 13) for different treatment scenarios.

Parameter	Resistance-free chemotherapy		Therapy with chemoresistance	
	CP—SM	CP—LT	CP—SM	CP—LT
R	77	240	17	397
G	20	160	1.75	100
$M_{0}$	89	900	$9\times 10^{3}$	$2.4\times 10^{3}$
$k_{0}$	19	0.1	3.5	50
$k_{m}$	$1.1\times 10^{-4}$	$4\times 10^{-6}$	$1\times 10^{-9}$	$5\times 10^{-8}$
γ	3.6	3.6	9	3
χ	1.8	1.8	0.5	3

CP, cyclophosphamide; SM, smaller tumors ( $V_{0}=200\;{\text{mm}}^{3};\;R_{0}=625\;{\text{mm}}^{3}$); LT, larger tumors ( $V_{0}=2,000\;{\text{mm}}^{3}$ and $R_{0}=6,250\;{\text{mm}}^{3}$).

Both non-adaptive and adaptive intermittent drug administrations were tested for uncertain tumor dynamics. The non-adaptive one was tested for the following cases found in clinical practice:

-
TD=3, 25, 40, and 70 mg kg^-1^
-
$t_{i}=2$ h
-
$T_{INT}+t_{i}=3,\;7,\;14$, and 21 days

Concerning adaptive intermittent administration, the following cases were considered (16):

-
TD=3, 25, 40, and 70 mg kg^-1^
- BB1: $L_{Sup}=V_{0}$ and $L_{Inf}=\frac{V_{0}\;}{2}$; BB2: $L_{Sup}=\frac{3V_{0}}{4}$ and $L_{Inf}=\frac{V_{0}\;}{4}$

The initial sensitive tissues $N_{10}$ was defined as 95% of $V_{0}$ and the initial resistant tissues $N_{20}$ as 5% of $V_{0}$, as defined by previous studies (36, 37). Tumor remission states were considered when detumorization reached $V=5\;{\text{mm}}^{3}$ (criterion for stopping the simulation). The following logistic-sigmoid function was used to define detumorization trajectories:

(13) $$u=\frac{e^{-\gamma (t-\chi )}}{1+e^{-\gamma (t-\chi )}}\;,$$

where parameters γ and χ are described in **Table 2**. This function was chosen due to its continuity and smoothness, expressing a concavity at the beginning of treatments that allows to reduce both the initial and average dosage.

*In silico* tests were performed using Matlab R2024a (v. 24.1, Mathworks) and Simulink (v. 24.1, Mathworks). A fixed-step solver ode14x (fixed-step size: 1 ms) was used to perform the numerical integration. Sensitivity analyses were carried out using the Sobol indices to identify the contributions from each controller parameter to the average drug administration as well as the impact of each astrophysical dynamics on various relevant mechanobiological parameters of tumor growth models (Equations 1, 2). According to Equations 4–8 and taking into account that the gravitational constant G is not a differentiating parameter among different astrophysical dynamics (its influence is found in all considered geometro-pharmacokinetics, as highlighted in Section 3.3.2), the following controller parameters were considered as the most distinctive for each phenomenon found in planet formation: $k_{1}$ for accretion, $M_{0}$ for gravitation, and the set $\left(R,k_{0}\right)$ for drag forces inside protoplanets. Concerning resistance-free chemotherapy, we analyzed the influence of each of these phenomena on tumor growth dynamics, cytotoxic effect of chemotherapy on tumor volume, inhibition of vasculature by the tumor, and anti-angiogenic effect due to chemotherapy. Besides those, similar analyses were conducted for cancer therapy with chemoresistance, namely, the influence of each planetary formation phenomenon on the growth of the sensitive and resistant tumor tissues, cytotoxic effect of chemotherapy on sensitive and resistant tumor tissues, and mutation between resistant cells and sensitive cells. Both first-order Sobol index and total-order Sobol index were computed according to the Saltelli method (59, 60). The uncertainty of each parameter was established in the range between -50% and 50% of the primary parametrization defined in **Table 2**. The Sobol sampling was 1,000.

## 3 Results

### 3.1 Results for resistance-free chemotherapy

#### 3.1.1 Stability of the PF controller

Effective anticancer treatment demands stable detumorization dynamics. A remission state is reached when V=0 for resistance-free chemotherapy; therefore, remission states are unique and equilibrium solutions to the dosing control problem. Thus, we investigated whether the PF controller is able to provide unique treatments, which are the equilibrium tumor states (for both regions where r>R and |*r*|≤*R*), and which conditions are required by the controller to ensure the stabilization of such equilibrium states. A linear approximation of tumor dynamics related to resistance-free chemotherapy (nonlinear system expressed by Equation 1) was carried out to simplify the stability analysis of detumorization trajectories, including to study the existence and uniqueness of solutions as well as to find the equilibrium tumor states. The following assumptions were considered: (i) the detumorization dynamics is continuously differentiable, (ii) remission states are achievable, (iii) disturbances in tumor dynamics are sufficiently small, (iv) model parameters of tumor dynamics are time-invariant (or their variations are sufficiently slow), but tumor dynamics themselves are time-dependent; to highlight this distinction, as well as to highlight that remission states can remain as such for t→∞, we associated each linearized tumor detumorization state to each time instant t, (v) linear stability of all detumorization states implies nonlinear local stability of these states, and (vi) desired detumorization dynamics are smooth (such as the one expressed in Equation 9). To our knowledge, the impact of these assumptions is not significant for the validity of stability conditions. Indeed immune responses (a biological effect that changes model parameters) are not activated as true intermittent behaviors but as periods of immune containment (61), and chemotherapy administered in pulses does not result in pulse behaviors in tumor dynamics (62). Many other biological phenomena may present switching-like dynamics (e.g., phenotypic switching in cancer cells (63), but there is no evidence that such effect results in non-differentiable or large disturbances in cancer dynamics. We found that the PF controller can deliver chemotherapeutic drug delivery dynamics for effective treatment. Appendix A provides an analytical proof highlighting that this dosing controller can ensure stable detumorization trajectories toward remission states ( V→0 as r→0) and remain in such equilibrium states.

#### 3.1.2 *In silico* results

**Figure 3:**
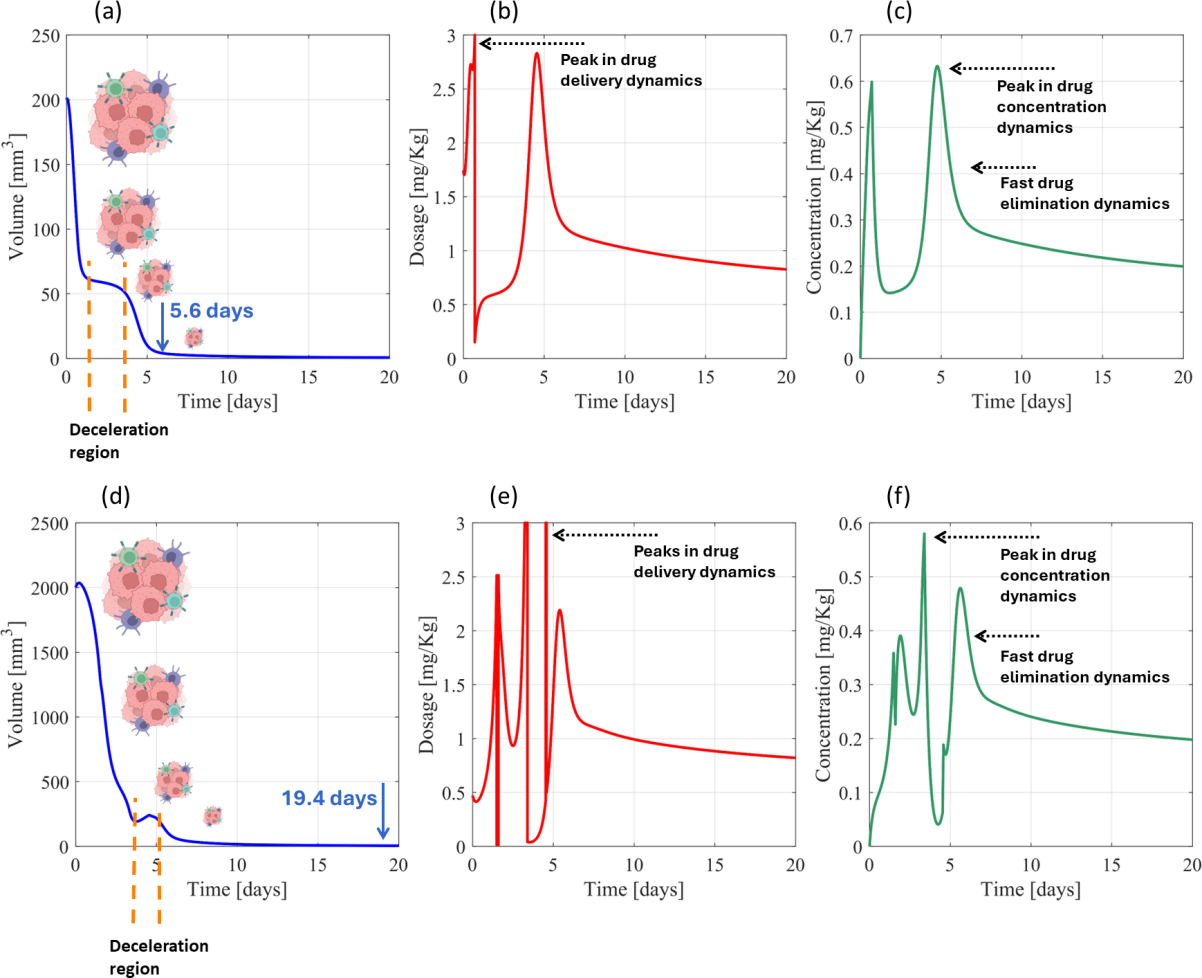
Resistance-free chemotherapy using cyclophosphamide for PF-inspired administration. **(A)** Volume dynamics, **(B)** drug administration dynamics, and **(C)** drug concentration dynamics for the smaller tumor scenario (primary parametrization). **(D)** Volume dynamics, **(E)** drug administration dynamics, and **(F)** drug concentration dynamics for the larger tumor scenario (primary parametrization). **(A, D)** were created using a picture from BioRender, Gonçalves, G (2026); https://BioRender.com/u0ys9z9.

*PF-inspired administration considering the initial conditions* $\left(V_{0},R_{0})=(200,\;625\right)$ ${\text{mm}}^{3}$: Using the tumor primary parameters for the smaller tumor scenario (**Table 1**), the PF controller required 5.6 days to reach a remission state (**Figure 3A**), using 3 and 0.63 mg kg^-1^ of maximum drug administration and concentration, respectively, and 1.08 and 0.26 mg kg^-1^ of average drug administration and average concentration (**Figures 3B, C**). A highly nonlinear dosing dynamics (**Figure 3B**) was found by the controller to force a strong decrease in tumor volumetric dynamics in the first 1.5 days, until the tumor volume ≈60.5 mm^3^ was reached, and around day 3 to ensure accelerated detumorization toward a remission state (**Figure 3A**). Concerning the larger tumor scenario (**Table 1**), highly nonlinear drug dynamics were also necessary to impose a reduction in cancer volumes to <5 mm^3^ (**Figures 3D, E**), including a deceleration in tumor reduction between days 3.7 and 5 (**Figure 3D**). Delivering a maximum and average dosing of 3 and 0.99 mg kg^-1^, respectively (**Figure 3E**), a remission state was predicted after 19.4 days (**Figure 3D**), which was achieved by a maximum and average drug concentration of 0.58 and 0.24 mg kg^-1^, respectively (**Figure 3F**). The difference in the treatment time for small and large tumors (**Figures 3A, D**) was 13.8 days.

**Figure 4:**
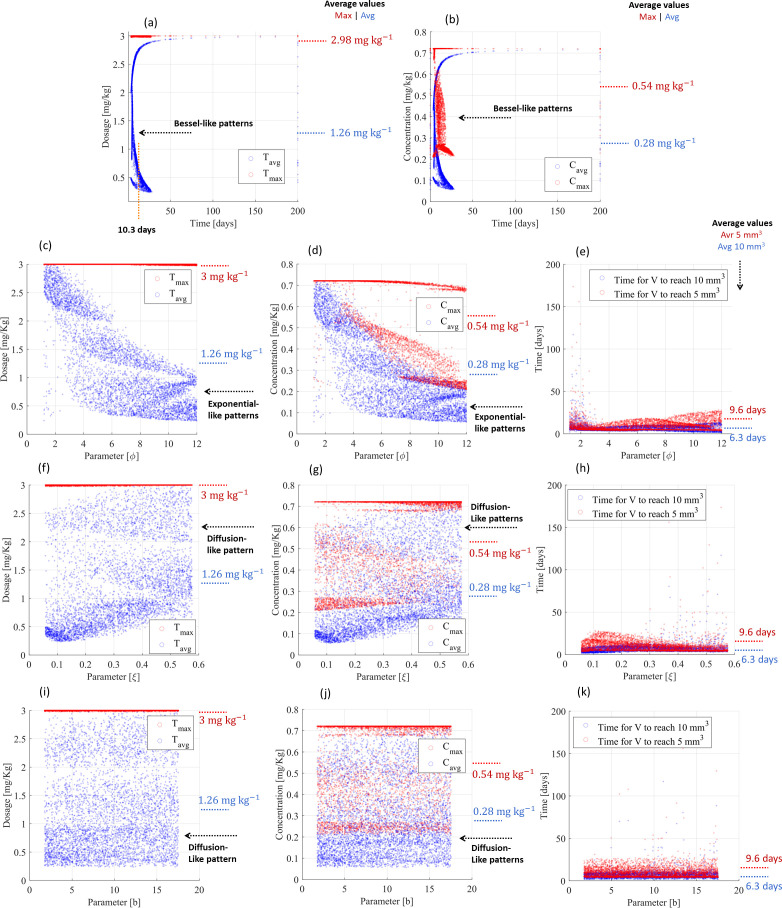
Resistance-free chemotherapy using cyclophosphamide for smaller tumor scenarios and PF-inspired administration. **(A)** Dosing administration and **(B)** drug concentration considering the influence of all biological parameters. Influence of the cytotoxic effect on the tumor volume ( φ) on **(C)** dosing administration, **(D)** drug concentration, and **(E)** treatment time. Influence of the volumetric tumor growth ( ξ) on **(F)** dosing administration, **(G)** drug concentration, and **(H)** treatment time. Influence of the angiogenic stimulation ( b) on **(I)** dosing administration, **(J)** drug concentration, and **(K)** treatment time.

The PF controller also revealed effective performance for multiple treatment scenarios (random combination of parameters) of small tumors. Moreover, 99.0% and 98.9% of treatments were able to reduce cancer volumes to <10 and <5 mm^3^, respectively, within 200 days of treatment and according to Bessel-like patterns, ensuring that the maximum drug administration was lower than 3 mg kg^-1^ (**Figure 4A**). The remaining treatments (≤1.1%) were converted into a chronic disease, as the dosing controller *attracted* a stationary (constant) tumor volume and vasculature capacity, thus stopping tumor growth dynamics. The maximum drug administration was 2.98 mg kg^-1^ (standard deviation: 4 × 10–^3^ mg kg^-1^), but the average drug administration did not exceed 1.26 mg kg^-1^ (standard deviation: 0.77 mg kg^-1^) throughout 10.3 days (**Figure 4A**), which resulted in maximum and average concentrations of 0.54 mg kg^-1^ (deviation: 0.20 mg kg^-1^) and 0.28 mg kg^-1^ (deviation: 0.18 mg kg^-1^), respectively (**Figure 4B**). The cytotoxic effect on the tumor volume (parameter φ), volumetric tumor growth (parameter ξ), and angiogenic stimulation (parameter b) slightly influenced the controller performance, including on their standard deviation. These parameters equally contributed to changing dose administrations and related drug concentrations and treatments (**Figures 4C–K**): (i) they increased the maximum drug administration from 2.98 to 3 mg kg^-1^ (**Figures 4C, F, I**) but (ii) reduced the treatment time from 10.3 days (deviation: 14.3 days) to 9.6 days (deviation: 7.3 days) (**Figures 4E, H, K**) and (iii) they changed the administration patterns from Bessel-like patterns to exponential-like patterns (**Figures 4C, D**) and diffusion-like patterns (**Figures 4F, G, I, J**). The PF controller also responded with diffusion-like control patterns to significant changes in the loss of endothelial support ( μ), cytotoxic effect on the vasculature ( η), and angiogenic inhibition ( d), requiring the same drug administration and treatment time, as shown in **Supplementary Figure 1A** (**Supplementary Information 3**). These different patterns (Bessel, exponential, and diffusion) converging to similar drug administration (maximum: 3 mg kg^-1^; average: 1.26 mg kg^-1^), similar drug concentrations (maximum: 0,54 mg kg^-1^; average: 0.28 mg kg^-1^), and similar treatment times (10 mm^3^: 6.3 days; 5 mm^3^: 9.6 days; remission states achieved in the range between 3.3 and 173.6 days of treatment) highlight the adaptability of the PF controller. **Table 3** provides a summary of these findings.

**Table 3:** Summary of the main findings related to the performance of the PF controller in randomized detumorization scenarios without chemoresistance.

Parameter	CP—SM				CP—LT			
	All	φ	ξ	b	All	φ	ξ	b
Maximum drug administration [mg kg^-1^]	2.98	3.00	3.00	3.00	3.00	3.00	3.00	3.00
Average drug administration [mg kg^-1^]	1.26	1.26	1.26	1.26	1.39	1.39	1.39	1.39
Maximum drug concentrations [mg kg^-1^]	0.54	0.54	0.54	0.54	0.53	0.53	0.53	0.53
Average drug concentrations [mg kg^-1^]	0.28	0.28	0.28	0.28	0.32	0.32	0.32	0.32
Average treatment time ( $10\;{\text{mm}}^{3})$ [days]	10.3	9.60	9.60	9.60	15.60	13.90	13.90	13.90

CP, cyclophosphamide; SM, smaller tumors ( $V_{0}=200\;{\text{mm}}^{3};\;R_{0}=625\;{\text{mm}}^{3}$); LT, larger tumors ( $V_{0}=2,000\;{\text{mm}}^{3}$ and $R_{0}=6,250\;{\text{mm}}^{3}$).

**Figure 5:**
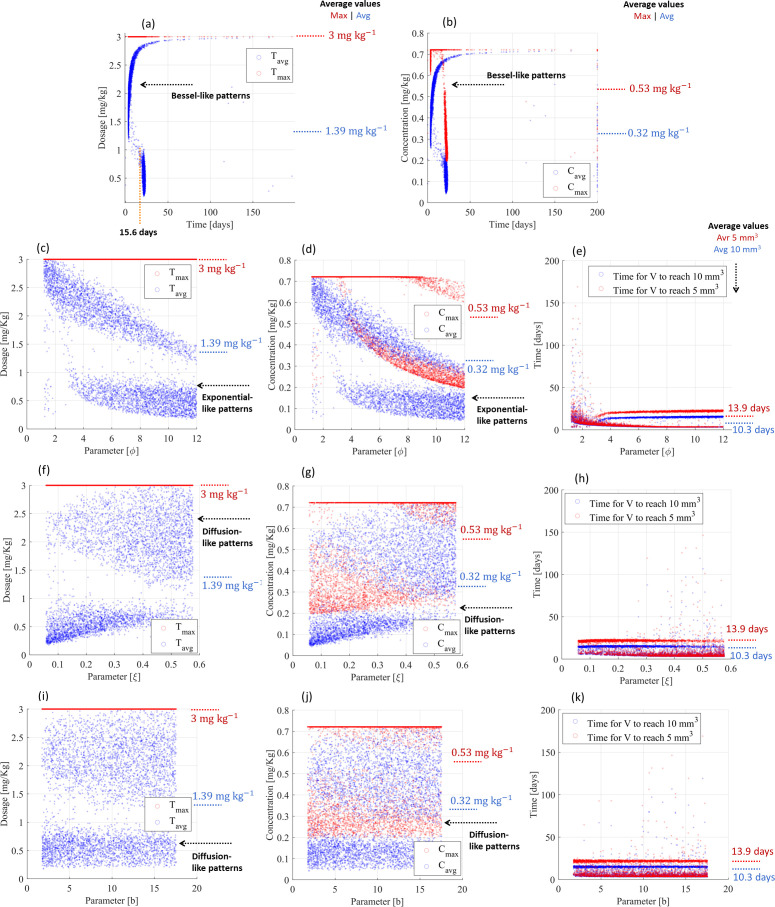
Resistance-free chemotherapy using cyclophosphamide for larger tumor scenarios using PF-inspired administration. **(A)** Dosing administration and **(B)** drug concentration considering the influence of all biological parameters. Influence of the cytotoxic effect on the tumor volume ( φ) on **(C)** dosing administration, **(D)** drug concentration, and **(E)** treatment time. Influence of the volumetric tumor growth ( ξ) on **(F)** dosing administration, **(G)** drug concentration, and **(H)** treatment time. Influence of the angiogenic stimulation ( b) on **(I)** dosing administration, **(J)** drug concentration, and **(K)** treatment time.

*PF-inspired administration considering the initial conditions* $\;(V_{0},R_{0})=(2,000,\;6,250)$ ${\text{mm}}^{3}$: Concerning the multiple treatment scenarios for larger tumors, the effectiveness was 99.0% (similar results for tumors <10 and <5 mm^3^) within 200 days of treatment and maximum drug administration of 3 mg kg^-1^ (**Figure 5A**); only 1% of treatments converged into a chronic disease. As expected, the average drug delivery increased (from 1.26 to 1.39 mg kg^-1^ (deviation: 0.85 mg kg^-1^)), as well as the average drug concentration (from 0.28 to 0.32 mg kg^-1^ (deviation: 0.19 mg kg^-1^)) and average treatment time (from 10.3 days to 15.6 days), as shown in **Figures 5A, B**. Bessel-like administration patterns were also observed. The PF controller was only slightly influenced by parameters φ, ξ, and b. These biological parameters did not influence the drug administration and related concentration (**Figures 5C, D, F, G, I, J**), and equally reduced the treatment time from 15.6 days (deviation: 20.4 days) to 13.9 days (deviation: 10.2 days), as highlighted in **Figures 5E, H, K**. Similar administration patterns were found for both small and large tumors (**Figures 5C, D, F, G, I, J**): exponential-like patterns for parameter φ (**Figure 4C, D**, **5C, D**), diffusion-like patterns for parameters ξ and b (**Figures 4F, G, I, J**, **5F, G, I, J**). Diffusion-like control patterns were also defined by the PF controller to ensure an adaptive behavior for variations in parameters μ, η, and d but required the same drug administration and treatment time (**Supplementary Figure 1B**). Besides that, the PF controller holds the potential to uniformize the treatment time: (i) the difference in the treatment times for small and large tumors (**Figures 4E, H, K**, **5E, H, K**) was 4.3 days (9.6 days for the smaller tumors; 13.9 days for the larger tumors) and (ii) for the difference in treatment time, it requires to decrease the tumor volume from 10 to 5 ${\text{mm}}^{3}$, which was 0.3 days (3.3 days for the smaller tumors; 3.6 days for the larger tumors). Notice that treatments between 3.2 days and 169 days were required such that remission states could be achieved, which are quite similar to those achieved for smaller tumors. The adaptability provided by the PF controller is evident from the observed control patterns, drug administrations, and treatment times. **Table 3** summarizes the findings described above.

**Figure 6:**
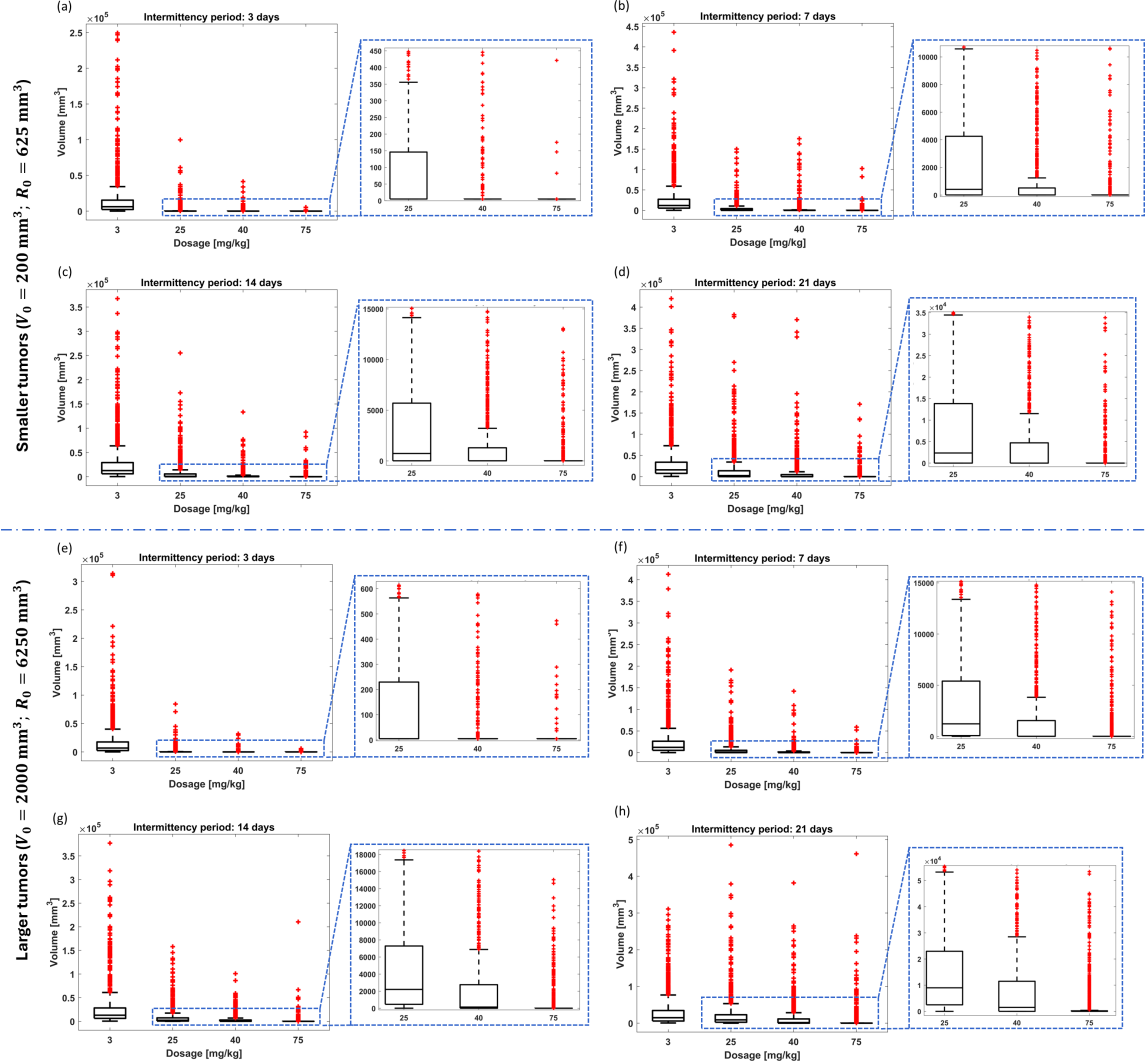
Resistance-free chemotherapy using cyclophosphamide for random combination of parameters of tumor dynamics and non-adaptive intermittent administration. Final tumor volume range for smaller tumors and intermittency period of **(A)** 3 days, **(B)** 7 days, **(C)** 14 days, and **(D)** 21 days. Final tumor volume range for larger tumors and intermittency period of **(E)** 3 days, **(F)** 7 days, **(G)** 14 days, and **(H)** 21 days. The bottom and top edges of the boxes indicate the 25th and 75th percentiles, respectively, while the marker “+” represents outliers.

**Figure 7:**
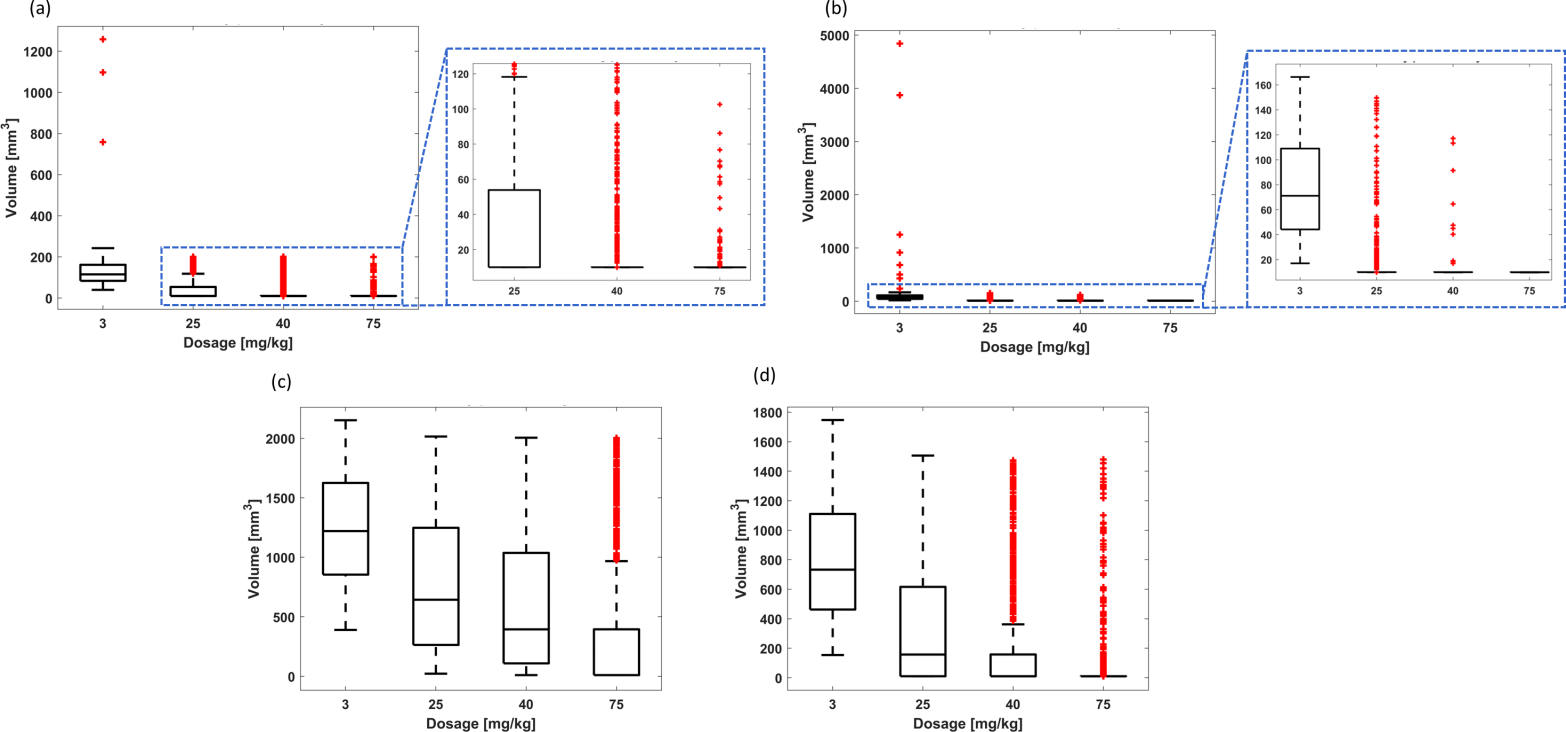
Resistance-free chemotherapy using cyclophosphamide for random combination of parameters of tumor dynamics and adaptive intermittent administration. Final tumor volume range considering the BB1 scheme for **(A)** smaller tumors and **(B)** larger tumors. Final tumor volume range considering the BB2 scheme for **(C)** smaller tumors and **(D)** larger tumors.

*Intermittent administration considering the initial conditions* $\left(V_{0},R_{0})=(200,625\right)$ ${\text{mm}}^{3}$: Both non-adaptive and adaptive intermittent administrations presented poor results compared to PF-inspired administration. Indeed while the PF controller ensured 89.9% of remission states using administrations lower than 3 mg kg^-1^ (average: 1.26 mg kg^-1^), the non-adaptive and adaptive intermittent administrations were not able to ensure effectiveness rates higher than 1% (**Tables 4**, **5**; **Figure 6**, **7**). Concerning non-adaptive fixed-dose intermittent administration (**Table 4**), only delivering 70 mg kg^-1^ on a 3-day intermittency basis can ensure tumor remission rates similar to those found in PF-inspired administration. If only 3 mg kg^-1^ is administered, the maximum expected effectiveness is 0.6% (3-day intermittency), and medium volumes are significantly larger than the initial ones. These metrics are improved when the administration is raised: Effectiveness rates are expected to be between 39.3% (21-day intermittency) and 69.6% (3-day intermittency) for 25 mg kg^-1^ and between 65.6% (21-day intermittency) and 86.1% (3-day intermittency) for 40 mg kg^-1^, but medium volumes can converge to clinically intolerable values. Finally, effectiveness rates in the range between 89.4% and 98.8% can be reached by 70 mg kg^-1^, but usually requiring longer treatment times in comparison to PF-inspired administration. The adaptive administration obtained enhanced performances compared to the non-adaptive administration, but it requires much higher drug administration (**Tables 4**, **5**). However, only the administration schemes using 70 mg kg^-1^ were able to overcome the effectiveness rates reached by the PF-inspired administration (**Table 5**). Indeed 95% and 100% were reached by BB1 and BB2 schemes, respectively, although these require median administrations between 20.75 and 43.70 mg kg^-1^. By administering 3.73 mg kg^-1^, the BB1 scheme achieves an effectiveness rate of only 52.8%. Even for remission rates between 52.8% and 95%, the BB1 scheme requires the administration of 3.73 and 20.75 mg kg^-1^, respectively, and the BB1 scheme requires administrations exceeding 12.2 mg kg^-1^ to obtain effectiveness rates higher than 90.1%.

**Table 4:** Summary of the results from non-adaptive intermittent administration treating the smaller tumors without chemoresistance.

*TD*	Results	$T_{INT}+t_{i}$			
		3 days	7 days	14 days	21 days
3 mg kg^-1^	Effectiveness (%)	0.6	0.0	0.0	0.0
	Treatment times (days)	48.4 to 189.5	-–	-–	-–
	Median volume (mm^3^)	6.2×10^3^	11.9×10^3^	12.6×10^3^	16.0×10^3^
	Volume range (mm^3^)	5.0 to 2.5×10^5^	124.1 to 4.3×10^5^	267.8 to 3.7×10^5^	507.1 to 4.2×10^5^
25 mg kg^-1^	Effectiveness (%)	69.6	43.0	39.3	39.3
	Treatment times (days)	0.2 to 144.4	0.2 to 77.6	0.2 to 28.8	0.2 to 0.7
	Median volume (mm^3^)	5.0	411.4	727.3	2.4×10^3^
	Volume range (mm^3^)	4.9 to 99.8×10^3^	4.9 to 1.5×10^5^	4.9 to 2.6×10^5^	4.9 to 3.8×10^5^
40 mg kg^-1^	Effectiveness (%)	86.1	69.0	69.2	65.6
	Treatment times (days)	0.2 to 195.6	0.2 to 42.5	0.2 to 56.9	0.2 to 0.9
	Median volume (mm^3^)	5.0	5.0	5.0	5.0
	Volume range (mm^3^)	4.9 to 41.4×10^3^	4.9 to 1.8×10^5^	4.9 to 1.3×10^5^	4.9 to 3.7×10^5^
70 mg kg^-1^	Effectiveness (%)	98.8	90.1	88.4	89.4
	Treatment times (days)	0.1 to 78.6	0.1 to 56.7	0.1 to 70.8	0.1 to 0.7
	Median volume (mm^3^)	4.9	4.9	4.9	4.9
	Volume range (mm^3^)	4.7 to 5.3×10^3^	4.7 to 1.0×10^5^	4.7 to 0.9×10^5^	4.7 to 1.7×10^5^

Effectiveness refers to the percentage of treatments that achieved a remission state; treatment times refer only to those treatments that reached a remission state. Volumetric metrics refer to all tumor dynamics defined by random combinations of model parameters.

TD, tolerated dose; $T_{INT}+t_{i}$, intermittency period.

**Table 5:** Summary of the results from adaptive intermittent administration without chemoresistance.

TD	Results	CP—SM		CP—LT	
		BB1	BB2	BB1	BB2
3 mg kg^-1^	Effectiveness (%)	0.0	0.0	0.0	0.0
	Treatment times (days)	-–	-–	-–	-–
	Median volume (mm^3^)	115.0	71.2	1220.3	732.7
	Volume range (mm^3^)	39.7 to 1,258.1	17.1 to 4,841.1	389.7 to 2,151.9	153.0 to 1,747.1
	Median administration (mg kg^-1^)	0.67	0.71	0.42	0.49
25 mg kg^-1^	Effectiveness (%)	52.8	90.1	0.0	28.3
	Treatment times (days)	0.3 to 0.9	0.2 to 0.9	-–	0.4 to 1.0
	Median volume (mm^3^)	0.2 to 6.4	10.0	643.4	157.2
	Volume range (mm^3^)	10.0	9.8 to 149.7	21.8 to 2,015.0	9.9 to 1,506.1
	Median administration (mg kg^-1^)	3.73	12.2	0.56	2.23
40 mg kg^-1^	Effectiveness (%)	77.8	99.0	6.1	61.3
	Treatment times (days)	0.2 to 6.4	0.1 to 0.9	0.5 to 63.9	0.3 to 0.9
	Median volume (mm^3^)	9.9 to 200.8	9.9	394.4	10.0
	Volume range (mm^3^)	9.9 to 200.5	9.8 to 117.1	10.0 to 2,005.1	9.8 to 1,473.3
	Median administration (mg kg^-1^)	8.62	22.0	0.79	7.19
70 mg kg^-1^	Effectiveness (%)	95.0	100.0	53.2	87.2
	Treatment times (days)	0.1 to 2	0.1 to 0.5	0.2 to 39.4	0.2 to 1.1
	Median volume (mm^3^)	9.9	9.9	10.0	9.9
	Volume range (mm^3^)	9.8 to 200.3	9.6 to 10.0	9.9 to 2,003.0	9.7 to 1,479.7
	Median administration (mg kg^-1^)	20.75	43.70	5.58	20.6

Effectiveness refers to the percentage of treatments that achieved a remission state; treatment times refer only to those treatments that reached a remission state. Volumetric metrics refer to all tumor dynamics defined by random combinations of model parameters.

TD, tolerated dose; BB1, $L_{Sup}=V_{0}$ and $L_{Inf}=\frac{V_{0}\;}{2}$; BB2, $L_{Sup}=\frac{3V_{0}}{4}$ and $L_{Inf}=\frac{V_{0}\;}{4}.$

*Intermittent administration considering the initial conditions* $\left(V_{0},R_{0})=(2,000,\;6,250\right)$ ${\text{mm}}^{3}$: Similar findings were found when comparing the performance of the three administration methods considered in this study (**Tables 5**, **6**). The PF-inspired administration was able to provide geometro-pharmacokinetics that outperformed both the non-adaptive and adaptive intermittent administrations. Indeed while the PF controller required an average administration of 1.39 mg kg^-1^ to achieve 99% effectiveness, the non-adaptive fixed-dose administration required the delivery of 70 mg kg^-1^ on a 3-day intermittency to ensure a closer tumor remission rate (98%) (**Table 5**), and the BB2 adaptive administration required 20.6 mg kg^-1^ to achieve 87.2% (the highest effectiveness rate) (**Table 6**). The effectiveness rates of non-adaptive fixed-dose intermittent administration (**Table 6**) were found between 4.5% and 65.7% for 25 mg kg^-1^, 42.4% and 86.5% for 40 mg kg^-1^, and 74.5% to 98% for 75 mg kg^-1^ and required longer treatment times in comparison to PF-inspired administration. Concerning the effectiveness rates of adaptive intermittent schemes (**Table 5**), the administration of 2.23 mg kg^-1^ resulted in an effectiveness of only 28.3% (BB2 scheme); enhanced results are expected to require significantly higher dosages: 61.3% for 7.19 mg kg^-1^ (BB2 scheme) and 87.2% for 20.6 mg kg^-1^ (BB2 scheme). The BB1 scheme was not able to exceed 53.2%.

**Table 6:** Summary of the results from non-adaptive intermittent administration treating larger tumors without chemoresistance.

*TD*	Results	$T_{INT}+t_{i}$			
		3 days	7 days	14 days	21 days
3 mg kg^-1^	Effectiveness (%)	0.0	0.0	0.0	0.0
	Treatment times (days)	-–	-–	-–	-–
	Median volume (mm^3^)	6.8×10^3^	12.2×10^3^	12.9×10^3^	14.9×10^3^
	Volume range (mm^3^)	6.8 to 3.1×10^5^	82.5 to 4.1×10^5^	384.7 to 3.8×10^5^	513.7 to 3.1×10^5^
25 mg kg^-1^	Effectiveness (%)	65.7	24.1	7.2	4.5
	Treatment times (days)	0.5 to 168.6	0.5 to 133.7	0.5 to 112.8	0.5 to 84.9
	Median volume (mm^3^)	5.0	1225.7	2204.0	8993.1
	Volume range (mm^3^)	4.9 to 84.2×10^3^	4.9 to 1.9×10^5^	4.9 to 1.6×10^5^	4.9 to 4.8×10^5^
40 mg kg^-1^	Effectiveness (%)	86.5	56.1	45.9	42.4
	Treatment times (days)	0.2 to 177.7	0.2 to 161.7	0.2 to 154.8	0.2 to 63.6
	Median volume (mm^3^)	5.0	5.0	5.0	1513.7
	Volume range (mm^3^)	4.9 to 31.8×10^3^	4.9 to 1.4×10^5^	4.9 to 1.0×10^5^	4.9 to 3.8×10^5^
70 mg kg^-1^	Effectiveness (%)	98.0	84.2	80.2	74.5
	Treatment times (days)	0.1 to 171.6	0.1 to 133.7	0.1 to 154.8	0.1 to 63.8
	Median volume (mm^3^)	4.9	4.9	4.9	4.9
	Volume range (mm^3^)	4.7 to 5.8×10^3^	4.7 to 0.6×10^3^	4.7 to 2.1×10^5^	4.7 to 4.6×10^5^

Effectiveness refers to the percentage of treatments that achieved a remission state; treatment times refer only to those treatments that reached a remission state. Volumetric metrics refer to all tumor dynamics defined by random combinations of model parameters.

TD, tolerated dose; $T_{INT}+t_{i}$, intermittency period.

### 3.2 Results for cancer therapy with chemoresistance

#### 3.2.1 Stability of the PF controller

Similarly to the resistance-free chemotherapy, a remission state is reached when $(N_{1}+N_{1})=0$ for chemotherapy with chemoresistance, respectively; therefore, remission states are unique and equilibrium solutions to the dosing control problem. We must then analyze whether the PF controller is able to provide unique treatments, which are the equilibrium tumor states (for both regions where r>R and |r|≤R), which conditions are required by the controller to ensure the stabilization of such equilibrium states. A linear approximation of tumor dynamics related to chemoresistance treatments (nonlinear system expressed by (Equation 2) was also carried out to simplify the stability analysis of detumorization trajectories. The same assumptions considered for resistance-free chemotherapy were also considered for chemoresistance treatments (Section 3.1.1) and their non-significant impact in the validity of stability conditions. We found that chemoresistance mechanisms can be managed by the PF dosing controller. We also show that effective drug delivery dynamics can be forced by this controller, such that stable detumorizations can be ensured toward remission states ( V→0 as r→0), and these equilibrium states can be kept. The analytical proof is provided in Appendix B.

#### 3.2.2 *In silico* results

**Figure 8:**
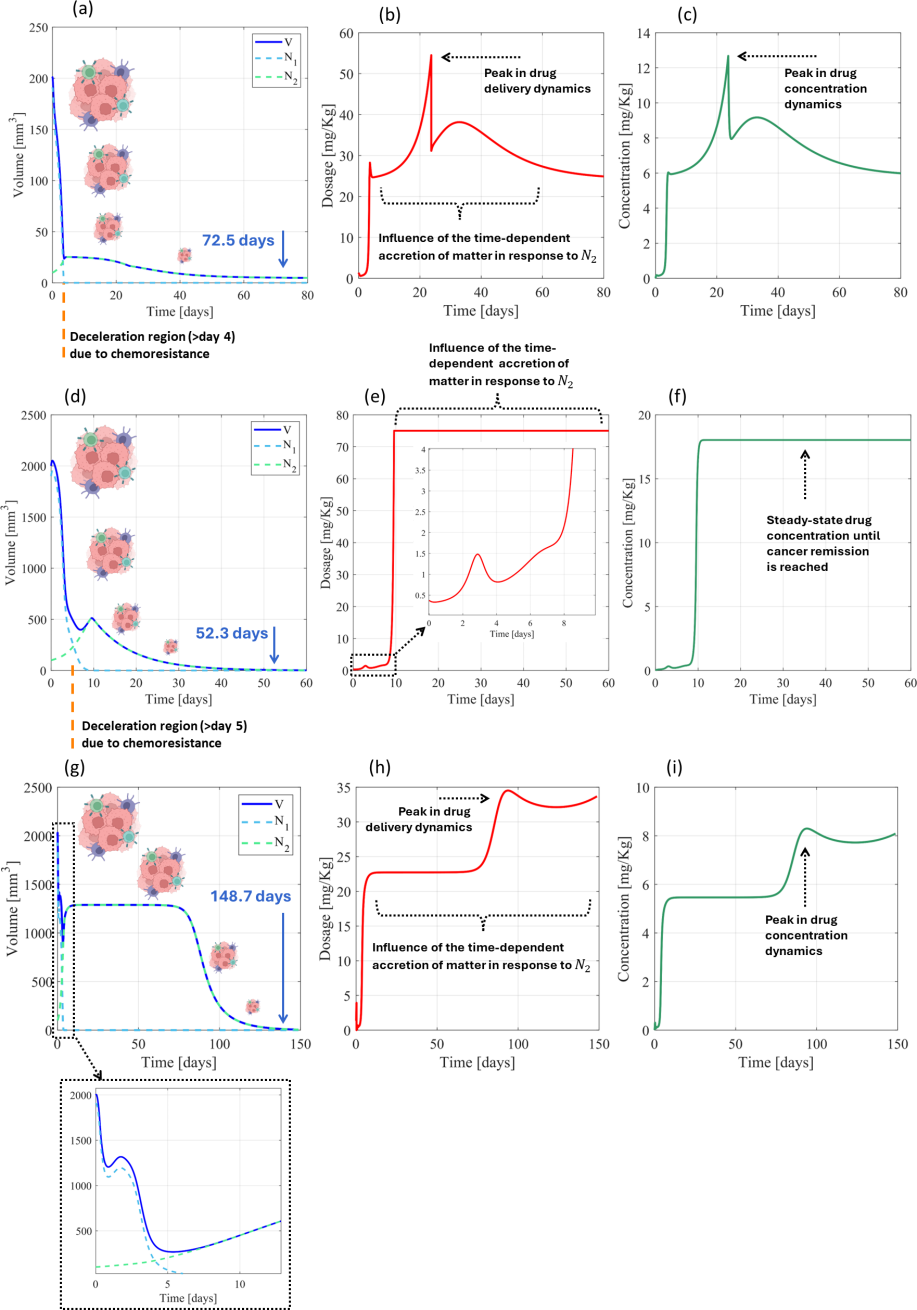
Cancer therapy with chemoresistance using cyclophosphamide for PF-inspired administration. **(A)** Volume dynamics, **(B)** drug administration dynamics, and **(C)** drug concentration dynamics for the smaller tumor scenario (primary parametrization). **(D)** Volume dynamics, **(E)** drug administration dynamics, and **(F)** drug concentration dynamics for the larger tumor scenario (primary parametrization). Performance of the PF controller (parameters CP—LT from **Table 2**) for tumor growth dynamics expressed by $\varphi =11.7819\;{\text{conc}}^{-1}{\text{ day}}^{-1}$, $\eta =2.8187\;{\text{conc}}^{-1}{\text{ day}}^{-1}$, $\xi_{1}=0.5412$, $\xi_{2}=0.2706{\text{ day}}^{-1}$, $\tau_{1}=5.6374\;\times \;10^{-5}$ ${\text{day}}^{-1}$, $\tau_{2}=2.8187\;\times \;10^{-5}$ ${\text{day}}^{-1},\;b=16.4894{\text{ day}}^{-1}$, $d=0.0246{\text{ mm}}^{-2}{\text{day}}^{-1}$, and $\mu =0.0564\;{\text{day}}^{-1}$. **(G)** Volume dynamics, **(H)** drug administration dynamics, and **(I)** drug concentration dynamics for the smaller tumor scenario. **Figures 6A, D, G** were created using a picture from BioRender, Gonçalves, **(G)** (2026); https://BioRender.com/u0ys9z9.

*PF-inspired administration considering the initial conditions* $\left(N_{10},N_{20},R_{0})=(190,\;10,\;625\right)$ ${\text{mm}}^{3}$: Using the tumor primary parameters for the smaller tumor scenario, 72.5 days were required by the PF controller to perform the detumorization from initial tumor volumes up to 5 $\;{\text{mm}}^{3}$ (**Figure 8A**) by delivering a nonlinear dosing dynamics characterized by 54.50 and 29.58 mg kg^-1^ of maximum and average drug administration, respectively, and 12.68 and 7.07 mg kg^-1^ of maximum and average concentrations (**Figures 8B, C**), which provided a smooth detumorization dynamics as V→0 (**Figure 8A**). This tumor regression is characterized by a deceleration region starting at day 4, when the tumor volume is 25.3 ${\text{mm}}^{3}$, which occurred due to the emergence of resistant tumor phase $N_{2}$. This chemoresistance lasted 68.5 days to be significantly reduced to a remission state, requiring a control signal highly influenced by the time-dependent accretion of matter $e^{k_{1}{\left(\int_{0}^{t}r\;d\tau \right)}^{2}}$, with $k_{1}=k_{m}t$) (Equation 4), which naturally produces a slow detumorization dynamics. This treatment phenomenon differs from the drug-resistance-free treatment scenarios; indeed 4.4 days were required for resistance-free chemotherapy to reach 25.3 ${\text{mm}}^{3}$ (**Figure 3A**), and 4 days were required for chemotherapy throughout negligible chemoresistance (**Figure 8A**). Concerning the larger tumor scenario, a highly nonlinear drug dynamics was also imposed by the controller to ensure a reduction in cancer volumes to <5 mm^3^ (**Figures 8D, E**), in which less than 2 mg kg^-1^ was delivered until day 8 (tumor volume: 432 mm^3^), followed by a sharp increase to effectively respond to the rapid increase of the resistant tumor phase, remaining in a steady-state drug administration until cancer remission is reached (**Figure 8D**). This dynamic dosage (average: 63.56 mg kg^-1^) was able to force a detumorization dynamics that reached a tumor remission 20.2 days sooner than for the lower tumors (**Figure 8D**) but requiring an increased average drug concentration (from 7.07 to 15.21 mg kg^1^). Such steady-state drug delivery is not the only response provided by the PF controller. Indeed different tumor growth dynamics will be treated with customized adaptive patterns, such as the one presented in **Figures 8G–I** that introduces a constant drug administration throughout 46 days (between days 14 and 60), and then interrupted by an adequate increase in drug release (from 23 to 34.5 mg kg^-1^) controlled by the time-dependent accretion of matter.

**Figure 9:**
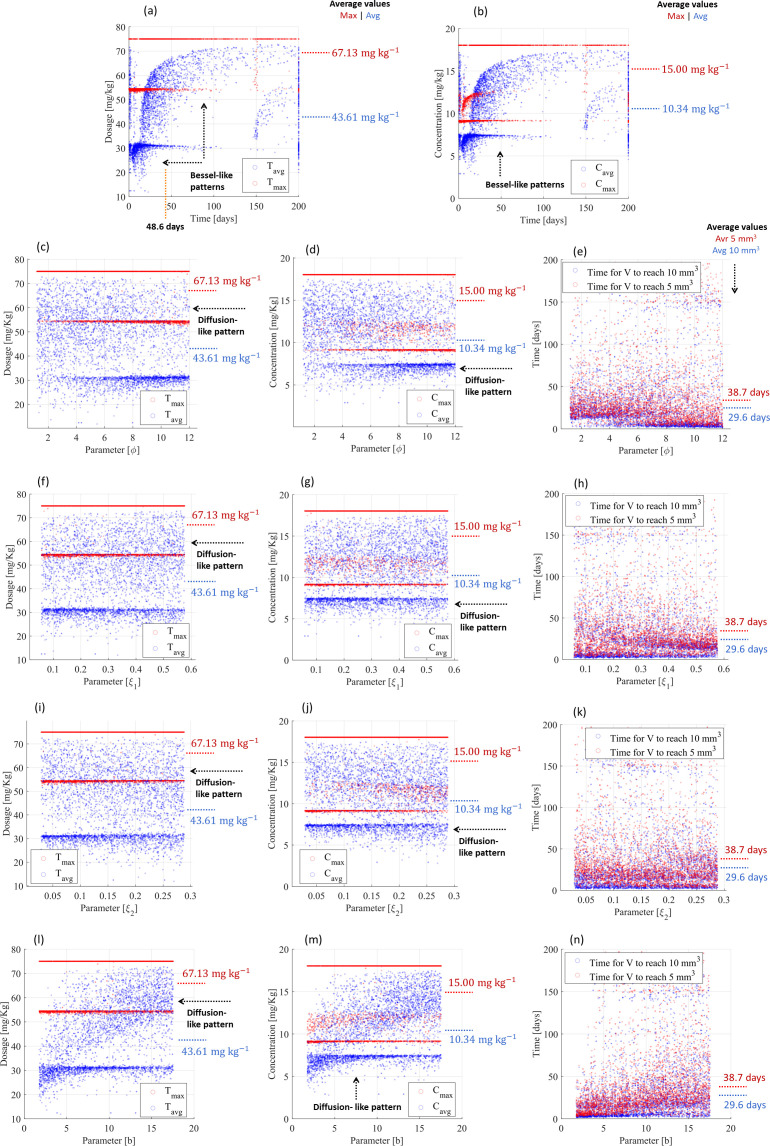
Cancer therapy with chemoresistance using cyclophosphamide for smaller tumor scenarios and PF-inspired administration. **(A)** Dosing administration and **(B)** drug concentration considering the influence of all biological parameters. Influence of the cytotoxic effect on the tumor volume ( φ) on **(C)** dosing administration, **(D)** drug concentration, and **(E)** treatment time. Influence of the volumetric tumor growth ( $\xi_{1}$) related to sensitive tumor tissue on **(F)** dosing administration, **(G)** drug concentration, and **(H)** treatment time. Influence of the volumetric tumor growth ( $\xi_{2}$) related to resistant tumor tissue on **(I)** dosing administration, **(J)** drug concentration, and **(K)** treatment time. Influence of the angiogenic stimulation ( b) on **(L)** dosing administration, **(M)** drug concentration, and **(N)** treatment time.

Concerning multiple treatment scenarios for small tumors, >80% of treatments resulted in complete tumor remission (87.3% for cancer volumes <10 mm^3^; 80.2% for <5 mm^3^) for maximum drug administration <75 mg kg^-1^, delivered throughout 200 days of treatment (**Figure 9A**), and the remaining ones (<20%) reach tumor retention states. The maximum drug administration was 67.13 mg kg^-1^ (standard deviation: 10.04 mg kg^-1^), but the average drug administration did not exceed 43.61 mg kg^-1^ (standard deviation: 14.02 mg kg^-1^) throughout 48.6 days (**Figure 9A**), which resulted in maximum and average concentrations of 15 mg kg^-1^ (deviation: 3.95 mg kg^-1^) and 10.34 mg kg^-1^ (deviation: 3.4 mg kg^-1^), respectively (**Figure 9B**). Therefore, chemoresistance required 22.5- and 34.6-fold increases in the maximum and average doses, respectively, in comparison with resistance-free chemotherapy. Highly nonlinear control patterns were defined by the PF controller, expressed by Bessel-like patterns considering the influence of all biological parameters (**Figures 9A, B**), and diffusion-like patterns for all individual influences (cytotoxic effect on the tumor volume ( φ), **Figures 9C, D**; volumetric tumor growth ( $\xi_{1}$) related to sensitive tumor tissue, **Figures 9F, G**; volumetric tumor growth ( $\xi_{2}$) related to resistant tumor tissue, **Figures 9I, J**; angiogenic stimulation ( b), **Figures 9L, M**; cellular mutation rate ( $\tau_{1}$), **Supplementary Figures 3A, B**; cellular reverse mutation rate ( $\tau_{2}$), **Supplementary Figures 3D, E**; the loss of endothelial support ( μ), **Supplementary Figures 3G, H**; cytotoxic effect on the vasculature ( η), **Supplementary Figures 3J, K**; angiogenic inhibition ( d), **Supplementary Figures 3M, N**). Even though different diffusion-like patterns were established, similar drug administrations (maximum: 67.13 mg kg^-1^; average: 43.61 mg kg^-1^) and drug concentrations (maximum: 15 mg kg^-1^; average: 10.34 mg kg^-1^) were required. These control patterns provide strong evidence that PF dosing control ensures adaptive behaviors for uncertain tumor dynamics. A relevant uniformity between the treatment times was also found; indeed remission states were achieved in the range between 2.5 and 196.9 days of treatment, which differed by only 13.4% (the maximum treatment time) from the range obtained in resistance-free chemotherapy. **Table 4** provides a summary of these findings.

**Figure 10:**
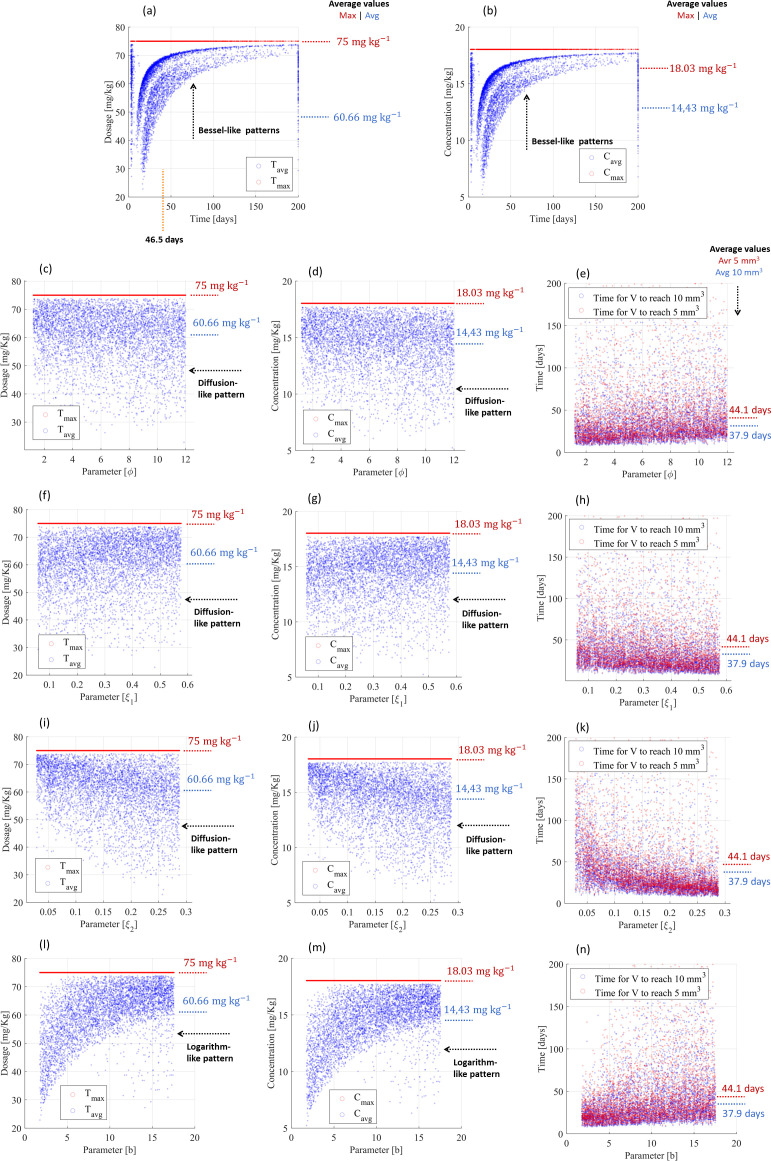
Cancer therapy with chemoresistance using cyclophosphamide for larger tumor scenarios and PF-inspired administration. **(A)** Dosing administration and **(B)** drug concentration considering the influence of all biological parameters. Influence of the cytotoxic effect on the tumor volume ( φ) on **(C)** dosing administration, **(D)** drug concentration, and **(E)** treatment time. Influence of the volumetric tumor growth ( $\xi_{1}$) related to sensitive tumor tissue on **(F)** dosing administration, **(G)** drug concentration, and **(H)** treatment time. Influence of the volumetric tumor growth ( $\xi_{2}$) related to resistant tumor tissue on **(I)** dosing administration, **(J)** drug concentration, and **(K)** treatment time. Influence of the angiogenic stimulation ( b) on **(L)** dosing administration, **(M)** drug concentration, and **(N)** treatment time.

*PF-inspired administration considering the initial conditions* $\left(N_{10},N_{20},R_{0})=(1,900,\;100,\;6,250\right)$ ${\text{mm}}^{3}$: Concerning multiple treatment scenarios for large tumors, only 13.7% of treatment converged for a chronic disease after 200 days of treatment (87.2% for cancer volumes <10 mm^3^; 86.3% for cancer volumes <5 mm^3^), which is surprisingly lower than the achievements for smaller tumors. Compared with treatments for small tumors, both the maximum and average drug delivery increased (maximum: from 67.13 mg kg^-1^ to 75 mg kg^-1^ (deviation: 0 mg kg^-1^), corresponding to 11.7% higher peak dosing; average: from 43.61 mg kg^-1^ to 60.66 mg kg^-1^ (deviation: 9.26 mg kg^-1^), corresponding to 39.1% higher average dosing) (**Figure 8A**, **9A**), as well as the average drug concentration (maximum: from 15 mg kg^-1^ to 18.03 mg kg^-1^ (deviation: 0 mg kg^-1^); average: from 10.34 mg kg^-1^ to 14.43 mg kg^-1^ (deviation: 2.28 mg kg^-1^)) (**Figure 8B**, **9B**). However, the average treatment time was also surprisingly reduced by 2.1 days (from 48.6 to 46.5 days), as shown in **Figure 8A**, **9A**, being reduced by only 2.5 days under the influence of individual biological parameters (**Figures 10E, H, K, N**; **Supplementary Figures 4C, F, I, L, O**). Most biological parameters were controlled using the diffusion-like control patterns, namely, $\varphi ,\xi_{1}$, $\xi_{2},$ $\tau_{1}$, $\tau_{2}$, μ, η, and d (**Figures 10C, D, F, G, I, J, L, M**; **Supplementary Figures 4A, B, D, E, G, H, J, K, M, N**). Nevertheless, the angiogenic stimulation ( b) was controlled by a logarithm-like control pattern, which highlights the adaptive behavior of this new control method. Even though the PF controller demanded 25- and 43.6-fold increases in the maximum and average doses, respectively, in comparison with resistance-free chemotherapy, it expressed once again its potential to uniformize the treatment time. Indeed the difference in the treatment times for small and large tumors (**Figures 9E, H, K, N**, **10E, H, K, N**) was 5.4 days (38.7 days for the smaller tumors; 44.1 days for the larger tumors), which was slightly higher than that obtained in resistance-free chemotherapy (**Figures 5E, H, K**). In addition, the difference in treatment time for decreasing the tumor volume from 10 to 5 ${\text{mm}}^{3}$ (**Figures 9E, H, K, N**, **10E, H, K, N**) was 2.9 days (9.1 days for the smaller tumors; 6.2 days for the larger tumors), which was slightly shorter than that obtained in resistance-free treatments (**Figures 5E, H, K**). Moreover, remission states were achieved in the range between 8.9 and 199.7 days of treatment (**Figures 10E, H, K, N**), an increase of only 1.4% (the maximum treatment time) compared with the range obtained for treating small tumors with chemoresistance (**Figures 9E, H, K, N**). **Table 7** summarizes the findings described above.

**Table 7:** Summary of the main findings related to the performance of the PF controller in randomized detumorization scenarios with chemoresistance.

Parameter	CP—SM					CP—LT				
	All	φ	$\xi_{1}$	$\xi_{2}$	b	All	φ	$\xi_{1}$	$\xi_{2}$	b
Maximum drug administration [mg kg^-1^]	67.13	67.13	67.13	67.13	67.13	75	75	75	75	75
Average drug administration [mg kg^-1^]	43.61	43.61	43.61	43.61	43.61	60.66	60.66	60.66	60.66	60.66
Maximum drug concentrations [mg kg^-1^]	15.00	15.00	15.00	15.00	15.00	18.03	18.03	18.03	18.03	18.03
Average drug concentrations [mg kg^-1^]	10.34	10.34	10.34	10.34	10.34	14.43	14.43	14.43	14.43	14.43
Average treatment time ( $10\;{\text{mm}}^{3})$ [days]	48.6	38.7	38.7	38.7	38.7	46.5	44.1	44.1	44.1	44.1

CP, cyclophosphamide; SM, smaller tumors ( $V_{0}=200\;{\text{mm}}^{3};\;R_{0}=625\;{\text{mm}}^{3}$); LT, larger tumors ( $V_{0}=2,000\;{\text{mm}}^{3}$ and $R_{0}=6,250\;{\text{mm}}^{3}$).

**Figure 11:**
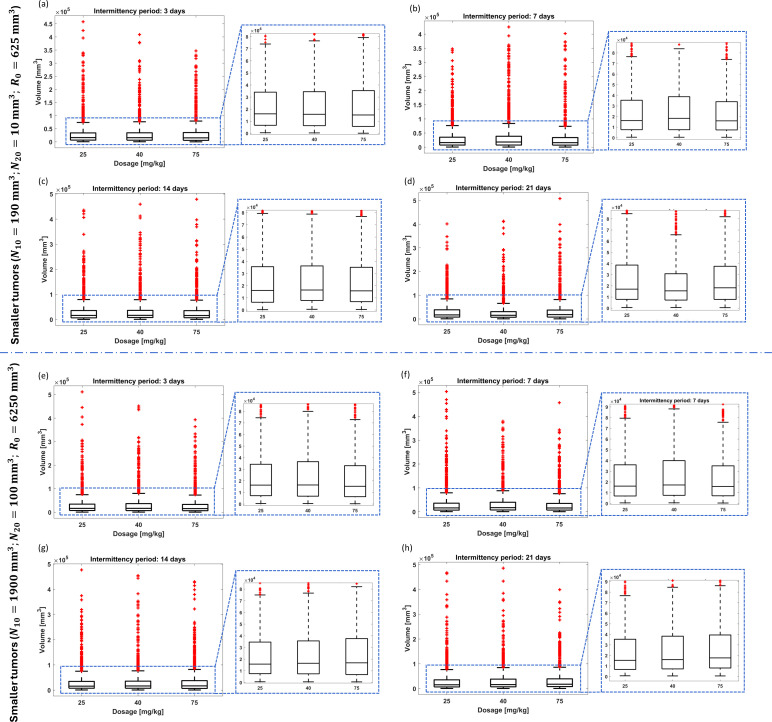
Cancer therapy with chemoresistance using cyclophosphamide for the random combination of parameters of tumor dynamics and non-adaptive intermittent administration. Final tumor volume range for smaller tumors and intermittency period of **(A)** 3 days, **(B)** 7 days, **(C)** 14 days, and **(D)** 21 days. Final tumor volume range for larger tumors and intermittency period of **(E)** 3 days, **(F)** 7 days, **(G)** 14 days, and **(H)** 21 days. The bottom and top edges of the boxes indicate the 25th and 75th percentiles, respectively, while the marker “+” represents outliers.

**Figure 12:**
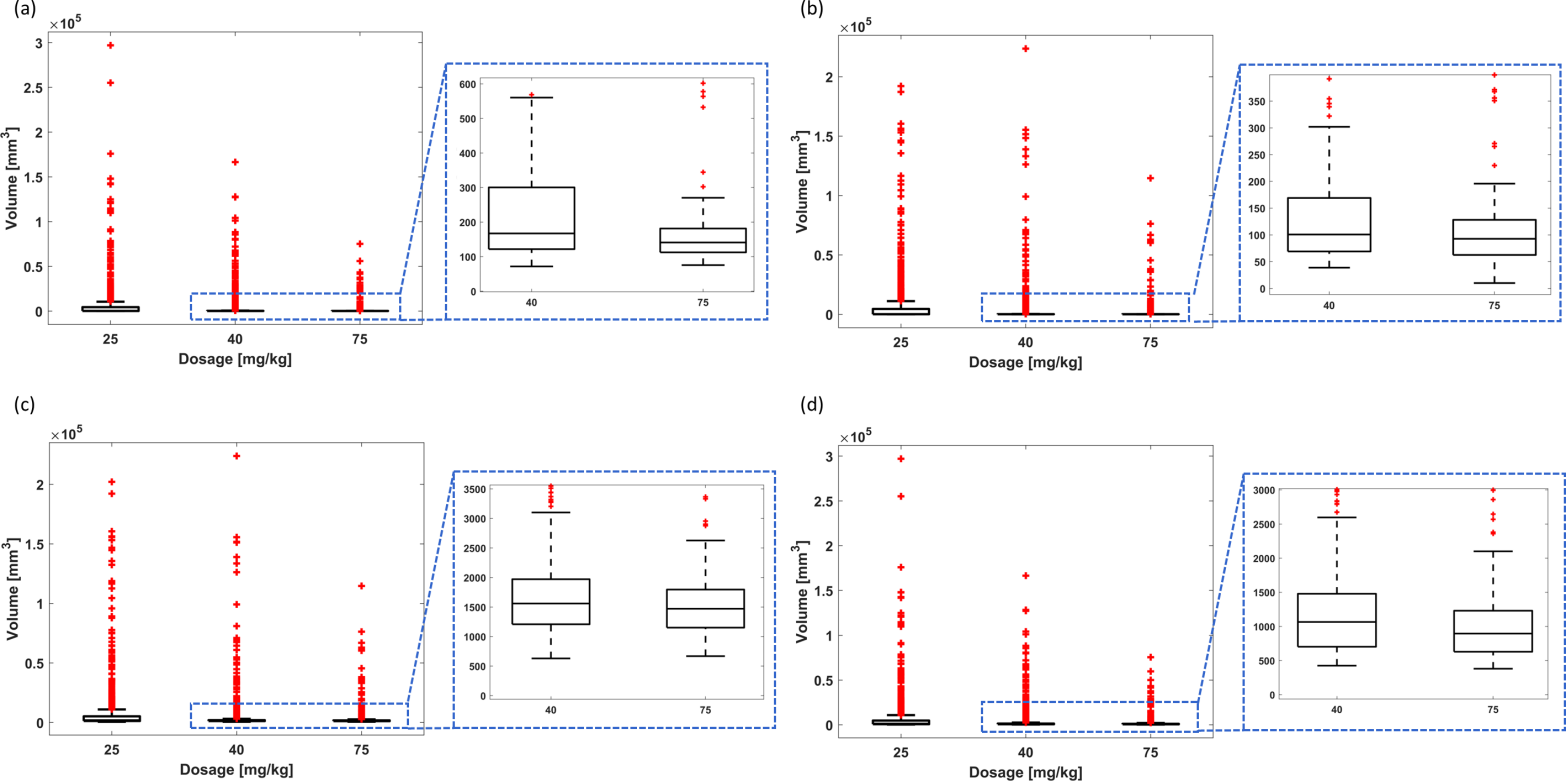
Cancer therapy with chemoresistance using cyclophosphamide for the random combination of parameters of tumor dynamics and adaptive intermittent administration. Final tumor volume range considering the BB1 scheme for **(A)** smaller tumors and **(B)** larger tumors. Final tumor volume range considering the BB2 scheme for **(C)** smaller tumors and **(D)** larger tumors.

*Intermittent administration considering the initial conditions* $\left(N_{10},N_{20},R_{0})=(190,\;10,\;625\right)$ ${\text{mm}}^{3}$: The PF-inspired administration significantly outperformed both the non-adaptive and adaptive intermittent administrations. While the PF controller allows cyclophosphamide to be administered as a monotherapy, achieving effectiveness rates of 87.3% using 43.61 mg kg^-1^ (**Table 7**), such is an ability that neither the non-adaptive nor the adaptive administration can replicate. Indeed intermittent drug delivery is not capable of detumorization dynamics ensuring remission states: the effectiveness is always zero, except the BB2 scheme for 70 mg kg^-1^, which showed 0.1% (**Tables 8**, **9**; **Figures 11**, **12**). Concerning the non-adaptive fixed-dose administration, median final tumor volumes exceeded the initial volume by >77 times (**Table 8**). The enhanced results were obtained by adaptive administration: the final tumor volumes were between 92.7 mm^3^ (corresponding to 0.46 times the initial volume using the BB2 scheme) and 195.4 mm^3^ (corresponding to 0.98 times the initial volume using the BB1 scheme), as shown in **Table 9**. Moreover, the lowest tumor volume (92.7 mm^3^) was achieved with an average administration of 46.86 mg kg^-1^, which is superior to the administration required by the PF controller to achieve the effectiveness rate of 87.3%.

**Table 8:** Summary of the results from non-adaptive intermittent administration treating the smaller tumors with chemoresistance.

*TD*	Results	$T_{INT}+t_{i}$			
		3 days	7 days	14 days	21 days
25 mg kg^-1^	Effectiveness (%)	0.0	0.0	0.0	0.0
	Treatment times (days)	-–	-–	-–	-–
	Median volume (mm^3^)	16.1×10^3^	16.1 ×10^3^	16.1×10^3^	17.1×10^3^
	Volume range (mm^3^)	461.5 to 4.6×10^5^	510.5 to 3.5×10^5^	298.5 to 4.4×10^5^	584.3 to 4.0×10^5^
40 mg kg^-1^	Effectiveness (%)	0.0	0.0	0.0	0.0
	Treatment times (days)	-–	-–	-–	-–
	Median volume (mm^3^)	15.8×10^3^	18.3×10^3^	16.4×10^3^	15.5×10^3^
	Volume range (mm^3^)	340.8 to 4.1×10^5^	475.3 to 4.3×10^5^	591.2 to 4.6×10^5^	594.8 to 4.1×10^5^
70 mg kg^-1^	Effectiveness (%)	0.0	0.0	0.0	0.0
	Treatment times (days)	-–	-–	-–	-–
	Median volume (mm^3^)	15.2×10^3^	15.9×10^3^	15.7×10^3^	18.3×10^3^
	Volume range (mm^3^)	102.6 to 3.5 ×10^5^	450.9 to 4.0×10^5^	423.7 to 4.8×10^5^	482.1 to 5.1×10^5^

Effectiveness refers to the percentage of treatments that achieved a remission state; treatment times refer only to those treatments that reached a remission state. Volumetric metrics refer to all tumor dynamics defined by random combinations of model parameters.

TD, tolerated dose; $T_{INT}+t_{i}$, intermittency period.

**Table 9:** Summary of the results from adaptive intermittent administration with chemoresistance.

*TD*	Results	CP—SM		CP—LT	
		BB1	BB2	BB1	BB2
25 mg kg^-1^	Effectiveness (%)	0.0	0.0	0.0	0.0
	Treatment times (days)	-–	-–	-–	-–
	Median volume (mm^3^)	195.4	146.0	1,780.2	1,246.4
	Volume range (mm^3^)	85.3 to 3.0×10^5^	43.2 to 1.9×10^5^	609.9 to 2.0×10^5^	394.4 to 3.0×10^5^
	Median administration (mg kg^-1^)	20.21	20.59	17.41	18.80
40 mg kg^-1^	Effectiveness (%)	0.0	0.0	0.0	0.0
	Treatment times (days)	-–	-–	-–	-–
	Median volume (mm^3^)	167.2	100.8	1,559.4	1,065.5
	Volume range (mm^3^)	71.7 to 1.7×10^5^	38.8 to 2.2×10^5^	629.7 to 2.2×10^5^	425.4 to 1.6×10^5^
	Median administration (mg kg^-1^)	29.25	30.16	24.84	26.9
70 mg kg^-1^	Effectiveness (%)	0.0	0.1	0.0	0.0
	Treatment times (days)	-–	0.9	-–	-–
	Median volume (mm^3^)	141.1	92.7	1,470.7	895.5
	Volume range (mm^3^)	75.6 to 0.8×10^5^	10 to 1.1×10^5^	667.9 to 1.1×10^5^	380.9 to 7.6×10^4^
	Median administration (mg kg^-1^)	44.79	46.86	36.56	41.14

Effectiveness refers to the percentage of treatments that achieved a remission state; treatment times refer only to those treatments that reached a remission state. Volumetric metrics refer to all tumor dynamics defined by random combinations of model parameters.

CP, cyclophosphamide; SM, smaller tumors ( $V_{0}=200\;{\text{mm}}^{3};\;R_{0}=625\;{\text{mm}}^{3}$); LT, larger tumors ( $V_{0}=2,000\;{\text{mm}}^{3}$ and $R_{0}=6,250\;{\text{mm}}^{3}$).

*Intermittent administration considering the initial conditions* $\left(N_{10},N_{20},R_{0})=(1,900,\;100,\;6,250\right)$ ${\text{mm}}^{3}$: Similar findings were found when comparing the performance of the three administration methods considered in this study (**Tables 9**, **10**; **Figures 11**, **12**). The PF-inspired administration outperformed both the non-adaptive and adaptive intermittent administrations. Indeed while the PF controller required an average administration of 60.66 mg kg^-1^ to achieve the effectiveness rate of 87.2% (**Table 7**), the non-adaptive fixed-dose intermittent administration was not able to reduce the initial tumor volume (considering average metric; **Table 10**), and the adaptive intermittent administration was only able to reduce the initial tumor volume to 895.5 mg kg^-1^ (corresponding to 0.45 times the initial volume using the BB2 scheme), even requiring 41.14 mg kg^-1^ (**Table 9**).

**Table 10:** Summary of the results from non-adaptive intermittent administration treating the larger tumors with chemoresistance.

*TD*	Results	$T_{INT}+t_{i}$			
		3 days	7 days	14 days	21 days
25 mg kg^-1^	Effectiveness (%)	0.0	0.0	0.0	0.0
	Treatment times (days)	-–	-–	-–	-–
	Median volume (mm^3^)	16.3×10^3^	16.2×10^3^	15.7×10^3^	15.4×10^3^
	Volume range (mm^3^)	417.2 to 5.1×10^5^	532.7 to 5.0×10^5^	618.7 to 4.8×10^5^	682.9 to 4.7×10^5^
40 mg kg^-1^	Effectiveness (%)	0.0	0.0	0.0	0.0
	Treatment times (days)	-–	-–	-–	-–
	Median volume (mm^3^)	16.4×10^3^	17.3×10^3^	16.5×10^3^	16.0×10^3^
	Volume range (mm^3^)	413.9 to 4.5×10^5^	573.9 to 3.8×10^5^	562.7 to 4.5×10^5^	574.3 to 4.9×10^5^
70 mg kg^-1^	Effectiveness (%)	0.0	0.0	0.0	0.0
	Treatment times (days)	-–	-–	-–	-–
	Median volume (mm^3^)	15.3×10^3^	15.9×10^3^	16.8×10^3^	17.7×10^3^
	Volume range (mm^3^)	234.0 to 3.9×10^5^	410.3 to 4.6×10^5^	530.7 to 4.3×10^5^	566.4 to 4.0×10^5^

Effectiveness refers to the percentage of treatments that achieved a remission state; treatment times refer only to those treatments that reached a remission state. Volumetric metrics refer to all tumor dynamics defined by random combinations of model parameters.

CP, cyclophosphamide; SM, smaller tumors ( ${\text{V}}_{0}=200\;\text{m}{\text{m}}^{3};\;{\text{R}}_{0}=625\;\text{m}{\text{m}}^{3}$); LT, larger tumors ( ${\text{V}}_{0}=2,000\;\text{m}{\text{m}}^{3}$ and ${\text{R}}_{0}=6,250\;\text{m}{\text{m}}^{3}$).

### 3.3 Sensitivity analysis

#### 3.3.1 Sensitivity of drug administration to PF controller parameters

The Sobol indices are provided in **Table 11**. The following analyses will consider $parameter_{S1}$ as the first-order Sobol index of such parameter (example: $R_{S1}$ is the first-order index of parameter R) and $parameter_{ST}$ as the total-order Sobol index of such parameter (example: $R_{ST}$ is the total-order index of R).

**Table 11:** Sensitivity analyses using the Sobol indices to identify the contributions from each controller parameter to the average drug administration.

Parameter	Resistance-free chemotherapy				Therapy with chemoresistance			
	CP—SM		CP—LT		CP—SM		CP—LT	
	S1	ST	S1	ST	S1	ST	S1	ST
*R*	0.7325	0.7674	0.9796	0.9733	0.4773	0.5216	0.8904	0.9240
*G*	0.9839	0.9916	1.0000	1.0000	1.0000	1.0000	1.0000	1.0000
*M*_0_	0.8206	0.8352	0.8436	0.8400	0.4878	0.5233	0.9327	0.9492
*k*_0_	0.9687	0.9769	1.0107	1.0000	1.0000	1.0000	0.9136	0.9308
*k*_1_	0.4137	0.4182	0.1988	0.2066	1.0000	1.0000	0.2042	0.2755

S1, first-order Sobol indices; ST, total-order Sobol indices.

Concerning the results related to resistance-free chemotherapy and initial conditions $\;(V_{0},R_{0})=(200,\;625)$ ${\text{mm}}^{3}$: All parameters present non-negligible independent influence ( 0.4137≤S1≤0.9839), and the total contribution of each parameter is similar to the contribution of each parameter alone, i.e., their impact is mostly independent (no strong interactions were found: $R_{S1}\approx R_{ST};\;G_{S1}\approx G_{ST};\;M_{0-S1}\approx M_{0-ST}$; $k_{0-S1}\approx k_{0-ST};\;k_{1-S1}\approx k_{1-ST}$). Drug administration dynamics are mainly influenced by the parameters G ( $G_{S1}=0.9839$) and ( $k_{0-S1}=0.9687$), and the parameter $k_{1}$ is the one with the least influence $\left(k_{1-S1}=0.4137\ll G_{S1}\right)$. Concerning the results related to resistance-free chemotherapy and initial conditions $\left(V_{0},R_{0})=(2,000,6,250\right)$ ${\text{mm}}^{3}$, the following results are similar: (i) most parameters present non-negligible independent influence, namely, $R,G,M_{0}$ and $k_{0}$, (ii) the contribution of each parameter is mostly independent, (iii) parameters G ( $G_{S1}=1.0000$) and ( $k_{0-S1}=1.0000$) are the dominant parameters, and (iv) the least influence is provided by $k_{1}$ ( $k_{1-S1}=0.1988$). Differently, parameter R provides higher influence (increase from 0.7325 to 0.9796), and parameter $k_{1}$ presents a negligible independent influence ( $k_{1-S1}=0.1988$).

Concerning the results related to cancer therapies with chemoresistance and initial conditions $\;(N_{10},N_{20},\;R_{0})=(190,10,625)$ ${\text{mm}}^{3}$, not only *G* ( $G_{S1}=1.0000$) and $k_{0}$ ( $k_{0-S1}=1.0000$), but also $k_{1}$ ( $k_{1}=1.0000$). Indeed this significant influence occurs as $k_{1}=k_{m}t$, and chemoresistance usually increases with treatment duration. Their influence is mostly independent ( $G_{S1}\approx G_{ST};\;k_{0-S1}\approx k_{0-ST};\;k_{1-S1}\approx k_{1-ST}$). The least dominant parameter is $M_{0}\;(M_{0-S1}=0.4878$). Parameters *R* and $M_{0}$ participate in non-negligible interactions ( $R_{S1}=0.4773<R_{ST}=0.5216;\;M_{0-S1}=0.4878<M_{0-ST}=0.5233$). Concerning the results related to cancer therapies with chemoresistance and initial conditions $\;(N_{10},N_{20},\;R_{0})=(1,900,\;100,\;6,250)$ ${\text{mm}}^{3}$, drug administration dynamics are mainly influenced by parameter *G* ( $G_{S1}=1.0000$), even though significant influences are introduced by parameters *R*, $M_{0}$ and $k_{0}$ ( $R_{S1}=0.8904;\;M_{0-S1}=0.9327$; $k_{0-S1}=0.9136$). Surprisingly, the parameter with the least influence is $k_{1}$ ( $k_{1-S1}=0.2042$), even though the longest treatment times occurred under chemoresistance. Parameters $G,\;M_{0}$, and $k_{0}$ are mostly independent ( $G_{S1}=1.0000\approx G_{ST}=1.0000;\;M_{0-S1}=0.9327$ $\approx M_{0-ST}=0.9492;\;k_{0-S1}=0.9136\approx k_{0-ST}=0.9308$), but parameter $k_{1}\;$participates in non-negligible interactions ( $k_{1-S1}=0.2042<k_{1-ST}=0.2755$).

#### 3.3.2 Sensitivity of mechanobiological tumor dynamics to astrophysical dynamics

The influence of astrophysical dynamics on mechanobiological tumor dynamics was found to be mostly independent ( $parameter_{S1}\approx parameter_{ST}$), as shown in **Tables 12**, **13**. Concerning resistance-free chemotherapy (**Table 12**), the drag force inside protoplanets is usually the most dominant astrophysical phenomenon influencing mechanobiological dynamics, while accretion is usually the less dominant astrophysical phenomena:

**Table 12:** Sensitivity analyses using the Sobol indices for resistance-free chemotherapy to identify the contributions from each controller parameter on mechanobiological dynamics.

Parameter	Tumor growth				Cytotoxic effect of chemotherapy on the tumor volume			
	CP – SM		CP - LT		CP – SM		CP - LT	
	S1	ST	S1	ST	S1	ST	S1	ST
R	0.8993	0.9850	0.9855	0.9876	0.7106	0.7440	0.9571	0.9761
G	0.9979	1.0000	0.9938	0.9992	1.0000	1.0000	1.0000	0.9984
$M_{0}$	0.6140	0.6709	0.1992	0.2046	0.6600	0.6791	0.3657	0.3622
$k_{0}$	0.8405	0.8963	0.9935	0.9988	0.9401	0.9563	1.0000	0.9983
$k_{1}$	0.5024	0.5142	0.8150	0.8129	0.6595	0.6575	0.6622	0.6590
Parameter	Inhibition of vasculature by the tumor				Anti-angiogenic effect due to chemotherapy			
	CP – SM		CP - LT		CP – SM		CP - LT	
	S1	ST	S1	ST	S1	ST	S1	ST
R	0.9665	0.9461	0.9558	0.9698	0.7034	0.7328	0.9537	0.9592
G	1.0000	0.9931	0.9863	0.9956	0.9939	0.9964	1.0000	0.9969
$M_{0}$	1.0000	0.9855	0.9273	0.9152	0.7543	0.7693	0.6991	0.7106
$k_{0}$	1.0000	0.9800	0.9847	0.9955	0.9621	0.9731	1.0000	0.9971
$k_{1}$	0.0766	0.0728	0.1010	0.1039	0.5411	0.5454	0.3488	0.3375

S1, first-order Sobol indices; ST, total-order Sobol indices.

**Table 13:** Sensitivity analyses using the Sobol indices for therapy with chemoresistance to identify the contributions from each controller parameter on mechanobiological dynamics.

Parameter	Growth of the sensitive tumor tissue				Cytotoxic effect of chemotherapy on the sensitive tumor tissue			
	CP – SM		CP - LT		CP – SM		CP - LT	
	S1	ST	S1	ST	S1	ST	S1	ST
R	0.6820	0.7467	0.8774	0.8830	0.4868	0.5801	0.9376	0.9913
G	0.9857	1.0000	1.0000	0.9972	0.8698	0.9982	1.0000	1.0000
$M_{0}$	0.3043	0.3242	0.4113	0.4388	0.3789	0.5247	0.5091	0.5592
$k_{0}$	0.9551	0.9867	0.7017	0.7490	0.8585	0.9977	0.6598	0.7084
$k_{1}$	0.9857	1.0000	0.9846	0.9781	0.8698	0.9982	0.7734	0.7856
Parameter	Growth of the resistant tumor tissue				Stimulation of angiogenesis by sensitive and resistant tumor tissues			
	CP – SM		CP - LT		CP – SM		CP - LT	
	S1	ST	S1	ST	S1	ST	S1	ST
R	0.3111	0.5645	0.7883	0.8051	0.5888	0.7038	0.8365	0.8383
G	1.0000	1.0000	0.9132	0.9885	0.9764	1.0000	1.0000	0.9960
$M_{0}$	0.3528	0.6248	0.9042	0.9835	0.3816	0.3736	0.4253	0.4620
$k_{0}$	0.9971	1.0000	0.8640	0.9840	0.9532	0.9987	0.7265	0.7770
$k_{1}$	1.0000	1.0000	0.1709	0.2974	0.9763	1.0000	0.9556	0.9599
Parameter	Inhibition of vasculature by sensitive and resistant tumor tissues				Anti-angiogenic effect due to chemotherapy			
	CP – SM		CP - LT		CP – SM		CP - LT	
	S1	ST	S1	ST	S1	ST	S1	ST
R	0.5149	0.7850	0.7267	0.7715	0.5043	0.5366	0.8707	0.9203
G	1.0000	1.0000	0.9636	0.9804	0.9888	0.9936	1.0000	0.9978
$M_{0}$	0.1889	0.4880	0.5579	0.5854	0.4570	0.4924	0.8981	0.9013
$k_{0}$	1.0000	0.9867	0.7548	0.8576	0.9880	0.9927	0.8726	0.8867
$k_{1}$	1.0000	1.0000	0.7981	0.8270	0.9888	0.9936	0.3035	0.3670
Parameter	Mutation from resistant cells to sensitive cells				Mutation from sensitive cells to resistant cells			
	CP – SM		CP - LT		CP – SM		CP - LT	
	S1	ST	S1	ST	S1	ST	S1	ST
R	0.7529	0.5471	0.8591	0.9604	0.4764	0.5630	0.7088	0.8292
G	1.0000	0.9897	0.8616	0.9921	1.0000	1.0000	0.9142	1.0000
$M_{0}$	0.4250	0.4540	0.5867	0.6692	0.5534	0.6250	1.0000	1.0000
$k_{0}$	1.0000	0.9895	0.5979	0.8358	1.0000	1.0000	0.9481	1.0000
$k_{1}$	1.0000	0.9897	0.3769	0.6394	1.0000	1.0000	0.1448	0.2977

S1, first-order Sobol indices; ST, total-order Sobol indices.

(i) Tumor growth: It is mainly influenced by drag forces inside protoplanets both for small tumors ( $R_{S1},k_{0-S1}=(0.8993,\;0.8405\;$)) and large tumors ( $R_{S1},k_{0-S1}=(0.9855,\;0.9935$)). However, while accretion is the astrophysical dynamic that has the least influence on small tumors ( $k_{1-S1}=0.5024$), gravitation is the one with the least influence on large tumors ( $M_{0-S1}=0.1992$).
(ii) Cytotoxic effect of chemotherapy on tumor volume: It is also mainly influenced by drag forces inside protoplanets both on small tumors ( $R_{S1},k_{0-S1}=(0.7106,\;0.9401$)) and large tumors ( $R_{S1},k_{0-S1}=(0.9571,\;1.0000$)). This mechanobiological dynamic in small tumors is less influenced by gravitation ( $M_{0-S1}=0.6600$) and accretion ( $k_{1-S1}=0.6595$), even though gravitation is clearly the one with the least influence on large tumors ( $M_{0-S1}=0.3657$).
(iii) Inhibition of vasculature by the tumor: This mechanobiological dynamic is also mainly influenced by both drag forces inside protoplanets (small tumors: ( $R_{S1},k_{0-S1}=(0.9665,\;1.0000$)); large tumors: ( $R_{S1},k_{0-S1}=(0.9558,\;0.9847$))) and gravitation (small tumors: $M_{0-S1}=1.0000$; large tumors: $M_{0-S1}=0.9273)$. The influence of accretion is negligible (small tumors: $k_{1-S1}=0.0766$; large tumors: $k_{1-S1}=0.1010$).
(iv) Anti-angiogenic effect due to chemotherapy: It is mainly influenced by drag forces inside protoplanets (small tumors: ( $R_{S1},k_{0-S1}=(0.7034,\;0.9621$)); large tumors: ( $R_{S1},k_{0-S1}=(0.9537,\;1.0000$))) and less influenced by accretion (small tumors: $k_{1-S1}=0.5411$; large tumors: $k_{1-S1}=0.3488)$.

Concerning chemoresistant tumors (**Table 13**), accretion is usually the most dominant astrophysical phenomenon influencing mechanobiological dynamics, while gravitation is usually the less dominant astrophysical phenomenon (even though they have the least impact on specific initial tumor conditions):

(i) Growth of the sensitive tumor tissue: It is mainly influenced by the accretion (small tumors: $k_{1-S1}=0.9857$; large tumors: 0.9846), while gravitation is the astrophysical dynamics that has the least influence (small tumors: $M_{0-S1}=0.3043$; large tumors: $M_{0-S1}=0.4113$).
(ii) Cytotoxic effect of chemotherapy on sensitive tumor tissue: Small tumors are mainly influenced by accretion ( $k_{1-S1}=0.8698$), while both accretion ( $k_{1-S1}=0.7734$) and the drag force inside protoplanets ( $R_{S1},k_{0-S1}=(0.9376,\;0.6598$)) are the ones with a stronger influence on this mechanobiological dynamic. Gravitation is the one with the least influence on small tumors ( $M_{0-S1}=0.3789$) and large tumors ( $M_{0-S1}=0.5091$), even though its impact cannot be considered negligible.
(iii) Growth of the resistant tumor tissue: While accretion is the most dominant astrophysical phenomenon in small tumors ( $k_{1-S1}=1.0000$), it is the least dominant phenomenon in large tumors ( $k_{1-S1}=0.1709$). Differently, while gravitation is the least dominant astrophysical phenomenon in small tumors ( $M_{0-S1}=0.3528$), it is the most dominant phenomenon in large tumors ( $M_{0-S1}=0.9042$).
(iii) Stimulation of angiogenesis by sensitive and resistant tumor tissues: Similarly to the growth of sensitive tumor tissues, angiogenesis is mainly stimulated by accretion (small tumors: $k_{1-S1}=0.9763$; large tumors: $k_{1-S1}=\;$0.9556), while gravitation has the least influence on angiogenesis stimulation (small tumors: $M_{0-S1}=0.3816$; large tumors: $M_{0-S1}=0.4253$). Therefore, the growth of sensitive tumor tissue is highly dependent on angiogenesis stimulation.
(iv) Inhibition of vasculature by sensitive and resistant tumor tissues: It is also mainly influenced by accretion (small tumors: $k_{1-S1}=1.0000$; large tumors: 0.7981) and less influenced by gravitation (small tumors: $M_{0-S1}=0.1889$; large tumors: $M_{0-S1}=0.5579$). However, the impact of gravitation on the inhibition of vasculature is not negligible in large tumors ( $M_{0-S1}=0.5579$).
(v) Anti-angiogenic effect due to chemotherapy: Similarly to the growth of resistant tumor tissue, while accretion is the most dominant astrophysical phenomenon in small tumors ( $k_{1-S1}=0.9888$), it is the least dominant phenomenon in large tumors ( $k_{1-S1}=0.3035$). Differently, while gravitation is the least dominant astrophysical phenomenon in small tumors ( $M_{0-S1}=0.4570$; therefore, it is not a negligible influence), it is the most dominant phenomenon in large tumors ( $M_{0-S1}=0.8981$), even with strong influences ensured by drag forces inside protoplanets ( $R_{S1},k_{0-S1}=(0.8707,\;0.8867$)). Therefore, the growth of resistant tumor tissue is highly dependent on anti-angiogenic dynamics.
(vi) Mutation from resistant cells to sensitive cells: Accretion mainly influences this mechanobiological dynamics in small tumors ( $k_{1-S1}=1.0000$), but its influence is strongly reduced on large tumors ( $k_{1-S1}=0.3769$). The drag force inside protoplanets provides a strong influence on small tumors ( $R_{S1},k_{0-S1}=(0.7529,\;1.0000$)) but is the dominant phenomenon in large tumors ( $R_{S1},k_{0-S1}=(0.8591,\;0.5979$)).
(vii) Mutation from sensitive cells to resistant cells: Even though accretion provides the strongest influence on small tumors ( $k_{1-S1}=1.0000$) and the least one in large tumors ( $k_{1-S1}=0.1448$), as occurs in the mutation from resistant cells to sensitive cells, gravitation provides the least influence on small tumors (but not a negligible influence, as $M_{0-S1}=0.5534$) and the strongest influence on large tumors ( $M_{0-S1}=1.0000$), although strong influences ensured by drag forces inside protoplanets were found ( $R_{S1},k_{0-S1}=(0.7088,\;0.9481$)).

## 4 Discussion and conclusions

This study provides a promising control method to overcome the challenges associated with tumor heterogeneity and related highly uncertain tumor dynamics. Our results support the development of a new clinical therapeutic planning approach that goes far beyond empirical dosimetry and intermittent dosing. We demonstrated *in silico* that the use of PF-like dynamics ensures either tumor remission or retention states, regardless of the initial tumor volume, tumor growth dynamics, and potential impact of chemoresistance. Its robustness and adaptive behavior, associated with a continuous drug administration approach, revealed effective detumorization by non-evident highly non-linear drug administration, ensuring drug release independent of individual biological influences, as well as the uniformity of treatment time. Such a therapeutic approach will probably not require significant changes in clinical protocols, as the only change will be implemented in drug delivery systems. Instead of (current) therapeutic management based on manual programming, therapeutic management must be sophisticated to provide automatic drug dosing planning, according to a control law established by the PF controller, relying on the best therapeutic practices for patient safety. Through clinical translation investigations, the protocol for applying such a control method must be identified. Included is the identification of effective detumorization trajectories and controller parameters.

The survival probabilistic paradigm has produced clinical results that are far from exploring the full potential of dosage strategies. Indeed the 5-year survival rate of intermittent chemotherapy can reach 85% for breast cancer but can be lower than 10% for pancreatic cancer and glioblastoma (31, 64–66). Metronomic (continuous) chemotherapy has been mainly used as adjuvant or maintenance therapy; no large trials were conducted to compare the performance of metronomic and intermittent chemotherapies for curative purposes. Even though similar survival rates have been obtained for several cancer types (e.g., head and neck carcinoma), survival rates exceeding 40% were already reached for glioblastoma (67). Mathematical analyses using both deterministic and stochastic modeling also emphasize that metronomic adaptive dosing is able to provide significantly superior performances in comparison to intermittent therapy, mainly in reducing the drug concentration by 30%–80% and the resistant cell fixation by 40%–70% (1, 68–70). Our comparative analyses revealed that (i) considering chemotherapy-free treatments, the PF-inspired control required much lower average administration than intermittent schemes (small tumors: 1.26 vs. 20.75 mg kg^-1^ for effectiveness rates approximately 90%; large tumors: 1.39 mg kg^-1^ vs. 43.7 mg kg^-1^ to achieve approximately 99% effectiveness and (ii) considering cancer therapies with chemoresistance, intermittent treatments are not capable of detumorization dynamics ensuring remission states (and, therefore, they cannot be used as monotherapies); however, the PF-inspired administration is able to achieve very high effectiveness rates (87.3%), usually requiring lower average dosages than the ones that do not result in remission states through intermittent schemes. The use of the “time” component (integrating the accretion phenomenon) has proven to be a successful control parameter to deal with uncertain chemoresistance, which can pave the way to develop even more sophisticated controllers using additional dynamics found in planetary formation. Indeed we found that accretion is usually the most dominant astrophysical phenomenon influencing mechanobiological dynamics in cancer therapies with chemoresistance, while the drag force inside protoplanets is usually the most dominant one in resistance-free chemotherapy.

Various limitations must still be overcome, mainly those related to tumor models and controller design:

Our findings were reached using two mathematical models widely used to investigate tumor dynamics. However, these models do not consider multiscale cancer behaviors (atomic, molecular, microscopic, and macroscopic) (71, 72) as well as spatial dynamics for specific cancer types and vascularization, tumor–immune interactions during chemotherapy, and the synergistic effects due to the combination of therapies (such as chemo-immunotherapy) (73–76). Besides that, the primary modeled variables are formulated as phenomenological endpoints integrating multiple underlying biological effects. Other relevant dynamics must be considered, including hypoxic and multi-focal tumor lesions, immunosuppression, and glycosylation patterns (77, 78). As a computationally promising proof-of-concept study, we have methodologically chosen tumor models considering their realistic dynamics but as simple as possible (even though we used highly non-linear tumor dynamics governed of third-order and fourth-order); further research must integrate multiple hierarchies in cancer modeling. It is worth noting that modeling limitations were partially overcome as a wide range of parametric variations were considered.

Besides deterministic modeling, such as based on ordinary differential equations, stochastic modeling, such as based on agent-based modeling, must also be considered, such that the predictability of tumor dynamics, due to the random nature of mutations, can increase (32, 79). Stochasticity holds potential to provide a valuable prediction tool for drug-induced resistance (32).

Even though the PF-like controller revealed the ability to overcome both parametric and stochastic uncertainties, its performance in handling structural uncertainties must also be quantified in detail. Indeed performance limitations will emerge if uncertainties of multiscale models are not considered, which result in avoidable chemotoxicity. Besides that, uncertainties due to pharmacokinetic variability were not analyzed in this study; therefore, it is mandatory to analyze the performance of our new controller using different pharmacokinetics (e.g., by using atezolizumab).

Although PF control ensures high effectiveness and robustness to force detumorization dynamics, treatment times may be unnecessarily longer and/or dosages may be unnecessarily higher. This non-optimal behavior is due to the use of static parameters of tumor growth models (**Table 1**). Artificial intelligence (AI) algorithms can be used to minimize the secondary effects caused by this performance limitation. Big data related to tumor type and region, genetic profile, and comorbidities of patients, among others, can be used to adapt parameters of tumor growth models (e.g., on a daily basis), such that controller parameters can be refined/adapted throughout treatments.

Only four phenomena found in planetary formation were considered in the controller design (gravitation outside protoplanets, the density–mass relationship inside protoplanets, drag forces inside protoplanets, and accretion of protoplanets). Moreover, several assumptions were made, namely, considering protoplanets as uniform spheres with linear distribution of mass, the maximum protoplanet density as proportional to the minimum protoplanet density, linear drag forces, linear density–mass relationship, and the universal gravitation as realistically Newtonian. Planetary rearrangements by erosion and pebble accretion phenomena, as well as by mass–protoplanet collisions and resonance trapping, were not considered (30). As natural phenomena, they tend to adapt toward both prediction-error and energy minimization, holding potential to force highly effective therapeutic pathways toward tumor remission while ensuring minimal drug administration. Besides that, improved therapeutic outcomes could most likely be reached if Einsteinian time–space deformation theory is used to formulate the attraction between planetesimal bodies and protoplanets. Indeed the relativistic spacetime has natural curves, the geodesics, which ensures the shortest (known) natural paths between two regions (80). This phenomenon can then be used to minimize the treatment duration.

Even though we analyzed the influence of astrophysical-like dynamics on mechanobiological tumor dynamics, further investigations will be needed to explain several relevant effects, namely, the convergence of treatment times for different tumor volumes, the shorter treatment times obtained in larger chemoresistant tumors, and the (Bessel-like, diffusion-like, and logarithm-like) dosing patterns related to the response of PF controllers to uncertain tumor dynamics. Considering the highly nonlinear nature of tumor models and control law, it is very likely that analytical solutions can be found to mathematically explain these effects. A suitable approach to address this issue is most likely to use AI algorithms to find these correlative patterns.

The controller performance analysis was carried out considering all patients as eligible for this new anticancer treatment method. However, a screening process must be carefully implemented to identify which patients can undergo it. Firstly, it is mandatory to ensure that tumor removal is not urgent. Besides that, the eligibility criteria must rigorously consider critical tumor states, including the aggressive and metastatic ones, as they may require drug administrations that exceed the maximum safe dose limits, which can be further aggravated by comorbidities.

Our results represent a proof-of-concept based on computer simulations. The full potential of this new approach requires that the performance of our PF controller must be analyzed *in vivo* and compared with using multiscale models. These include (i) *in silico* 3D multi-physical spatiotemporal models defining tumor microregions (cell clusters separated by stomal components) and spatial subclones (microregions with shared genetic alterations) (19) and (ii) patient-derived organoids (PDOs) and PDO-derived xenografts (PDOXs) in animal models. Drug administration must be conducted according to various administration methods, including those consistent with standard clinical routes for cyclophosphamide (e.g., including intravenous, intramuscular, and intraperitoneal), such that the best administration method can be found for geometrodynamics performed by PF control. Micro-electromechanical systems (MEMS) can be designed as drug delivery devices, such that the classical drug administration during hospitalization can be extended to at-home or hospital-at-home drug administration. New studies must be conducted to test the robustness of PF controllers for a wide range of tumor volumes (from micro-size to massive size) as well as to evaluate whether reducing/debulking tumor masses enhance the effectiveness of chemotherapy controlled by geometrodynamics defined by PF controllers. Finally, AI algorithms must be developed both to optimize the controller’s parameters and to analyze correlation patterns between astrophysical dynamics and mechanobiological dynamics that cannot be mathematically resolved.

## Data Availability

The original contributions presented in the study are included in the article/**Supplementary Material**. Further inquiries can be directed to the corresponding author. The source code (Matlab and Simulink) developed for this work is available at the DUnAs repository (University of Aveiro): https://doi.org/10.48527/TEIF8G.
