# Supplementary material for: Continuous adaptive chemotherapy dosing by using planet-formation-inspired dynamics: analytical and in silico insights
Source: Front Oncol. 2026 May 11;16:1761791. doi: 10.3389/fonc.2026.1761791 (PMC13200006; doi:10.3389/fonc.2026.1761791)
Supplement: Supplementary file 1 [file SupplementaryFile1.docx]

Supplementary information - Additional figures and mathematical demonstrations

Continuous adaptive chemotherapy dosing by using Planet Formation-inspired dynamics: Analytical and in-silico insights

Marco P. Soares dos Santos^1,2*^, Sérgio X. F. Santos^1^, Rodrigo M. C. Bernardo^1^, João V. Vidal^3,4^, Gil Gonçalves^1,2^

^1^ Center for Mechanical Technology & Automation (TEMA), Department of Mechanical Engineering, University of Aveiro, Aveiro, Portugal.

^2^ Intelligent Systems Associate Laboratory (LASI), Guimarães, Portugal.

^3^ Department of Physics and Aveiro Institute of Materials (CICECO), University of Aveiro, Aveiro, Portugal.

^4^ Department of Physics and Institute for Nanostructures, Nanomodelling and Nanofabrication (I3N), University of Aveiro, Aveiro, Portugal.

***1. Additional Figures related to the influence of biological parameters on the performance of Planet Formation control***

**1.1 Results for resistance-free chemotherapy**

| 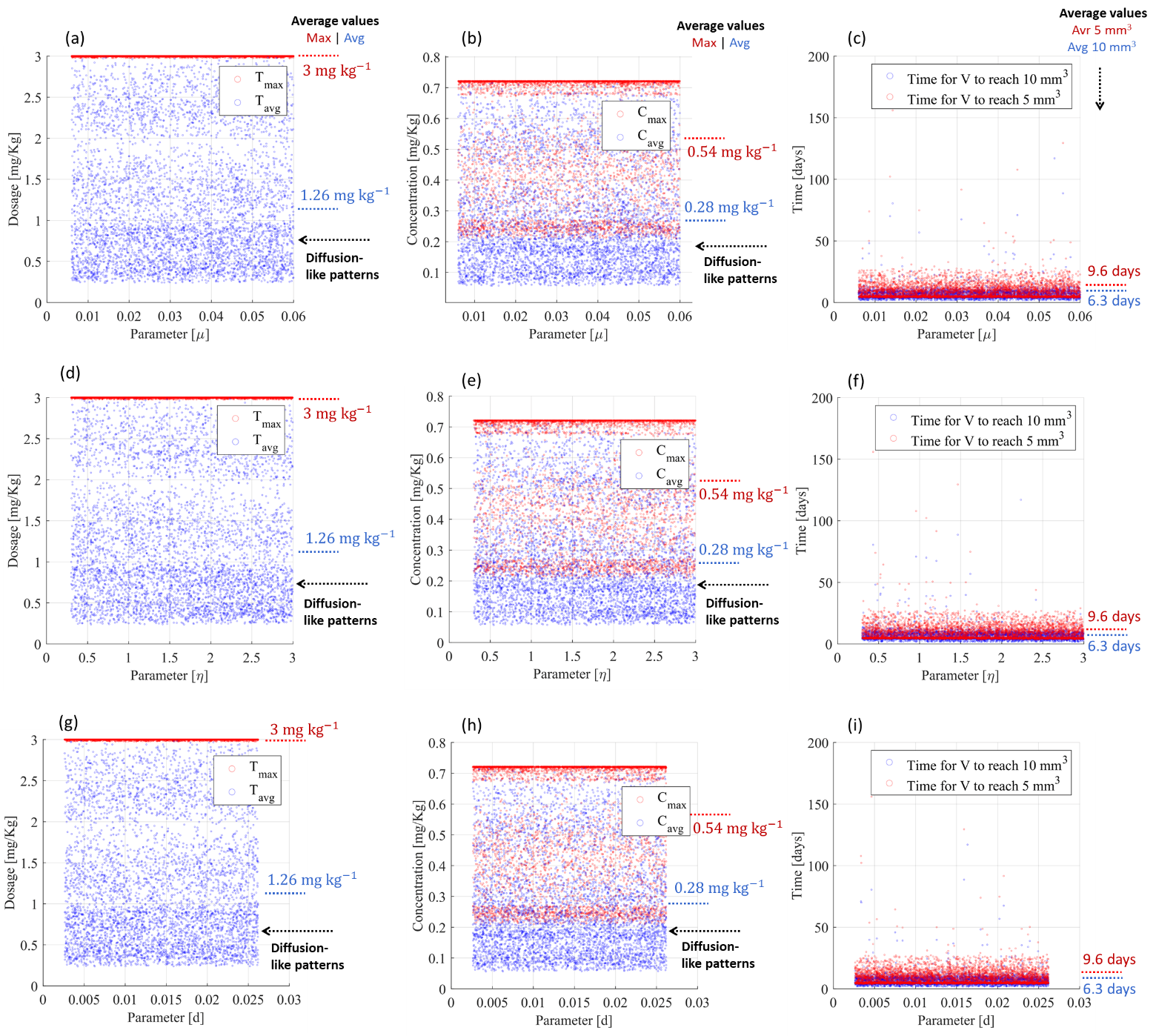 |
| --- |
| **Fig. S1** \| Resistance-free chemotherapy using Cyclophosphamide for the smaller tumoral scenarios. Influence of the loss of endothelial support ($\mu$) on **(a)** dosing administration, **(b)** drug concentration, and **(c)** treatment time. Influence of cytotoxic effect on the vasculature ($\eta$) on **(d)** dosing administration, **(e)** drug concentration, and **(f)** treatment time. Influence of the angiogenic inhibition ($d$) on **(g)** dosing administration, **(h)** drug concentration, and **(i)** treatment time. |

| 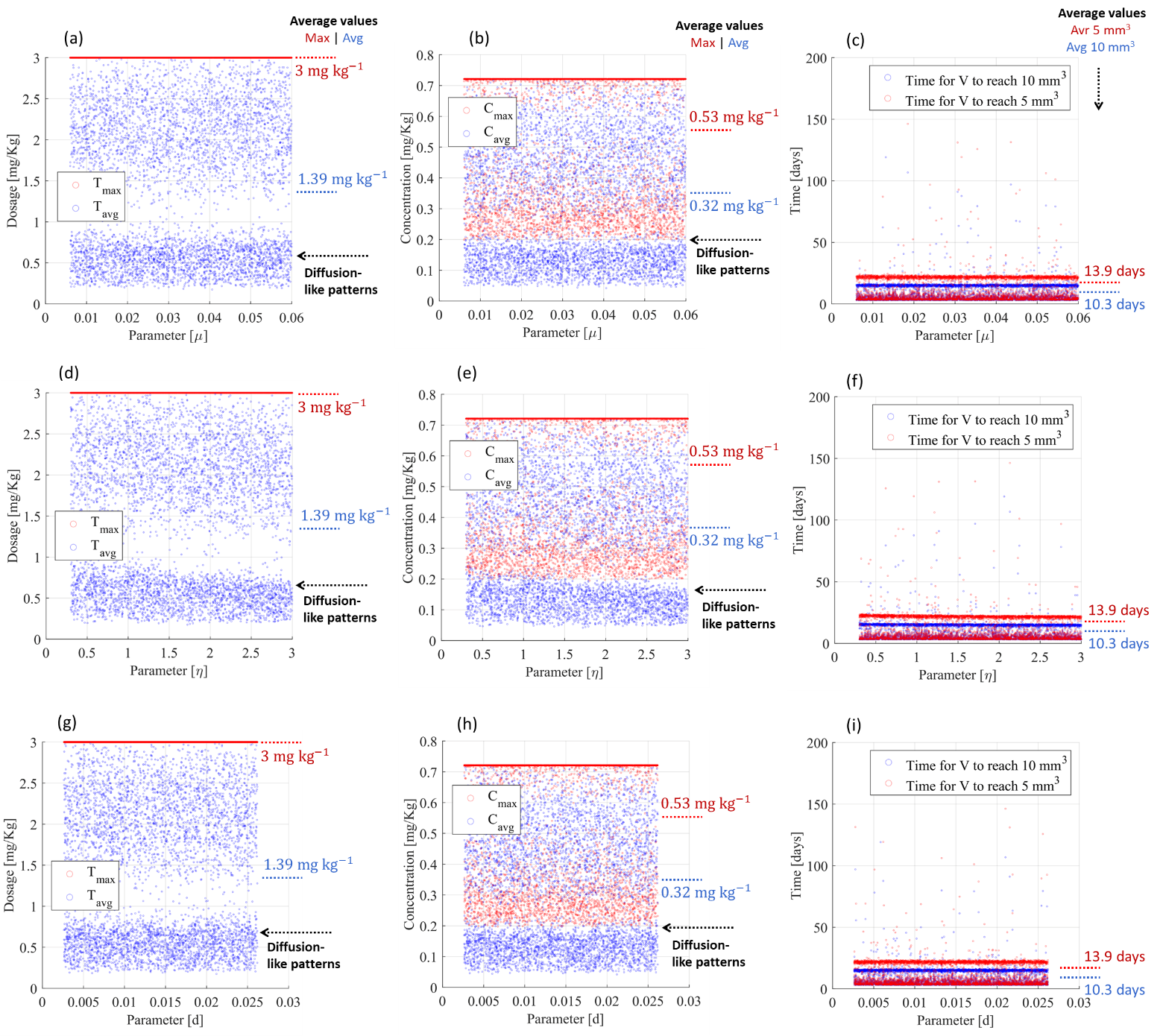 |
| --- |
| **Fig. S2** \| Resistance-free chemotherapy using Cyclophosphamide for the larger tumoral scenarios. Influence of the loss of endothelial support ($\mu$) on **(a)** dosing administration, **(b)** drug concentration, and **(c)** treatment time. Influence of cytotoxic effect on the vasculature ($\eta$) on **(d)** dosing administration, **(e)** drug concentration, and **(f)** treatment time. Influence of the angiogenic inhibition ($d$) on **(g)** dosing administration, **(h)** drug concentration, and **(i)** treatment time. |

**1.2 Results for cancer therapy with chemoresistance**

| 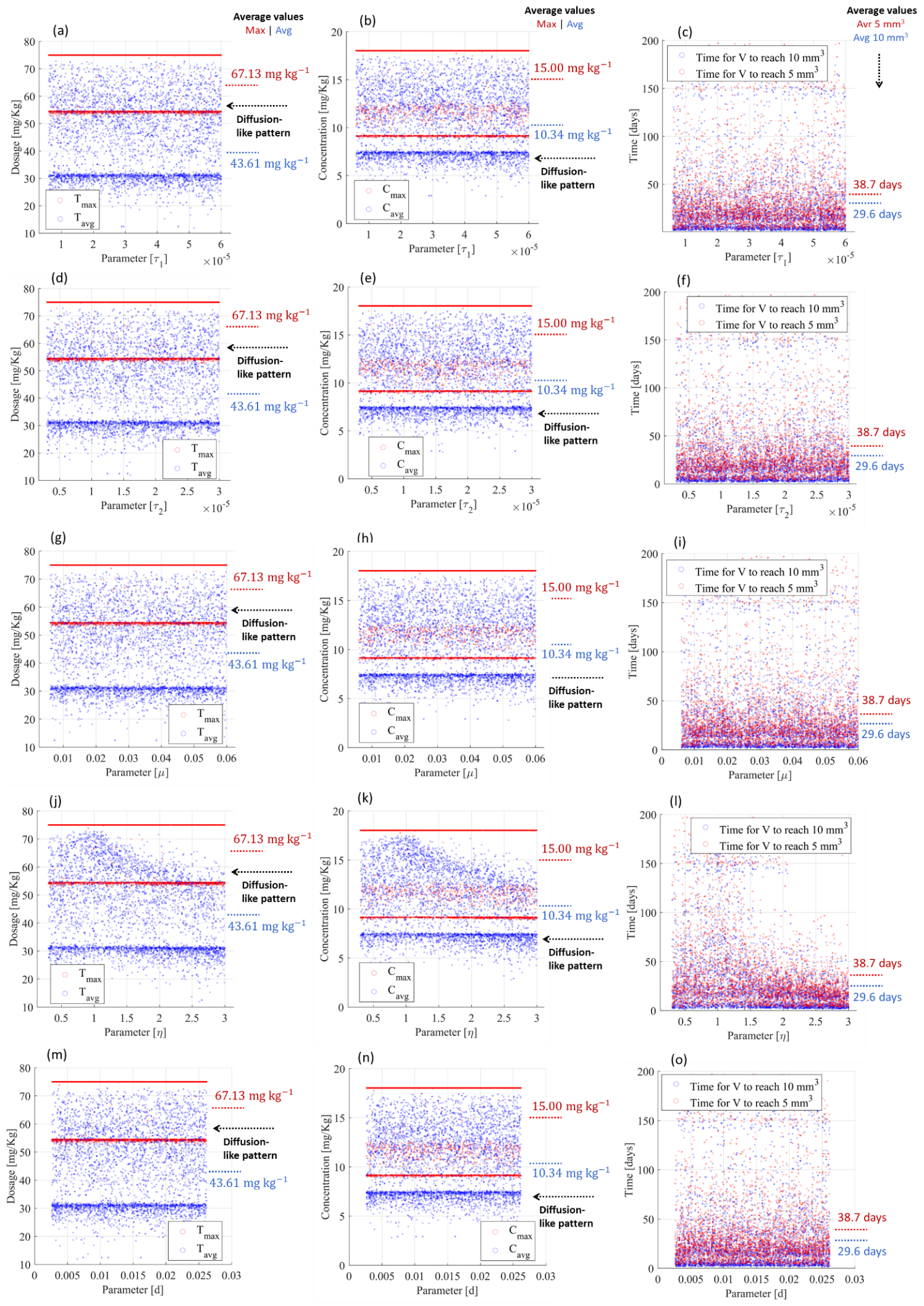 |
| --- |
| **Fig. S3** \| Cancer therapy with chemoresistance using Cyclophosphamide for the smaller tumoral scenarios. Influence of the cellular mutation rate ($\tau_{1}$) on **(a)** dosing administration, **(b)** drug concentration, and **(c)** treatment time. Influence of the cellular reverse mutation rate ($\tau_{2}$) on **(d)** dosing administration, **(e)** drug concentration, and **(f)** treatment time. Influence of the loss of endothelial support ($\mu$) on **(g)** dosing administration, **(h)** drug concentration, and **(i)** treatment time. Influence of cytotoxic effect on the vasculature ($\eta$) on **(j)** dosing administration, **(k)** drug concentration, and **(l)** treatment time. Influence of the angiogenic inhibition ($d$) on **(m)** dosing administration, **(n)** drug concentration, and **(o)** treatment time. |
| 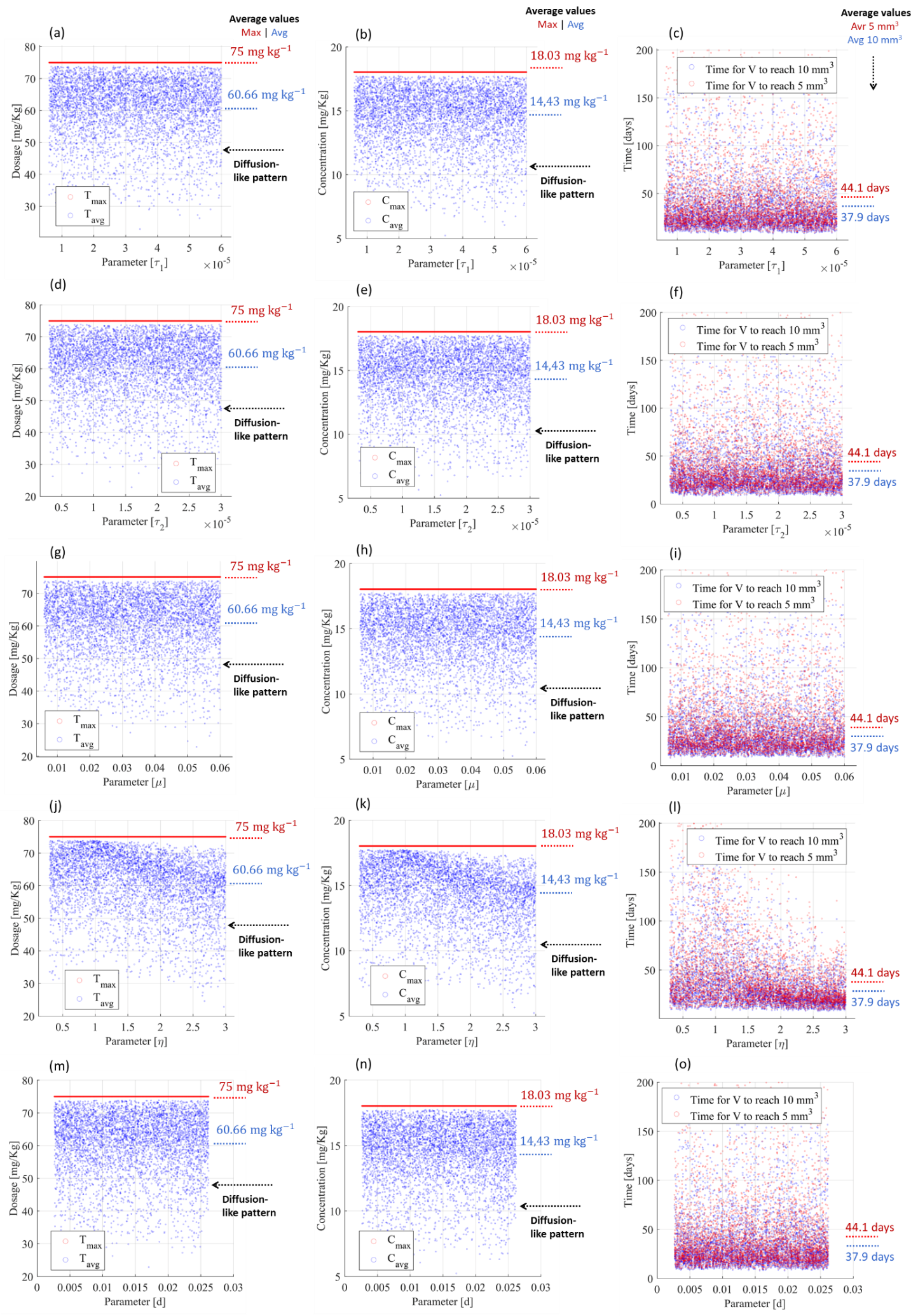 |
| **Fig. S4** \| (Cancer therapy with chemoresistance using Cyclophosphamide for the larger tumoral scenarios. Influence of the cellular mutation rate ($\tau_{1}$) on **(a)** dosing administration, **(b)** drug concentration, and **(c)** treatment time. Influence of the cellular reverse mutation rate ($\tau_{2}$) on **(d)** dosing administration, **(e)** drug concentration, and **(f)** treatment time. Influence of the loss of endothelial support ($\mu$) on **(g)** dosing administration, **(h)** drug concentration, and **(i)** treatment time. Influence of cytotoxic effect on the vasculature ($\eta$) on **(j)** dosing administration, **(k)** drug concentration, and **(l)** treatment time. Influence of the angiogenic inhibition ($d$) on **(m)** dosing administration, **(n)** drug concentration, and **(o)** treatment time. |

***2. Mathematical demonstrations related to resistance-free chemotherapy***

### 2.1 Model Linearization

The tumor growth model related to resistance-free chemotherapy was linearized, as follows:

| $\dot{V}_{Lt}=\Lambda_{t1}+\nabla_{VV}\left( V_{Lt}-V_{t} \right)+\nabla_{VR}\left( R_{Lt}-R_{t} \right),$ | (1) |
| --- | --- |
| $\dot{R}_{Lt}=\Lambda_{t2}+\nabla_{RV}\left( V_{Lt}-V_{t} \right)+\nabla_{RR}\left( R_{Lt}-R_{t} \right),$ | (2) |
| $\dot{C}_{Lt}=\dot{C}_{t}=-\lambda C_{t}+T_{t},$ | (3) |

where $V_{Lt}$, $R_{Lt}, C_{t}, T_{t}$ are the tumor volume, the vasculature capacity, the drug concentration and the drug administration, respectively, at instant time $t$,

$\Lambda_{t1}=-\xi\ln\left( \frac{V_{t}}{R_{t}} \right)V_{t}-\varphi V_{t}C_{t};$ $\Lambda_{t2}=bV_{t}-\left( \mu+d{V_{t}}^{\frac{2}{3}} \right)R_{t}-\eta R_{t}C_{t};$

$$\nabla_{VV}=-\xi\left( V_{t}\frac{1}{\frac{V_{t}}{R_{t}}}\frac{1}{R_{t}}+\ln\left( \frac{V_{t}}{R_{t}} \right) \right)-\varphi C_{t}=-\xi\left( 1+\ln\left( \frac{V_{t}}{R_{t}} \right) \right)-\varphi C_{t}; \nabla_{VR}=-\xi V_{t}\left( \frac{1}{\frac{V_{t}}{R_{t}}}\left( -\frac{V_{t}}{R_{t}^{2}} \right) \right)=\frac{\xi V_{t}}{R_{t}} ;$$

$$\nabla_{RV}=b-\frac{2}{3}{dR}_{t}{V_{t}}^{-\frac{1}{3}} ; \nabla_{RR}=\mu+d{V_{t}}^{\frac{2}{3}}-\eta C_{t}.$$

Taking

$$\nabla_{VV-C}=-\xi\left( 1+\ln\left( \frac{V_{t}}{R_{t}} \right) \right),$$

and

$$\nabla_{RR-C}=\mu+d{V_{t}}^{\frac{2}{3}} ,$$

and rewriting Eqs. (1) and (2), then

| $\dot{V}_{Lt}=\Lambda_{t1}+\nabla_{VV-C}\left( V_{Lt}-V_{t} \right)+\nabla_{VR}\left( R_{Lt}-R_{t} \right)-\varphi\left( V_{Lt}-V_{t} \right)C_{t},$ | (4) |
| --- | --- |
| $\dot{R}_{Lt}=\Lambda_{t2}+\nabla_{RV}\left( V_{Lt}-V_{t} \right)+\nabla_{RR-C}\left( R_{Lt}-R_{t} \right)-\eta\left( R_{Lt}-R_{t} \right)C_{t},$ | (5) |
| $\dot{C}_{Lt}=\dot{C}_{t}=-\lambda C_{t}+T_{t},$ | (6) |

with

$$\Lambda_{t1}=-\xi\ln\left( \frac{V_{t}}{R_{t}} \right)V_{t}-\varphi V_{t}C_{t};\Lambda_{t2}=bV_{t}-\left( \mu+d{V_{t}}^{\frac{2}{3}} \right)R_{t}-\eta R_{t}C_{t}; \nabla_{VV-C}=-\xi\left( 1+\ln\left( \frac{V_{t}}{R_{t}} \right) \right);$$

$$\nabla_{RR-C}=\mu+d{V_{t}}^{\frac{2}{3}}; \nabla_{VR}=\frac{\xi V_{t}}{R_{t}};\nabla_{RV}=b\left( 1-\frac{2{dR}_{t}}{3b\sqrt[3]{V_{t}}} \right).$$

### 2.2 Closed-loop system for $\boldsymbol{r>R}$

Taking $z_{A}=\int_{0}^{t} r dt=\int_{0}^{t} (V_{Lt-A}-u+\delta)dt$, where the subscript $A$ refers to the region where $r>R$**,** the PF dosing controller can be incorporated into a closed-loop system $\boldsymbol{f}_{\boldsymbol{t-A}}$ defined as (Figure S5A):

| ${\dot{\mathbf{x}}}_{\boldsymbol{t-A}}\mathbf{=}f_{t-A}\left( t\mathbf{,}\mathbf{x}_{\boldsymbol{t-A}} \right)\mathbf{=}\left[ \begin{matrix} f_{t1-A}\left( t,\mathbf{x}_{\boldsymbol{t-A}} \right) \\ f_{t2-A}\left( t,\mathbf{x}_{\boldsymbol{t-A}} \right) \\ f_{t3-A}\left( t,\mathbf{x}_{\boldsymbol{t-A}} \right) \\ f_{t4-A}\left( t,\mathbf{x}_{\boldsymbol{t-A}} \right) \end{matrix} \right]$  $\mathbf{=}\left\{ \begin{matrix} \dot{V}_{Lt-A}=\Lambda_{t1}+\nabla_{VV-C}\left( V_{Lt-A}-V_{t} \right)+\nabla_{VR}\left( R_{Lt-A}-R_{t} \right)-\varphi\left( V_{Lt-A}-V_{t} \right)C_{PL-t-A}, \\ \dot{R}_{Lt-A}=\Lambda_{t2}+\nabla_{RV}\left( V_{Lt-A}-V_{t} \right)+\nabla_{RR-C}\left( R_{Lt-A}-R_{t} \right)-\eta\left( R_{Lt-A}-R_{t} \right)C_{PL-t-A}, \\ \dot{C}_{PL-t-A}=-\lambda C_{PL-t-A}+\frac{G\left( M_{0}+e^{k_{1}z_{A}^{2}} \right)}{\left( V_{Lt-A}-u+2\delta\right)^{2}}, \\ \dot{z}_{A}=V_{Lt-A}-u+\delta. \end{matrix} \right.$ | (7) |  |
| --- | --- | --- |
| 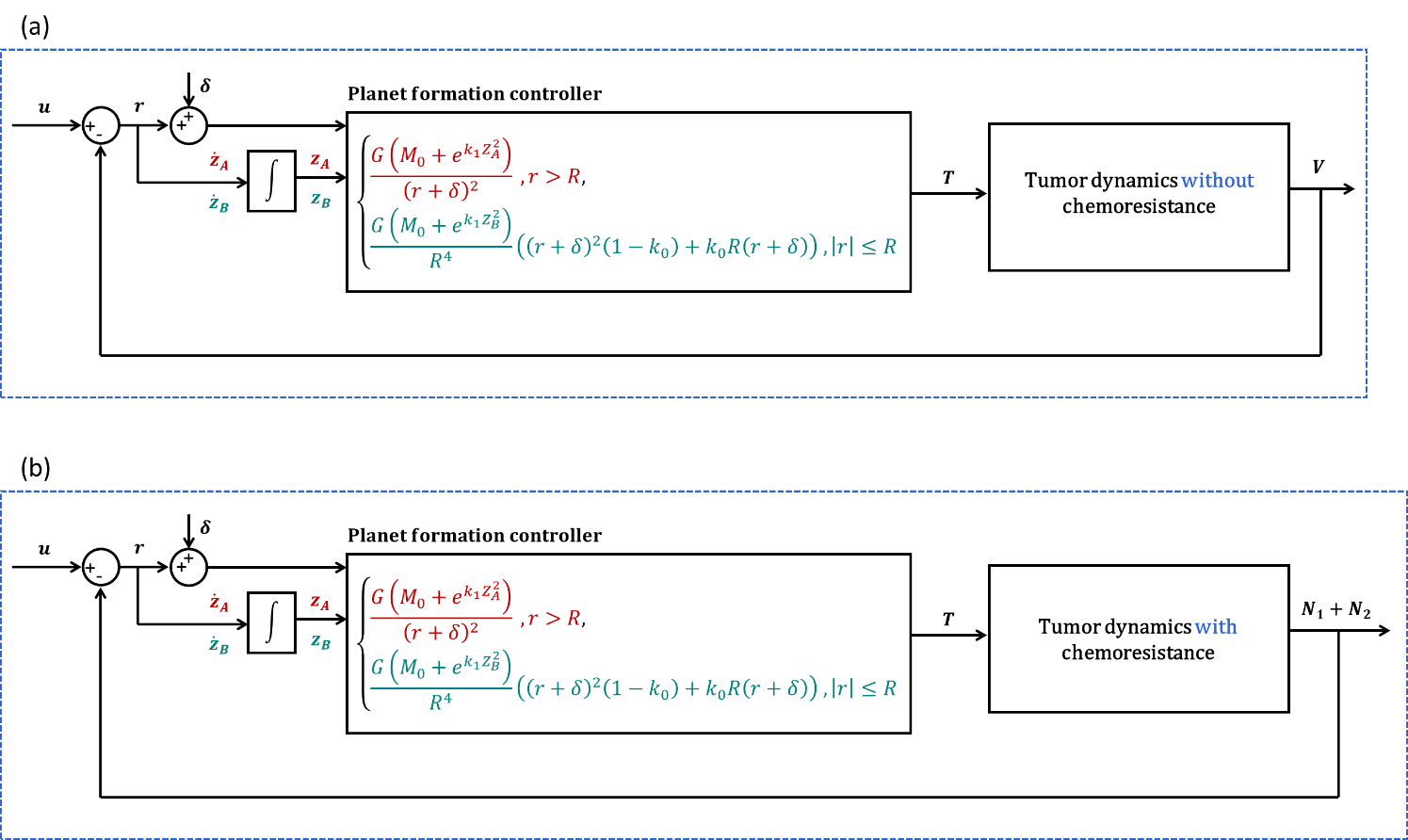 | | |
| Fig. S5 \| Closed-loop system incorporating the PF controller for detumorization dynamics (a) without chemoresistance; (b) with chemoresistance. | | |

### 2.3 Closed-loop system for $\boldsymbol{|r|\leq R}$

Taking $z_{B}=\int_{0}^{t} r dt=\int_{0}^{t} (V_{Lt-B}-u+\delta)dt$, where the subscript $B$ refers to the region where $|r|\leq R$, the PF dosing controller can be incorporated into a closed-loop system $\boldsymbol{f}_{\boldsymbol{t-B}}$ defined as (Figure S5A):

| ${\dot{\mathbf{x}}}_{\boldsymbol{t-B}}\mathbf{=}f_{t-B}\left( t\mathbf{,}\mathbf{x}_{\boldsymbol{t-B}} \right)\mathbf{=}\left[ \begin{matrix} f_{t1-B}\left( t,\mathbf{x}_{\boldsymbol{t-B}} \right) \\ f_{t2-B}\left( t,\mathbf{x}_{\boldsymbol{t-B}} \right) \\ f_{t3-B}\left( t,\mathbf{x}_{\boldsymbol{t-B}} \right) \\ f_{t4-B}\left( t,\mathbf{x}_{\boldsymbol{t-B}} \right) \end{matrix} \right]$  $\boldsymbol{=}\left\{ \begin{matrix} \dot{V}_{Lt-B}=\Lambda_{t1}+\nabla_{VV-C}\left( V_{Lt-B}-V_{t} \right)+\nabla_{VR}\left( R_{Lt-B}-R_{t} \right)-\varphi\left( V_{Lt-B}-V_{t} \right)C_{PL-t-B}, \\ \dot{R}_{Lt-B}=\Lambda_{t2}+\nabla_{RV}\left( V_{Lt-B}-V_{t} \right)+\nabla_{RR-C}\left( R_{Lt-B}-R_{t} \right)-\eta\left( R_{Lt-B}-R_{t} \right)C_{PL-t-B}, \\ \dot{C}_{PL-t-B}=-\lambda C_{PL-t-B}+T_{PL-t-B}, \\ \dot{z}_{B}=V_{Lt-B}-u+\delta, \end{matrix} \right.$  $\mathbf{=}\left\{ \begin{matrix} \dot{V}_{Lt-B}=\Lambda_{t1}+\nabla_{VV-C}\left( V_{Lt-B}-V_{t} \right)+\nabla_{VR}\left( R_{Lt-B}-R_{t} \right)-\varphi\left( V_{Lt-B}-V_{t} \right)C_{PL-t-B}, \\ \dot{R}_{Lt-C}=\Lambda_{t2}+\nabla_{RV}\left( V_{Lt-B}-V_{t} \right)+\nabla_{RR-C}\left( R_{Lt-B}-R_{t} \right)-\eta\left( R_{Lt-B}-R_{t} \right)C_{PL-t-B}, \\ \dot{C}_{PL-t-B}=-\lambda C_{PL-t-B}+\frac{G\left( M_{0}+e^{k_{1}z_{B}^{2}} \right)}{R^{4}} \left( ({V_{Lt-B}-u+2\delta)}^{2}(1-k_{0})+k_{0}R(V_{Lt-B}-u+2\delta) \right), \\ \dot{z}_{B}=V_{Lt-B}-u+\delta. \end{matrix} \right.$ | (8) |
| --- | --- |

### 2.4 Existence and uniqueness of solutions provided by the PF controller

*Closed-loop system* $f_{t-A}\left( t,x_{t-A} \right)$*:*

$$J_{f_{t}-A}=\left[ \begin{matrix} \frac{\partial f_{t1-A}}{\partial V_{Lt-A}} & \frac{\partial f_{t1-A}}{\partial R_{Lt-A}} & \frac{\partial f_{t1-A}}{\partial C_{PL-t-A}} & \frac{\partial f_{t1-A}}{\partial z_{A}} \\ \frac{\partial f_{t2-A}}{\partial V_{Lt-A}} & \frac{\partial f_{t2-A}}{\partial R_{Lt-A}} & \frac{\partial f_{t2-A}}{\partial C_{PL-t-A}} & \frac{\partial f_{t2-A}}{\partial z_{A}} \\ \frac{\partial f_{t3-A}}{\partial V_{Lt-A}} & \frac{\partial f_{t3-A}}{\partial R_{Lt-A}} & \frac{\partial f_{t3-A}}{\partial C_{PL-t-A}} & \frac{\partial f_{t3-A}}{\partial z_{A}} \\ \frac{\partial f_{t4-A}}{\partial V_{Lt-A}} & \frac{\partial f_{t4-A}}{\partial R_{Lt-A}} & \frac{\partial f_{t4-A}}{\partial C_{PL-t-A}} & \frac{\partial f_{t4-A}}{\partial z_{A}} \end{matrix} \right]=\left[ \begin{matrix} \frac{\partial f_{t1-A}}{\partial V_{Lt-A}} & \nabla_{VR} & \frac{\partial f_{t1-A}}{\partial C_{PL-t-A}} & 0 \\ \nabla_{RV} & \frac{\partial f_{t2-A}}{\partial R_{Lt-A}} & \frac{\partial f_{t2-A}}{\partial C_{PL-t-A}} & 0 \\ \frac{\partial f_{t3-A}}{\partial V_{Lt-A}} & 0 & -\lambda& \frac{\partial f_{t3-A}}{\partial z_{A}} \\ 1 & 0 & 0 & 0 \end{matrix} \right].$$

Concerning $f_{t1-A}(t,\boldsymbol{x}_{\boldsymbol{t-A}})$***:***

$$\frac{\partial f_{t1-A}}{\partial V_{Lt-A}}=\nabla_{VV-C}\boldsymbol{-}\varphi C_{PL-t-A}; \frac{\partial f_{t1-A}}{\partial R_{Lt-A}}=\nabla_{VR}; \frac{\partial f_{t1-A}}{\partial C_{PL-t-A}}=-\varphi\left( V_{Lt-A}-V_{t} \right); \frac{\partial f_{t1-A}}{\partial z_{A}}=0.$$

Concerning $f_{t2-A}(t,\boldsymbol{x}_{\boldsymbol{t-A}})$***:***

$$\frac{\partial f_{t2-A}}{\partial V_{Lt-A}}=\nabla_{RV}; \frac{\partial f_{t2-A}}{\partial R_{Lt-A}}=\nabla_{RR-C}-\eta C_{PL-t-A}; \frac{\partial f_{t2-A}}{\partial C_{PL-t-A}}=-\eta\left( R_{Lt-A}-R_{t} \right); \frac{\partial f_{t2-A}}{\partial z_{A}}=0.$$

Concerning $f_{t3-A}(t,\boldsymbol{x}_{\boldsymbol{t-A}})$***:***

$$\frac{\partial f_{t3-A}}{\partial V_{Lt-A}}=\frac{2G\left( M_{0}+e^{k_{1}z_{A}^{2}} \right)}{\left( V_{Lt-A}-u+2\delta\right)^{3}}; \frac{\partial f_{t3-A}}{\partial R_{Lt-A}}=0; \frac{\partial f_{t3-A}}{\partial C_{PL-t-A}}=-\lambda; \frac{\partial f_{t3-A}}{\partial z_{A}}=\frac{2Gk_{1}z_{A} e^{k_{1}z_{A}^{2}}}{\left( V_{Lt-A}-u+2\delta\right)^{2}}\boldsymbol{.}$$

Concerning $f_{t4-A}(t,\boldsymbol{x}_{\boldsymbol{t-A}})$***:***

$$\frac{\partial f_{t4-A}}{\partial V_{Lt-A}}=1; \frac{\partial f_{t4-A}}{\partial R_{Lt-A}}=0; \frac{\partial f_{t4-A}}{\partial C_{PL-t-A}}=0; \frac{\partial f_{t4-A}}{\partial z_{A}}=0$$

All partial derivatives of $f_{t-A}\left( t\mathbf{,}\mathbf{x}_{\boldsymbol{t-A}} \right)$ are continuous and bounded $\forall t \in[t_{0},t_{1}]$. Notice that: $\frac{\partial f_{t3-A}}{\partial V_{Lt-A}}$ and $\frac{\partial f_{t3-A}}{\partial z_{A}}$ contain state-dependent denominators, but such denominators are nonzero even if $\left( V_{Lt-A}-u \right)\to0$, as $\delta$ establishes a boundary; exponential terms included in $\frac{\partial f_{t3-A}}{\partial V_{Lt-A}}$ and $\frac{\partial f_{t3-A}}{\partial z_{A}}$ are bounded, as $z_{A}$ is finite for remission states $\left( V_{Lt-A}-u \right)\to0$. Therefore, $f_{t-A}\left( t\mathbf{,}\mathbf{x}_{\boldsymbol{t-A}} \right)$ is globally Lipschitz $\forall t\in[t_{0},t_{1}]$. As $f_{t-A}\left( t\mathbf{,}\mathbf{x}_{\boldsymbol{t-A}} \right)$ is continuous, then they are also piece-wise continuous. Since$f_{t-A}\left( t\mathbf{,}\mathbf{x}_{\boldsymbol{t-A}} \right)$ is globally Lipschitz and piece-wise continuous, then $\mathbf{x}_{\boldsymbol{t-A}}\mathbf{=}f_{t-A}\left( t\mathbf{,}\mathbf{x}_{\boldsymbol{t-A}} \right)$ has a unique solution $\forall t\in[t_{0},t_{1}]$ for each set of initial conditions $\mathbf{x}_{\boldsymbol{0}\boldsymbol{t-A}}$and for each trajectory $u$.

*Closed-loop system* $f_{t-B}\left( t,x_{t-B} \right):$

$$J_{f_{t}-B}=\left[ \begin{matrix} \frac{\partial f_{t1-B}}{\partial V_{Lt-B}} & \frac{\partial f_{t1-B}}{\partial R_{Lt-B}} & \frac{\partial f_{t1-B}}{\partial C_{BH-t-B}} & \frac{\partial f_{t1-B}}{\partial z_{B}} \\ \frac{\partial f_{t2-B}}{\partial V_{Lt-B}} & \frac{\partial f_{t2-B}}{\partial R_{Lt-B}} & \frac{\partial f_{t2-B}}{\partial C_{BH-t-B}} & \frac{\partial f_{t2-B}}{\partial z_{B}} \\ \frac{\partial f_{t3-B}}{\partial V_{Lt-B}} & \frac{\partial f_{t3-B}}{\partial R_{Lt-B}} & \frac{\partial f_{t3-B}}{\partial C_{BH-t-B}} & \frac{\partial f_{t3-B}}{\partial z_{B}} \\ \frac{\partial f_{t4-B}}{\partial V_{Lt-B}} & \frac{\partial f_{t4-B}}{\partial R_{Lt-B}} & \frac{\partial f_{t4-B}}{\partial C_{BH-t-B}} & \frac{\partial f_{t4-B}}{\partial z_{B}} \end{matrix} \right]=\left[ \begin{matrix} \frac{\partial f_{t1-B}}{\partial V_{Lt-B}} & \nabla_{VR} & \frac{\partial f_{t1-B}}{\partial C_{PL-t-B}} & 0 \\ \nabla_{RV} & \frac{\partial f_{t2-B}}{\partial R_{Lt-B}} & \frac{\partial f_{t2-B}}{\partial C_{PL-t-B}} & 0 \\ \frac{\partial f_{t3-B}}{\partial V_{Lt-B}} & 0 & -\lambda& \frac{\partial f_{t3-B}}{\partial z_{B}} \\ 1 & 0 & 0 & 0 \end{matrix} \right].$$

Concerning $f_{t1-B}(t,\boldsymbol{x}_{\boldsymbol{t-B}})$***:***

$$\frac{\partial f_{t1-B}}{\partial V_{Lt-B}}=\nabla_{VV-B}\boldsymbol{-}\varphi C_{PL-t-B}; \frac{\partial f_{t1-B}}{\partial R_{Lt-B}}=\nabla_{VR}; \frac{\partial f_{t1-B}}{\partial C_{PL-t-B}}=-\varphi\left( V_{Lt-B}-V_{t} \right); \frac{\partial f_{t1-B}}{\partial z_{B}}=0.$$

Concerning $f_{t2-B}(t,\boldsymbol{x}_{\boldsymbol{t-B}})$***:***

$$\frac{\partial f_{t2-B}}{\partial V_{Lt-B}}=\nabla_{RV}; \frac{\partial f_{t2-B}}{\partial R_{Lt-B}}=\nabla_{RR-C}-\eta C_{PL-t-B}; \frac{\partial f_{t2-B}}{\partial C_{PL-t-B}}=-\eta\left( R_{Lt-B}-R_{t} \right); \frac{\partial f_{t2-B}}{\partial z_{B}}=0.$$

Concerning $f_{t3-B}(t,\boldsymbol{x}_{\boldsymbol{t-B}})$***:***

$$\frac{\partial f_{t3-B}}{\partial V_{Lt-B}}=\frac{G\left( M_{0}+e^{k_{1}z_{B}^{2}} \right)}{R^{4}}\left( 2\left( 1-k_{0} \right)\left( V_{Lt-B}-u+2\delta\right)+k_{0}R \right); \frac{\partial f_{t3-B}}{\partial R_{Lt-B}}=0; \frac{\partial f_{t3-B}}{\partial C_{PL-t-B}}=-\lambda;$$

$$\frac{\partial f_{t3-B}}{\partial z_{B}}=\frac{2Gk_{1}z_{B} e^{k_{1}z_{B}^{2}}}{R^{4}}\left( ({V_{Lt-B}-u+2\delta)}^{2}(1-k_{0})+k_{0}R(V_{Lt-B}-u+2\delta) \right)\boldsymbol{.}$$

Concerning $f_{t4-B}(t,\boldsymbol{x}_{\boldsymbol{t-B}})$***:***

$$\frac{\partial f_{t4-B}}{\partial V_{Lt-B}}=1; \frac{\partial f_{t4-B}}{\partial R_{Lt-B}}=0; \frac{\partial f_{t4-B}}{\partial C_{PL-t-B}}=0; \frac{\partial f_{t4-B}}{\partial z_{B}}=0$$

All partial derivatives of $f_{t-B}\left( t\mathbf{,}\mathbf{x}_{\boldsymbol{t-B}} \right)$ is continuous and bounded $\forall t \in[t_{0},t_{1}]$. Notice that: $\frac{\partial f_{t3-B}}{\partial V_{Lt-B}}$ and $\frac{\partial f_{t3-B}}{\partial z_{B}}$ contain state-dependent denominators, but such denominators are nonzero even if $\left( V_{Lt-B}-u \right)\to0$, as $\delta$ establishes a boundary; exponential terms included in $\frac{\partial f_{t3-B}}{\partial V_{Lt-B}}$ and $\frac{\partial f_{t3-B}}{\partial z_{B}}$ are bounded, as $z_{B}$ is finite for remission states $\left( V_{Lt-B}-u \right)\to0$. Therefore, $f_{t-B}\left( t\mathbf{,}\mathbf{x}_{\boldsymbol{t-B}} \right)$ is globally Lipschitz $\forall t\in[t_{0},t_{1}]$. As $f_{t-B}\left( t\mathbf{,}\mathbf{x}_{\boldsymbol{t-B}} \right)$ is continuous, then they are also piece-wise continuous. Since$f_{t-B}\left( t\mathbf{,}\mathbf{x}_{\boldsymbol{t-B}} \right)$ is globally Lipschitz and piece-wise continuous, then $\mathbf{x}_{\boldsymbol{t-B}}\mathbf{=}f_{t-B}\left( t\mathbf{,}\mathbf{x}_{\boldsymbol{t-B}} \right)$ has a unique solution $\forall t\in[t_{0},t_{1}]$ for each set of initial conditions $\mathbf{x}_{\boldsymbol{0}\boldsymbol{t-B}}$and for each trajectory $u$. So, all partial derivatives of $f_{t-A}\left( t\mathbf{,}\mathbf{x}_{\boldsymbol{t-A}} \right)$ and $f_{t-B}\left( t\mathbf{,}\mathbf{x}_{\boldsymbol{t-B}} \right)$ are continuous and bounded $\forall t \in[t_{0},t_{1}]$. Therefore, $f_{t-A}\left( t\mathbf{,}\mathbf{x}_{\boldsymbol{t-A}} \right)$ and $f_{t-B}\left( t\mathbf{,}\mathbf{x}_{\boldsymbol{t-B}} \right)$ are globally Lipschitz $\forall t\in[t_{0},t_{1}]$. As $f_{t-A}\left( t\mathbf{,}\mathbf{x}_{\boldsymbol{t-A}} \right)$ and $f_{t-B}\left( t\mathbf{,}\mathbf{x}_{\boldsymbol{t-B}} \right)$ are continuous, then they are also piece-wise continuous. Since$f_{t-A}\left( t\mathbf{,}\mathbf{x}_{\boldsymbol{t-A}} \right)$ and $f_{t-B}\left( t\mathbf{,}\mathbf{x}_{\boldsymbol{t-B}} \right)$ are globally Lipschitz and piece-wise continuous, then $\mathbf{x}_{\boldsymbol{t-A}}\mathbf{=}f_{t-A}\left( t\mathbf{,}\mathbf{x}_{\boldsymbol{t-A}} \right)$ and $\mathbf{x}_{\boldsymbol{t-B}}\mathbf{=}f_{t-B}\left( t\mathbf{,}\mathbf{x}_{\boldsymbol{t-B}} \right)$ have a unique solution $\forall t\in[t_{0},t_{1}]$ for each set of initial conditions and for each trajectory $u$.

### 2.5 Equilibrium tumor states for $\boldsymbol{r>R}$.

The state $\mathbf{x}_{A}^{*}=\left[ \begin{matrix} V_{Lt-A}^{*} & R_{Lt-A}^{*} & C_{PL-t-A}^{*} & z_{A}^{*} \end{matrix} \right]^{T}$ for $f_{t-A}\left( t\mathbf{,}\mathbf{x}_{\boldsymbol{t-A}} \right)=0$*,* such that $V-u$ is ensured, is an equilibrium tumor state. Then,

$$\left\{ \begin{matrix} \dot{V}_{Lt-A}^{*}=\Lambda_{t1}+\nabla_{VV-C}\left( V_{Lt-A}^{*}-V_{t} \right)+\nabla_{VR}\left( R_{Lt-A}^{*}-R_{t} \right)-\varphi\left( V_{Lt-A}^{*}-V_{t} \right)C_{PL-t-A}^{*}=0, \\ \dot{R}_{Lt-A}^{*}=\Lambda_{t2}+\nabla_{RV}\left( V_{Lt-A}^{*}-V_{t} \right)+\nabla_{RR-C}\left( R_{Lt-A}^{*}-R_{t} \right)-\eta\left( R_{Lt-A}^{*}-R_{t} \right)C_{PL-t-A}^{*}=0, \\ \dot{C}_{PL-t-A}^{*}=-\lambda C_{PL-t-A}^{*}+\frac{G\left( M_{0}+e^{k_{1}z_{A}^{*2}} \right)}{\left( V_{Lt-A}^{*}-u+2\delta\right)^{2}}=0, \\ \dot{z}_{A}^{*}=V_{Lt-A}^{*}-u+\delta=0. \end{matrix} \right.$$

Concerning $V_{Lt-A}^{*}$*:*

$$V_{Lt-A}^{*}=u-\delta\Leftrightarrow V_{Lt-A}^{*}-u=-\delta.$$

Concerning $R_{Lt-A}^{*}$*:*

As

$$\Lambda_{t1}+\nabla_{VV-C}\left( u-\delta-V_{t} \right)+\nabla_{VR}\left( R_{Lt-A}^{*}-R_{t} \right)-\varphi\left( u-\delta-V_{t} \right)C_{PL-t-A}^{*}=0,$$

then,

| $\Lambda_{t1}+\nabla_{VV-C}\left( u-\delta-V_{t} \right)+\nabla_{VR}\left( R_{Lt-A}^{*}-R_{t} \right)=\varphi\left( u-\delta-V_{t} \right)C_{PL-t-A}^{*}.$ | (9) |
| --- | --- |

As

$$\Lambda_{t2}+\nabla_{RV}\left( u-\delta-V_{t} \right)+\nabla_{RR-C}\left( R_{Lt-A}^{*}-R_{t} \right)-\eta\left( R_{Lt-A}^{*}-R_{t} \right)C_{PL-t-A}^{*}=0,$$

then,

| $\Lambda_{t2}+\nabla_{RV}\left( u-\delta-V_{t} \right)+\nabla_{RR-C}\left( R_{Lt-A}^{*}-R_{t} \right)= \eta\left( R_{Lt-A}^{*}-R_{t} \right)C_{PL-t-A}^{*}$ | (10) |
| --- | --- |

Calculating (9)/(10), we obtain

$$\frac{\varphi\left( u-\delta-V_{t} \right)C_{PL-t-A}^{*}}{\eta\left( R_{Lt-A}^{*}-R_{t} \right)C_{PL-t-A}^{*}}=\frac{\Lambda_{t1}+\nabla_{VV-C}\left( u-\delta-V_{t} \right)+\nabla_{VR}\left( R_{Lt-A}^{*}-R_{t} \right)}{\Lambda_{t2}+\nabla_{RV}\left( u-\delta-V_{t} \right)+\nabla_{RR-C}\left( R_{Lt-A}^{*}-R_{t} \right)}$$

$$\Leftrightarrow\left( -\eta\nabla_{VR} \right)R_{Lt-A}^{*2}+\left( \varphi\nabla_{RR-C}\left( u-\delta-V_{t} \right)-\eta\Lambda_{t1}-\eta\nabla_{VV-C}\left( u-\delta-V_{t} \right)+2\eta R_{t}\nabla_{VR} \right)R_{Lt-A}^{*}+\left( \varphi\Lambda_{t2}\left( u-\delta-V_{t} \right)+\varphi\nabla_{RV}\left( u-\delta-V_{t} \right)^{2}-\varphi R_{t}\nabla_{RR-C}\left( u-\delta-V_{t} \right)+\eta R_{t}\Lambda_{t1}+\eta R_{t}\nabla_{VV-C}\left( u-\delta-V_{t} \right)-\eta\nabla_{VR}R_{t}^{2} \right)=0\Leftrightarrow R_{Lt-A}^{*2}+r_{10}R_{Lt-A}^{*}+r_{11}=0\Leftrightarrow R_{Lt-A}^{*}=-\frac{v_{10}}{2}\pm\frac{\sqrt{v_{10}^{2}-4v_{11}}}{2}.$$

with

$$v_{10}=\frac{-\varphi\nabla_{RR-C}\left( u-\delta-V_{t} \right)+\eta\Lambda_{t1}+\eta\nabla_{VV-C}\left( u-\delta-V_{t} \right)-2\eta R_{t}\nabla_{VR}}{\eta\nabla_{VR}}.$$

When $\delta\longrightarrow0$and $u=0$, then

$$v_{10}=\frac{\varphi V_{t}\nabla_{RR-C}+\eta\Lambda_{t1}-\eta V_{t}\nabla_{VV-C}-2\eta R_{t}\nabla_{VR}}{\eta\nabla_{VR}};$$

and

$$v_{11}=\frac{-\varphi\Lambda_{t2}\left( u-\delta-V_{t} \right)-\varphi\nabla_{RV}\left( u-\delta-V_{t} \right)^{2}+\varphi R_{t}\nabla_{RR-C}\left( u-\delta-V_{t} \right)-\eta R_{t}\Lambda_{t1}-\eta R_{t}\nabla_{VV-C}\left( u-\delta-V_{t} \right)+\eta\nabla_{VR}R_{t}^{2}}{\eta\nabla_{VR}}.$$

When $\delta\longrightarrow0$and considering $u=0$, then

$$v_{11}=\frac{\varphi V_{t}\Lambda_{t2}-\varphi{V_{t}^{2}\nabla}_{RV}-\varphi{V_{t}R}_{t}\nabla_{RR-C}-\eta R_{t}\Lambda_{t1}+\eta V_{t}R_{t}\nabla_{VV-C}+\eta\nabla_{VR}R_{t}^{2}}{\eta\nabla_{VR}}.$$

The equilibrium states demand: $v_{10}^{2}-4v_{11}\geq0.$

Concerning $C_{BH-t-A}^{*}$*:*

Calculating (9)+(10), we obtain

$$\Lambda_{t1}+\nabla_{VV-C}\left( u-\delta-V_{t} \right)+\nabla_{VR}\left( R_{Lt-A}^{*}-R_{t} \right)-\varphi\left( u-\delta-V_{t} \right)C_{PL-t-A}^{*}+\Lambda_{t2}+\nabla_{RV}\left( u-\delta-V_{t} \right)+\nabla_{RR-C}\left( R_{Lt-A}^{*}-R_{t} \right)-\eta\left( R_{Lt-A}^{*}-R_{t} \right)C_{PL-t-A}^{*}=0$$

$$\Leftrightarrow C_{PL-t-A}^{*}=\frac{\left( \Lambda_{t1}+\Lambda_{t2} \right)+\left( u-\delta-V_{t} \right)\left( \nabla_{RV}+\nabla_{VV-C} \right)+\left( R_{Lt-A}^{*}-R_{t} \right)\left( \nabla_{VR}+\nabla_{RR-C} \right)}{\varphi\left( u-\delta-V_{t} \right)+\eta\left( R_{Lt-A}^{*}-R_{t} \right)} .$$

When $\delta\longrightarrow0$and $u=0$, then

$$C_{PL-t-A}^{*}=\frac{\left( \Lambda_{t1}+\Lambda_{t2} \right)-V_{t}\left( \nabla_{RV}+\nabla_{VV-C} \right)-\left( \frac{v_{10}}{2}\pm\frac{\sqrt{v_{10}^{2}-4v_{11}}}{2}+R_{t} \right)\left( \nabla_{VR}+\nabla_{RR-C} \right)}{-\varphi V_{t}-\eta\left( \frac{v_{10}}{2}\pm\frac{\sqrt{v_{10}^{2}-4v_{11}}}{2}+R_{t} \right)}=\frac{v_{12}}{\varphi V_{t}+\eta\left( \frac{v_{10}}{2}\pm\frac{\sqrt{v_{10}^{2}-4v_{11}}}{2}+R_{t} \right)}$$

with

$$v_{12}=V_{t}\left( \nabla_{RV}+\nabla_{VV-C} \right)+\left( \frac{r_{10}}{2}\pm\frac{\sqrt{r_{10}^{2}-4r_{11}}}{2}+R_{t} \right)\left( \nabla_{VR}+\nabla_{RR-C} \right)-\left( \Lambda_{t1}+\Lambda_{t2} \right).$$

The equilibrium states also demand: $\varphi V_{t}+\eta\left( \frac{v_{10}}{2}\pm\frac{\sqrt{v_{10}^{2}-4v_{11}}}{2}+R_{t} \right)\neq0.$

Concerning $z_{A}^{*}$*:*

$$-\lambda C_{PL-t-A}^{*}+\frac{G\left( M_{0}+e^{k_{1}z_{A}^{*2}} \right)}{\left( V_{Lt-A}^{*}-u+2\delta\right)^{2}}=0\Leftrightarrow e^{k_{1}z_{A}^{*2}}=\frac{{\lambda\left( V_{Lt-A}^{*}-u+2\delta\right)}^{2}}{G}C_{PL-t-A}^{*}-M_{0}.$$

So,

$$z_{A}^{*}=\pm\sqrt{\frac{1}{k_{1}}\ln\left( \frac{{\lambda\delta}^{2}v_{12}}{G\varphi V_{t}+G\eta\left( \frac{v_{10}}{2}\pm\frac{\sqrt{v_{10}^{2}-4v_{11}}}{2}+R_{t} \right)}-M_{0} \right)}=\pm\sqrt{\frac{1}{k_{1}}\ln\left( v_{13}-M_{0} \right),}$$

with

$$r_{13}=\frac{{\lambda\delta}^{2}v_{12}}{G\varphi V_{t}+G\eta\left( \frac{v_{10}}{2}\pm\frac{\sqrt{v_{10}^{2}-4v_{11}}}{2}+R_{t} \right)}.$$

The equilibrium states demand: $\frac{1}{k_{1}}\ln\left( v_{13}-M_{0} \right)>0.$ Therefore, the state

$$\mathbf{x}_{A}^{*}=\left[ \begin{matrix} V_{Lt-A}^{*} & R_{Lt-A}^{*} & C_{PL-t-A}^{*} & z_{A}^{*} \end{matrix} \right]^{T} =\left[ \begin{matrix} u-\delta& -\frac{v_{10}}{2}\pm\frac{\sqrt{v_{10}^{2}-4v_{11}}}{2} & \frac{v_{12}}{\varphi V_{t}+\eta\left( \frac{v_{10}}{2}\pm\frac{\sqrt{v_{10}^{2}-4r_{11}}}{2}+R_{t} \right)} & \pm\sqrt{\frac{1}{k_{1}}\ln\left( v_{13}-M_{0} \right)} \end{matrix} \right]^{T}$$

is an equilibrium tumor state, where

$$v_{10}=\frac{\varphi V_{t}\nabla_{RR-C}+\eta\Lambda_{t1}-\eta V_{t}\nabla_{VV-C}-2\eta R_{t}\nabla_{VR}}{\eta\nabla_{VR}},$$

$$v_{11}=\frac{\varphi V_{t}\Lambda_{t2}-\varphi{V_{t}^{2}\nabla}_{RV}-\varphi{V_{t}R}_{t}\nabla_{RR-C}-\eta R_{t}\Lambda_{t1}+\eta V_{t}R_{t}\nabla_{VV-C}+\eta\nabla_{VR}R_{t}^{2}}{\eta\nabla_{VR}},$$

$$v_{12}=V_{t}\left( \nabla_{RV}+\nabla_{VV-C} \right)+\left( \frac{v_{10}}{2}\pm\frac{\sqrt{v_{10}^{2}-4v_{11}}}{2}+R_{t} \right)\left( \nabla_{VR}+\nabla_{RR-C} \right)-\left( \Lambda_{t1}+\Lambda_{t2} \right),$$

and

$$v_{13}=\frac{{\lambda\delta}^{2}v_{12}}{G\varphi V_{t}+G\eta\left( \frac{v_{10}}{2}\pm\frac{\sqrt{v_{10}^{2}-4v_{11}}}{2}+R_{t} \right)},$$

ensuring $v_{10}^{2}-4v_{11}\geq0, \varphi V_{t}+\eta\left( \frac{v_{10}}{2}\pm\frac{\sqrt{v_{10}^{2}-4v_{11}}}{2}+R_{t} \right)\neq0,$ and $\frac{1}{k_{1}}\ln\left( v_{13}-M_{0} \right)>0.$

### 2.6 Equilibrium tumor states for $\boldsymbol{|r|\leq R}$.

The state $\mathbf{x}_{B}^{*}=\left[ \begin{matrix} V_{Lt-B}^{*} & R_{Lt-B}^{*} & C_{PL-t-B}^{*} & z_{B}^{*} \end{matrix} \right]^{T}$ for $f_{t-B}\left( t\mathbf{,}\mathbf{x}_{\boldsymbol{t-B}} \right)=0$*,* such that $V-u$ is ensured, is an equilibrium tumor state. Then,

$$\left\{ \begin{matrix} \dot{V}_{Lt-B}^{*}=\Lambda_{t1}+\nabla_{VV-C}\left( V_{Lt-B}^{*}-V_{t} \right)+\nabla_{VR}\left( R_{Lt-B}^{*}-R_{t} \right)-\varphi\left( V_{Lt-B}^{*}-V_{t} \right)C_{PL-t-B}^{*}=0, \\ \dot{R}_{Lt-C}^{*}=\Lambda_{t2}+\nabla_{RV}\left( V_{Lt-B}^{*}-V_{t} \right)+\nabla_{RR-C}\left( R_{Lt-B}^{*}-R_{t} \right)-\eta\left( R_{Lt-B}^{*}-R_{t} \right)C_{PL-t-B}^{*}=0, \\ \dot{C}_{PL-t-B}^{*}=-\lambda C_{PL-t-B}^{*}+\frac{G\left( M_{0}+e^{k_{1}z_{B}^{*2}} \right)}{R^{4}} \left( ({V_{Lt-B}^{*}-u+2\delta)}^{2}(1-k_{0})+k_{0}R(V_{Lt-B}^{*}-u+2\delta) \right)=0, \\ \dot{z}_{B}^{*}=V_{Lt-B}^{*}-u+\delta=0. \end{matrix} \right.$$

Concerning $V_{Lt-B}^{*}$*:*

$$V_{Lt-B}^{*}=V_{Lt-A}^{*}=u-\delta.$$

Concerning $R_{Lt-B}^{*}$*:*

$$R_{Lt-B}^{*}=R_{Lt-A}^{*}=-\frac{v_{10}}{2}\pm\frac{\sqrt{v_{10}^{2}-4v_{11}}}{2},$$

with $v_{10}^{2}-4v_{11}>0.$

Concerning $C_{BH-t-B}^{*}$*:*

$$C_{PL-t-B}^{*}=C_{PL-t-A}^{*}=\frac{v_{12}}{\varphi V_{t}+\eta\left( \frac{v_{10}}{2}\pm\frac{\sqrt{v_{10}^{2}-4v_{11}}}{2}+R_{t} \right)},$$

with $\varphi V_{t}+\eta\left( \frac{v_{10}}{2}\pm\frac{\sqrt{v_{10}^{2}-4v_{11}}}{2}+R_{t} \right)\neq0.$

Concerning $z_{B}^{*}$*:*

$$\frac{G\left( M_{0}+e^{k_{1}z_{B}^{*2}} \right)}{R^{4}} \left( ({V_{Lt-B}^{*}-u+2\delta)}^{2}(1-k_{0})+k_{0}R(V_{Lt-CB}^{*}-u+2\delta) \right)=\lambda C_{PL-t-B}^{*}.$$

When $\delta\longrightarrow0$and $u=0$, then

$$\frac{G\left( M_{0}+e^{k_{1}z_{B}^{*2}} \right)\left( \delta^{2} (1-k_{0})+k_{0}R\delta\right)}{R^{4}}=\lambda C_{PL-t-B}^{*}\Longleftrightarrow e^{k_{1}z_{B}^{*2}}=\frac{{\lambda R}^{4}}{G\left( \delta^{2} \left( 1-k_{0} \right)+k_{0}R\delta\right)}C_{PL-t-B}^{*}-M_{0}.$$

So,

$$z_{B}^{*}=\pm\sqrt{\frac{1}{k_{1}}\ln\left( \frac{{\lambda R}^{4}}{G\left( \delta^{2} \left( 1-k_{0} \right)+k_{0}R\delta\right)}\left( \frac{v_{12}}{\varphi V_{t}+\eta\left( \frac{v_{10}}{2}\pm\frac{\sqrt{v_{10}^{2}-4v_{11}}}{2}+R_{t} \right)} \right)-M_{0} \right)}=\pm\sqrt{\frac{1}{k_{1}}\ln\left( v_{14}-M_{0} \right)},$$

with

$$r_{14}=\frac{{\lambda R}^{4}v_{12}}{G\left( \delta^{2} \left( 1-k_{0} \right)+k_{0}R\delta\right)\left( \varphi V_{t}+\eta\left( \frac{v_{10}}{2}\pm\frac{\sqrt{v_{10}^{2}-4v_{11}}}{2}+R_{t} \right) \right)},$$

and $\frac{1}{k_{1}}\ln\left( v_{14}-M_{0} \right)>0.$

Therefore, the state

$$\mathbf{x}_{B}^{*}=\left[ \begin{matrix} V_{Lt-B}^{*} & R_{Lt-B}^{*} & C_{PL-t-B}^{*} & z_{B}^{*} \end{matrix} \right]^{T} =\left[ \begin{matrix} u-\delta& -\frac{v_{10}}{2}\pm\frac{\sqrt{v_{10}^{2}-4v_{11}}}{2} & \frac{v_{12}}{\varphi V_{t}+\eta\left( \frac{v_{10}}{2}\pm\frac{\sqrt{v_{10}^{2}-4v_{11}}}{2}+R_{t} \right)} & \pm\sqrt{\frac{1}{k_{1}}\ln\left( v_{14}-M_{0} \right)} \end{matrix} \right]^{T}$$

is an equilibrium tumor state, where

$$v_{10}=\frac{\varphi V_{t}\nabla_{RR-C}+\eta\Lambda_{t1}-\eta V_{t}\nabla_{VV-C}-2\eta R_{t}\nabla_{VR}}{\eta\nabla_{VR}},$$

$$v_{11}=\frac{\varphi V_{t}\Lambda_{t2}-\varphi{V_{t}^{2}\nabla}_{RV}-\varphi{V_{t}R}_{t}\nabla_{RR-C}-\eta R_{t}\Lambda_{t1}+\eta V_{t}R_{t}\nabla_{VV-C}+\eta\nabla_{VR}R_{t}^{2}}{\eta\nabla_{VR}},$$

$$v_{12}=V_{t}\left( \nabla_{RV}+\nabla_{VV-C} \right)+\left( \frac{v_{10}}{2}\pm\frac{\sqrt{v_{10}^{2}-4v_{11}}}{2}+R_{t} \right)\left( \nabla_{VR}+\nabla_{RR-C} \right)-\left( \Lambda_{t1}+\Lambda_{t2} \right),$$

and

$$r_{14}=\frac{{\lambda R}^{4}v_{12}}{G\left( \delta^{2} \left( 1-k_{0} \right)+k_{0}R\delta\right)\left( \varphi V_{t}+\eta\left( \frac{v_{10}}{2}\pm\frac{\sqrt{v_{10}^{2}-4v_{11}}}{2}+R_{t} \right) \right)},$$

ensuring $v_{10}^{2}-4v_{11}>0, \varphi V_{t}+\eta\left( \frac{v_{10}}{2}\pm\frac{\sqrt{v_{10}^{2}-4v_{11}}}{2}+R_{t} \right)\neq0$ and $\frac{1}{k_{1}}\ln\left( v_{14}-M_{0} \right)>0.$

### 2.7 Stability of detumorization trajectories

The eigenvalues of the linearization about the equilibrium tumor state $\mathbf{x}_{A}^{*}$can be found solving:

$$\left. \det\left( J_{f_{t-A}}-\psi I \right) \right|_{x=x_{A}^{*}}=0\Leftrightarrow\left| \begin{matrix} \left( \frac{\partial f_{t1-A}}{\partial V_{Lt-A}}-\lambda\psi\right) & \nabla_{VR} & \frac{\partial f_{t1-A}}{\partial C_{PL-t-A}} & 0 \\ \nabla_{RV} & \left( \frac{\partial f_{t2-A}}{\partial R_{Lt-A}}-\psi I \right) & \frac{\partial f_{t2-A}}{\partial C_{PL-t-A}} & 0 \\ \frac{\partial f_{t3-A}}{\partial V_{Lt-A}} & 0 & \left( -\lambda-\psi I \right) & \frac{\partial f_{t3-A}}{\partial z_{A}} \\ 1 & 0 & 0 & -\psi I \end{matrix} \right|=0$$

$$\Leftrightarrow\lambda\psi^{4}+\left( \lambda^{2}-\left. \frac{\partial f_{t1-A}}{\partial V_{Lt-A}} \right|_{x=x_{A}^{*}}-\left. \frac{\partial f_{t2-A}}{\partial R_{Lt-A}} \right|_{x=x_{A}^{*}} \right)\psi^{3}+\left( -\nabla_{VR}\nabla_{RV}-\lambda\left. \frac{\partial f_{t1-A}}{\partial V_{Lt-A}} \right|_{x=x_{A}^{*}}-\lambda\left. \frac{\partial f_{t2-A}}{\partial R_{Lt-A}} \right|_{x=x_{A}^{*}}+\left. \frac{\partial f_{t1-A}}{\partial V_{Lt-A}} \right|_{x=x_{A}^{*}}\left. \frac{\partial f_{t2-A}}{\partial R_{Lt-A}} \right|_{x=x_{A}^{*}}+\left. \frac{\partial f_{t1-A}}{\partial C_{PL-t-A}} \right|_{x=x_{A}^{*}}\left. \frac{\partial f_{t3-A}}{\partial V_{Lt-A}} \right|_{x=x_{A}^{*}} \right)\psi^{2}+\left( {-{\lambda\nabla}_{VR}\nabla_{RV}-\nabla}_{VR}\left. \frac{\partial f_{t2-A}}{\partial C_{PL-t-A}} \right|_{x=x_{A}^{*}}\left. \frac{\partial f_{t3-A}}{\partial V_{Lt-A}} \right|_{x=x_{A}^{*}}+\lambda\left. \frac{\partial f_{t1-A}}{\partial V_{Lt-A}} \right|_{x=x_{A}^{*}}\left. \frac{\partial f_{t2-A}}{\partial R_{Lt-A}} \right|_{x=x_{A}^{*}}-\left. \frac{\partial f_{t1-A}}{\partial C_{PL-t-A}} \right|_{x=x_{A}^{*}}\left. \frac{\partial f_{t3-A}}{\partial z_{A}} \right|_{x=x_{A}^{*}}-\left. \frac{\partial f_{t1-A}}{\partial C_{PL-t-A}} \right|_{x=x_{A}^{*}}\left. \frac{\partial f_{t2-A}}{\partial R_{Lt-A}} \right|_{x=x_{A}^{*}}\left. \frac{\partial f_{t3-A}}{\partial V_{Lt-A}} \right|_{x=x_{A}^{*}} \right)\psi+\left( \left. \frac{\partial f_{t1-A}}{\partial C_{PL-t-A}} \right|_{x=x_{A}^{*}}\left. \frac{\partial f_{t2-A}}{\partial R_{Lt-A}} \right|_{x=x_{A}^{*}}\left. \frac{\partial f_{t3-A}}{\partial z_{A}} \right|_{x=x_{A}^{*}}-\nabla_{VR}\left. \frac{\partial f_{t2-A}}{\partial C_{PL-t-A}} \right|_{x=x_{A}^{*}}\left. \frac{\partial f_{t3-A}}{\partial z_{A}} \right|_{x=x_{A}^{*}} \right)=0$$

$$\Leftrightarrow\psi^{4}+\frac{a_{1}}{\lambda}\psi^{3}+\frac{a_{2}}{\lambda}\psi^{2}+\frac{a_{3}}{\lambda}\psi+\frac{a_{4}}{\lambda}=0,$$

with

$$a_{1}=\lambda^{2}-\left. \frac{\partial f_{t1-A}}{\partial V_{Lt-A}} \right|_{x=x_{A}^{*}}-\left. \frac{\partial f_{t2-A}}{\partial R_{Lt-A}} \right|_{x=x_{A}^{*}}=\lambda^{2}-\nabla_{VV-C}\boldsymbol{+}\varphi C_{PL-t-A}^{*}-\nabla_{RR-C}+\eta C_{PL-t-A}^{*}{=C}_{PL-t-A}^{*}\left( \varphi+\eta\right)-b_{1},$$

with $b_{1}=\nabla_{VV-C}+\nabla_{RR-C}-\lambda^{2};$

$$a_{2}=-\nabla_{VR}\nabla_{RV}-\lambda\left. \frac{\partial f_{t1-A}}{\partial V_{Lt-A}} \right|_{x=x_{A}^{*}}-\lambda\left. \frac{\partial f_{t2-A}}{\partial R_{Lt-A}} \right|_{x=x_{A}^{*}}+\left. \frac{\partial f_{t1-A}}{\partial V_{Lt-A}} \right|_{x=x_{A}^{*}}\left. \frac{\partial f_{t2-A}}{\partial R_{Lt-A}} \right|_{x=x_{A}^{*}}+\left. \frac{\partial f_{t1-A}}{\partial C_{PL-t-A}} \right|_{x=x_{A}^{*}}\left. \frac{\partial f_{t3-A}}{\partial V_{Lt-A}} \right|_{x=x_{A}^{*}}$$

$$a_{2}=-\nabla_{VR}\nabla_{RV}-\lambda\left( \nabla_{VV-C}\boldsymbol{-}\varphi C_{PL-t-A}^{*} \right)-\lambda\left( \nabla_{RR-C}-\eta C_{PL-t-A}^{*} \right)+\left( \nabla_{VV-C}\boldsymbol{-}\varphi C_{PL-t-A}^{*} \right)\left( \nabla_{RR-C}-\eta C_{PL-t-A}^{*} \right)-\varphi\left( V_{Lt-A}^{*}-V_{t} \right)\frac{2G\left( M_{0}+e^{k_{1}z_{A}^{* 2}} \right)}{\left( V_{Lt-A}^{*}-u+2\delta\right)^{3}}$$

$$a_{2}={\varphi\eta C_{PL-t-A}^{*2}+b_{2}C}_{PL-t-A}^{*}-\frac{2G{\varphi v}_{13}\left( V_{Lt-A}^{*}-V_{t} \right)}{\left( V_{Lt-A}^{*}-u+2\delta\right)^{3}}-b_{3},$$

with $b_{2}=\lambda\varphi++\lambda\eta-{\eta\nabla}_{VV-C}-\varphi\nabla_{RR-C}$, and $b_{3}=\nabla_{VR}\nabla_{RV}+\lambda\nabla_{VV-C}+\lambda\nabla_{RR-C}-\nabla_{VV-C}\nabla_{RR-C};$

$$a_{3}={-{\lambda\nabla}_{VR}\nabla_{RV}-\nabla}_{VR}\left. \frac{\partial f_{t2-A}}{\partial C_{PL-t-A}} \right|_{x=x_{A}^{*}}\left. \frac{\partial f_{t3-A}}{\partial V_{Lt-A}} \right|_{x=x_{A}^{*}}+\lambda\left. \frac{\partial f_{t1-A}}{\partial V_{Lt-A}} \right|_{x=x_{A}^{*}}\left. \frac{\partial f_{t2-A}}{\partial R_{Lt-A}} \right|_{x=x_{A}^{*}}-\left. \frac{\partial f_{t1-A}}{\partial C_{PL-t-A}} \right|_{x=x_{A}^{*}}\left. \frac{\partial f_{t3-A}}{\partial z_{A}} \right|_{x=x_{A}^{*}}-\left. \frac{\partial f_{t1-A}}{\partial C_{PL-t-A}} \right|_{x=x_{A}^{*}}\left. \frac{\partial f_{t2-A}}{\partial R_{Lt-A}} \right|_{x=x_{A}^{*}}\left. \frac{\partial f_{t3-A}}{\partial V_{Lt-A}} \right|_{x=x_{A}^{*}}$$

$$a_{3}=-{\lambda\nabla}_{VR}\nabla_{RV}-\nabla_{VR}\eta\left( R_{Lt-A}^{*}-R_{t} \right)\frac{2Gv_{13}}{\left( V_{Lt-A}^{*}-u+2\delta\right)^{3}}+\lambda\left( \nabla_{VV-C}\boldsymbol{-}\varphi C_{PL-t-A}^{*} \right)\left( \nabla_{RR-C}-\eta C_{PL-t-A}^{*} \right)+\varphi\left( V_{Lt-A}^{*}-V_{t} \right)\frac{2Gk_{1}z_{A}^{*} \left( v_{13}-M_{0} \right)}{\left( V_{Lt-A}^{*}-u+2\delta\right)^{2}}+\varphi\left( V_{Lt-A}^{*}-V_{t} \right)\left( \nabla_{RR-C}-\eta C_{PL-t-A}^{*} \right)\frac{2Gv_{13}}{\left( V_{Lt-A}^{*}-u+2\delta\right)^{3}}$$

$$a_{3}=\varphi\eta C_{PL-t-A}^{*2}-{b_{4}C}_{PL-t-A}^{*}+\frac{2Gv_{13} b_{6}}{\left( V_{Lt-A}^{*}-u+2\delta\right)^{3}}+\varphi\left( V_{Lt-A}^{*}-V_{t} \right)\frac{2Gk_{1}z_{A}^{*} \left( v_{13}-M_{0} \right)}{\left( V_{Lt-A}^{*}-u+2\delta\right)^{2}}+b_{7},$$

with $b_{4}=\varphi\nabla_{RR-C}+\lambda\eta\nabla_{VV-C}$, $b_{5}={\eta R_{t}\nabla}_{VR}-{\varphi V}_{t}\nabla_{RR-C},$ $b_{6}=\lambda\nabla_{VV-C}\nabla_{RR-C}-{\lambda\nabla}_{VR}\nabla_{RV},$ and

$$b_{7}={\varphi\nabla}_{RR-C}V_{Lt-A}^{*}-{\varphi\eta V}_{Lt-A}^{*}C_{PL-t-A}^{*}+\varphi\eta V_{t}C_{PL-t-A}^{*}-{\eta\nabla}_{VR}R_{Lt-A}^{*}+b_{5};$$

and

$$a_{4}=\left. \frac{\partial f_{t1-A}}{\partial C_{PL-t-A}} \right|_{x=x_{A}^{*}}\left. \frac{\partial f_{t2-A}}{\partial R_{Lt-A}} \right|_{x=x_{A}^{*}}\left. \frac{\partial f_{t3-A}}{\partial z_{A}} \right|_{x=x_{A}^{*}}-\nabla_{VR}\left. \frac{\partial f_{t2-A}}{\partial C_{PL-t-A}} \right|_{x=x_{A}^{*}}\left. \frac{\partial f_{t3-A}}{\partial z_{A}} \right|_{x=x_{A}^{*}}$$

$$a_{4}=-\varphi\left( V_{Lt-A}^{*}-V_{t} \right)\left( \nabla_{RR-C}-\eta C_{PL-t-A}^{*} \right)\left( \frac{2Gk_{1}z_{A}^{*} \left( v_{13}-M_{0} \right)}{\left( V_{Lt-A}^{*}-u+2\delta\right)^{2}} \right)$$

$$+{\eta\nabla}_{VR}\left( R_{Lt-A}^{*}-R_{t} \right)\left( \frac{2Gk_{1}z_{A}^{*} \left( r_{13}-M_{0} \right)}{\left( V_{Lt-A}^{*}-u+2\delta\right)^{2}} \right)=-\frac{2Gk_{1}z_{A}^{*} \left( v_{13}-M_{0} \right)b_{6}}{\left( V_{Lt-A}^{*}-u+2\delta\right)^{2}}.$$

Using the Routh-Hurwitz criterion, the fifth-order polynomial $P\left( \psi\right)=\psi^{4}+b_{1}\psi^{3}+b_{2}\psi^{2}+b_{3}\psi+b_{4}$has all roots in the left half-plane if and only if:

*Condition 1:* $a_{1}>0\Leftrightarrow C_{PL-t-A}^{*}\left( \varphi+\eta\right)-b_{1}>0\Leftrightarrow C_{PL-t-A}^{*}>\frac{\nabla_{VV-C}+\nabla_{RR-C}-\lambda^{2}}{\varphi+\eta}.$

*Condition 2:*$a_{3}>0$

$$\Leftrightarrow\varphi\eta C_{PL-t-A}^{*2}-{b_{4}C}_{PL-t-A}^{*}+\frac{2Gv_{13} b_{6}}{\left( V_{Lt-A}^{*}-u+2\delta\right)^{3}}+\varphi\left( V_{Lt-A}^{*}-V_{t} \right)\frac{2Gk_{1}z_{A}^{*} \left( v_{13}-M_{0} \right)}{\left( V_{Lt-A}^{*}-u+2\delta\right)^{2}}+b_{7}>0$$

As remission states require $V_{Lt-A}^{*}\to0$, $\left( V_{Lt-A}^{*}-u \right)\to0$ and $\delta\to0$, then we obtain $Gv_{13} b_{6}>0.$

*Condition 3:* $a_{4}>0$

$$\Leftrightarrow-\frac{2Gk_{1}z_{A}^{*} \left( v_{13}-M_{0} \right)b_{6}}{\left( V_{Lt-A}^{*}-u+2\delta\right)^{2}}>0\Leftrightarrow z_{A}^{*} \left( v_{13}-M_{0} \right)b_{6}<0.$$

*Condition 4 (Quartic condition):* $a_{1}a_{2}a_{3}-a_{1}^{2}a_{4}-\lambda a_{3}^{2}>0$

$$a_{1}a_{2}a_{3}=\left( C_{PL-t-A}^{*}\left( \varphi+\eta\right)-b_{1} \right)\left( {\varphi\eta C_{PL-t-A}^{*2}+b_{2}C}_{PL-t-A}^{*}-\frac{2G{\varphi v}_{13}\left( V_{Lt-A}^{*}-V_{t} \right)}{\left( V_{Lt-A}^{*}-u+2\delta\right)^{3}}-b_{3} \right)\left( \varphi\eta C_{PL-t-A}^{*2}-{b_{4}C}_{PL-t-A}^{*}+\frac{2Gv_{13} b_{6}}{\left( V_{Lt-A}^{*}-u+2\delta\right)^{3}}+\varphi\left( V_{Lt-A}^{*}-V_{t} \right)\frac{2Gk_{1}z_{A}^{*} \left( v_{13}-M_{0} \right)}{\left( V_{Lt-A}^{*}-u+2\delta\right)^{2}}+b_{7} \right).$$

$$a_{1}^{2}a_{4}=-2Gk_{1}\left( C_{PL-t-A}^{*}\left( \varphi+\eta\right)-b_{1} \right)^{2}\frac{z_{A}^{*} \left( v_{13}-M_{0} \right)b_{6}}{\left( V_{Lt-A}^{*}-u+2\delta\right)^{2}}.$$

$$\lambda a_{3}^{2}=\lambda\left( \varphi\eta C_{PL-t-A}^{*2}-{b_{4}C}_{PL-t-A}^{*}+\frac{2Gv_{13} b_{6}}{\left( V_{Lt-A}^{*}-u+2\delta\right)^{3}}+\varphi\left( V_{Lt-A}^{*}-V_{t} \right)\frac{2Gk_{1}z_{A}^{*} \left( v_{13}-M_{0} \right)}{\left( V_{Lt-A}^{*}-u+2\delta\right)^{2}}+b_{7} \right)^{2}.$$

So, the quartic condition is

$$\left( C_{PL-t-A}^{*}\left( \varphi+\eta\right)-b_{1} \right)\left( {\varphi\eta C_{PL-t-A}^{*2}+b_{2}C}_{PL-t-A}^{*}-\frac{2G{\varphi v}_{13}\left( V_{Lt-A}^{*}-V_{t} \right)}{\left( V_{Lt-A}^{*}-u+2\delta\right)^{3}}-b_{3} \right)\left( \varphi\eta C_{PL-t-A}^{*2}-{b_{4}C}_{PL-t-A}^{*}+\frac{2Gv_{13} b_{6}}{\left( V_{Lt-A}^{*}-u+2\delta\right)^{3}}+\varphi\left( V_{Lt-A}^{*}-V_{t} \right)\frac{2Gk_{1}z_{A}^{*} \left( v_{13}-M_{0} \right)}{\left( V_{Lt-A}^{*}-u+2\delta\right)^{2}}+b_{7} \right)+2Gk_{1}\left( C_{PL-t-A}^{*}\left( \varphi+\eta\right)-b_{1} \right)^{2}\frac{z_{A}^{*} \left( v_{13}-M_{0} \right)b_{6}}{\left( V_{Lt-A}^{*}-u+2\delta\right)^{2}}-\lambda\left( \varphi\eta C_{PL-t-A}^{*2}-{b_{4}C}_{PL-t-A}^{*}+\frac{2Gv_{13} b_{6}}{\left( V_{Lt-A}^{*}-u+2\delta\right)^{3}}+\varphi\left( V_{Lt-A}^{*}-V_{t} \right)\frac{2Gk_{1}z_{A}^{*} \left( v_{13}-M_{0} \right)}{\left( V_{Lt-A}^{*}-u+2\delta\right)^{2}}+b_{6} \right)^{2}>0.$$

If $V_{Lt-A}^{*}\to0$, $\left( V_{Lt-A}^{*}-u \right)\to0$ and $\delta\to0$, then

$$\left( C_{PL-t-A}^{*}\left( \varphi+\eta\right)-b_{1} \right)\left( -\frac{2G{\varphi v}_{13}\left( V_{Lt-A}^{*}-V_{t} \right)}{\left( V_{Lt-A}^{*}-u+2\delta\right)^{3}} \right)\left( \frac{2Gv_{13} b_{6}}{\left( V_{Lt-A}^{*}-u+2\delta\right)^{3}} \right)-\lambda\left( \frac{2Gv_{13} b_{6}}{\left( V_{Lt-A}^{*}-u+2\delta\right)^{3}} \right)^{2}>0.$$

$$4\varphi G^{2}v_{13}^{2}V_{t}b_{6}\left( C_{PL-t-A}^{*}\left( \varphi+\eta\right)-b_{1} \right)-4\lambda G^{2}v_{13}^{2}b_{6}^{2}>0$$

$$\Leftrightarrow\varphi V_{t}\left( C_{PL-t-A}^{*}\left( \varphi+\eta\right)+\lambda^{2}-\nabla_{VV-C}-\nabla_{RR-C} \right)>\lambda b_{6}.$$

The eigenvalues of the linearization about the equilibrium tumor state $\mathbf{x}_{B}^{*}$can be found solving:

$$\left. \det\left( J_{f_{t-B}}-\psi I \right) \right|_{x=\mathbf{x}_{B}^{*}}=0\Leftrightarrow\left| \begin{matrix} \left( \frac{\partial f_{t1-B}}{\partial V_{Lt-B}}-\lambda\psi\right) & \nabla_{VR} & \frac{\partial f_{t1-B}}{\partial C_{PL-t-B}} & 0 \\ \nabla_{RV} & \left( \frac{\partial f_{t2-B}}{\partial R_{Lt-B}}-\psi I \right) & \frac{\partial f_{t2-B}}{\partial C_{PL-t-B}} & 0 \\ \frac{\partial f_{t3-B}}{\partial V_{Lt-B}} & 0 & \left( -\lambda-\psi I \right) & \frac{\partial f_{t3-B}}{\partial z_{B}} \\ 1 & 0 & 0 & -\psi I \end{matrix} \right|=0$$

$$\Leftrightarrow\lambda\psi^{4}+\left( \lambda^{2}-\left. \frac{\partial f_{t1-B}}{\partial V_{Lt-B}} \right|_{x=x_{B}^{*}}-\left. \frac{\partial f_{t2-B}}{\partial R_{Lt-B}} \right|_{x=x_{B}^{*}} \right)\psi^{3}+\left( -\nabla_{VR}\nabla_{RV}-\lambda\left. \frac{\partial f_{t1-B}}{\partial V_{Lt-B}} \right|_{x=x_{B}^{*}}-\lambda\left. \frac{\partial f_{t2-B}}{\partial R_{Lt-B}} \right|_{x=x_{B}^{*}}+\left. \frac{\partial f_{t1-B}}{\partial V_{Lt-B}} \right|_{x=x_{B}^{*}}\left. \frac{\partial f_{t2-B}}{\partial R_{Lt-B}} \right|_{x=x_{B}^{*}}+\left. \frac{\partial f_{t1-B}}{\partial C_{PL-t-B}} \right|_{x=x_{B}^{*}}\left. \frac{\partial f_{t3-B}}{\partial V_{Lt-B}} \right|_{x=x_{B}^{*}} \right)\psi^{2}+\left( {-{\lambda\nabla}_{VR}\nabla_{RV}-\nabla}_{VR}\left. \frac{\partial f_{t2-B}}{\partial C_{PL-t-B}} \right|_{x=x_{B}^{*}}\left. \frac{\partial f_{t3-B}}{\partial V_{Lt-B}} \right|_{x=x_{B}^{*}}+\lambda\left. \frac{\partial f_{t1-B}}{\partial V_{Lt-B}} \right|_{x=x_{B}^{*}}\left. \frac{\partial f_{t2-B}}{\partial R_{Lt-B}} \right|_{x=x_{B}^{*}}-\left. \frac{\partial f_{t1-B}}{\partial C_{PL-t-B}} \right|_{x=x_{B}^{*}}\left. \frac{\partial f_{t3-B}}{\partial z_{B}} \right|_{x=x_{B}^{*}}-\left. \frac{\partial f_{t1-B}}{\partial C_{PL-t-B}} \right|_{x=x_{B}^{*}}\left. \frac{\partial f_{t2-B}}{\partial R_{Lt-B}} \right|_{x=x_{B}^{*}}\left. \frac{\partial f_{t3-B}}{\partial V_{Lt-B}} \right|_{x=x_{B}^{*}} \right)\psi+\left( \left. \frac{\partial f_{t1-B}}{\partial C_{PL-t-B}} \right|_{x=x_{B}^{*}}\left. \frac{\partial f_{t2-B}}{\partial R_{Lt-B}} \right|_{x=x_{B}^{*}}\left. \frac{\partial f_{t3-B}}{\partial z_{B}} \right|_{x=x_{B}^{*}}-\nabla_{VR}\left. \frac{\partial f_{t2-B}}{\partial C_{PL-t-B}} \right|_{x=x_{B}^{*}}\left. \frac{\partial f_{t3-B}}{\partial z_{B}} \right|_{x=x_{B}^{*}} \right)=0$$

$$\Leftrightarrow\psi^{4}+\frac{a_{5}}{\lambda}\psi^{3}+\frac{a_{6}}{\lambda}\psi^{2}+\frac{a_{7}}{\lambda}\psi+\frac{a_{8}}{\lambda}=0,$$

with

$$a_{5}=\lambda^{2}-\left. \frac{\partial f_{t1-B}}{\partial V_{Lt-B}} \right|_{x=x_{B}^{*}}-\left. \frac{\partial f_{t2-B}}{\partial R_{Lt-B}} \right|_{x=x_{B}^{*}}=\lambda^{2}-\left( \nabla_{VV-B}\boldsymbol{-}\varphi C_{PL-t-B}^{*} \right)-\left( \nabla_{RR-C}-\eta C_{PL-t-B}^{*} \right)$$

$$a_{5}=C_{PL-t-B}^{*}\left( \varphi+\eta\right)-b_{8},$$

with $b_{8}=\nabla_{VV-B}+\nabla_{RR-C}-\lambda^{2};$

$$a_{6}=-\nabla_{VR}\nabla_{RV}-\lambda\left. \frac{\partial f_{t1-B}}{\partial V_{Lt-B}} \right|_{x=x_{B}^{*}}-\lambda\left. \frac{\partial f_{t2-B}}{\partial R_{Lt-B}} \right|_{x=x_{B}^{*}}+\left. \frac{\partial f_{t1-B}}{\partial V_{Lt-B}} \right|_{x=x_{B}^{*}}\left. \frac{\partial f_{t2-B}}{\partial R_{Lt-B}} \right|_{x=x_{B}^{*}}+\left. \frac{\partial f_{t1-B}}{\partial C_{PL-t-B}} \right|_{x=x_{B}^{*}}\left. \frac{\partial f_{t3-B}}{\partial V_{Lt-B}} \right|_{x=x_{B}^{*}}$$

$$a_{6}=-\nabla_{VR}\nabla_{RV}-\lambda\left( \nabla_{VV-B}\boldsymbol{-}\varphi C_{PL-t-B}^{*} \right)-\lambda\left( \nabla_{RR-C}-\eta C_{PL-t-B}^{*} \right)+\left( \nabla_{VV-B}\boldsymbol{-}\varphi C_{PL-t-B}^{*} \right)\left( \nabla_{RR-C}-\eta C_{PL-t-B}^{*} \right)-\varphi\left( V_{Lt-B}^{*}-V_{t} \right)\frac{G\left( M_{0}+e^{k_{1}z_{B}^{*2}} \right)}{R^{4}}\left( 2\left( 1-k_{0} \right)\left( V_{Lt-B}^{*}-u+2\delta\right)+k_{0}R \right)$$

$$a_{6}=-\nabla_{VR}\nabla_{RV}-\lambda\nabla_{VV-B}+\lambda\varphi C_{PL-t-B}^{*}-\lambda\nabla_{RR-C}+\lambda\eta C_{PL-t-B}^{*}+\nabla_{VV-B}\nabla_{RR-C}-\eta\nabla_{VV-B}C_{PL-t-B}^{*}-\varphi\nabla_{RR-C}C_{PL-t-B}^{*}+\varphi\eta C_{PL-t-B}^{*2}-\frac{G{\varphi r}_{14}}{R^{4}}\left( V_{Lt-B}^{*}-V_{t} \right)\left( \left( 2-{2k}_{0} \right)\left( V_{Lt-B}^{*}-u+2\delta\right)+k_{0}R \right)$$

$$a_{6}=\varphi\eta C_{PL-t-B}^{*2}+{b_{9} C}_{PL-t-B}^{*}+\frac{G{\varphi r}_{14}}{R^{4}}\left( V_{Lt-B}^{*}-V_{t} \right)\left( \left( V_{Lt-B}^{*}-u \right)\left( 2-{2k}_{0} \right)+b_{10} \right)+b_{11},$$

with $b_{9}=\lambda\varphi+\lambda\eta-\eta\nabla_{VV-B}-\varphi\nabla_{RR-C}, b_{10}=4\delta-{4k}_{0}\delta+k_{0}R$, and

$$b_{11}=\nabla_{VV-B}\nabla_{RR-C}-\nabla_{VR}\nabla_{RV}-\lambda\nabla_{VV-B}-\lambda\nabla_{RR-C};$$

$$a_{7}={-{\lambda\nabla}_{VR}\nabla_{RV}-\nabla}_{VR}\left. \frac{\partial f_{t2-B}}{\partial C_{PL-t-B}} \right|_{x=x_{B}^{*}}\left. \frac{\partial f_{t3-B}}{\partial V_{Lt-B}} \right|_{x=x_{B}^{*}}+\lambda\left. \frac{\partial f_{t1-B}}{\partial V_{Lt-B}} \right|_{x=x_{B}^{*}}\left. \frac{\partial f_{t2-B}}{\partial R_{Lt-B}} \right|_{x=x_{B}^{*}}-\left. \frac{\partial f_{t1-B}}{\partial C_{PL-t-B}} \right|_{x=x_{B}^{*}}\left. \frac{\partial f_{t3-B}}{\partial z_{B}} \right|_{x=x_{B}^{*}}-\left. \frac{\partial f_{t1-B}}{\partial C_{PL-t-B}} \right|_{x=x_{B}^{*}}\left. \frac{\partial f_{t2-B}}{\partial R_{Lt-B}} \right|_{x=x_{B}^{*}}\left. \frac{\partial f_{t3-B}}{\partial V_{Lt-B}} \right|_{x=x_{B}^{*}}$$

$$a_{7}=-{\lambda\nabla}_{VR}\nabla_{RV}+\frac{{\eta G\nabla}_{VR}r_{14}}{R^{4}}\left( R_{Lt-B}^{*}-R_{t} \right)\left( \left( 2-{2k}_{0} \right)\left( V_{Lt-B}^{*}-u+2\delta\right)+k_{0}R \right)+\lambda\left( \nabla_{VV-B}\boldsymbol{-}\varphi C_{PL-t-B}^{*} \right)\left( \nabla_{RR-C}-\eta C_{PL-t-B}^{*} \right)+\varphi\left( V_{Lt-B}^{*}-V_{t} \right)\frac{2Gk_{1}z_{B}^{*}\left( v_{14}-M_{0} \right)}{R^{4}}\left( ({V_{Lt-B}^{*}-u+2\delta)}^{2}(1-k_{0})+k_{0}R(V_{Lt-B}^{*}-u+2\delta) \right)+\frac{\varphi Gr_{14}}{R^{4}}\left( V_{Lt-B}^{*}-V_{t} \right)\left( \nabla_{RR-C}-\eta C_{PL-t-B}^{*} \right)\left( \left( 2-{2k}_{0} \right)\left( V_{Lt-B}^{*}-u+2\delta\right)+k_{0}R \right)$$

$$a_{7}=\varphi\eta C_{PL-t-B}^{*2}-C_{PL-t-B}^{*}\left( b_{12}-\frac{\varphi\eta Gv_{14}}{R^{4}}\left( V_{Lt-B}^{*}-V_{t} \right)\left( \left( V_{Lt-B}^{*}-u \right)\left( 2-{2k}_{0} \right)+b_{10} \right) \right)+\frac{Gv_{14}}{R^{4}}\left( \left( V_{Lt-B}^{*}-u \right)\left( 2-{2k}_{0} \right)+b_{10} \right)\left( \varphi\nabla_{VR}\left( R_{Lt-B}^{*}-R_{t} \right)+\varphi\nabla_{RR-C}\left( V_{Lt-B}^{*}-V_{t} \right) \right)+\frac{2G\varphi k_{1}z_{B}^{*}\left( v_{14}-M_{0} \right)}{R^{4}}\left( V_{Lt-B}^{*}-V_{t} \right)\left( ({V_{Lt-B}^{*}-u+2\delta)}^{2}(1-k_{0})+k_{0}R(V_{Lt-B}^{*}-u+2\delta) \right)+b_{13},$$

with $b_{12}=\eta\nabla_{VV-B}+\varphi\nabla_{RR-C},$and $b_{13}={\lambda\nabla}_{VR}\nabla_{RV}+\nabla_{VV-B}\nabla_{RR-C};$

and

$$a_{8}=\left. \frac{\partial f_{t1-B}}{\partial C_{PL-t-B}} \right|_{x=x_{B}^{*}}\left. \frac{\partial f_{t2-B}}{\partial R_{Lt-B}} \right|_{x=x_{B}^{*}}\left. \frac{\partial f_{t3-B}}{\partial z_{B}} \right|_{x=x_{B}^{*}}-\nabla_{VR}\left. \frac{\partial f_{t2-B}}{\partial C_{PL-t-B}} \right|_{x=x_{B}^{*}}\left. \frac{\partial f_{t3-B}}{\partial z_{B}} \right|_{x=x_{B}^{*}}$$

$$a_{8}=-\frac{2G{\varphi k}_{1}z_{B}^{*}\left( v_{14}-M_{0} \right)}{R^{4}}\left( V_{Lt-B}^{*}-V_{t} \right)\left( \nabla_{RR-C}-\eta C_{PL-t-B}^{*} \right)\left( ({V_{Lt-B}^{*}-u+2\delta)}^{2}(1-k_{0})+k_{0}R(V_{Lt-B}^{*}-u+2\delta) \right)+\frac{2G\eta{\nabla_{VR}k}_{1}z_{B}^{*}\left( v_{14}-M_{0} \right)}{R^{4}}\left( R_{Lt-B}^{*}-R_{t} \right)\left( ({V_{Lt-B}^{*}-u+2\delta)}^{2}(1-k_{0})+k_{0}R(V_{Lt-B}^{*}-u+2\delta) \right)$$

$$a_{8}=\frac{2Gk_{1}z_{B}^{*}\left( v_{14}-M_{0} \right)}{R^{4}}\left( ({V_{Lt-B}^{*}-u+2\delta)}^{2}(1-k_{0})+k_{0}R(V_{Lt-B}^{*}-u+2\delta) \right)\left( \eta\nabla_{VR}\left( R_{Lt-B}^{*}-R_{t} \right)-\varphi\left( V_{Lt-B}^{*}-V_{t} \right)\left( \nabla_{RR-C}-\eta C_{PL-t-B}^{*} \right) \right).$$

Using the Routh-Hurwitz criterion,

*Condition 5:* $a_{5}>0\Leftrightarrow C_{PL-t-B}^{*}\left( \varphi+\eta\right)-b_{8}>0\Leftrightarrow C_{PL-t-B}^{*}>\frac{\nabla_{VV-B}+\nabla_{RR-C}-\lambda^{2}}{\varphi+\eta}.$

*Condition 6:*$a_{7}>0$

$$\Leftrightarrow\varphi\eta C_{PL-t-B}^{*2}-C_{PL-t-B}^{*}\left( b_{12}-\frac{\varphi\eta Gv_{14}}{R^{4}}\left( V_{Lt-B}^{*}-V_{t} \right)\left( \left( V_{Lt-B}^{*}-u \right)\left( 2-{2k}_{0} \right)+b_{10} \right) \right)$$

$$+\frac{Gv_{14}}{R^{4}}\left( \left( V_{Lt-B}^{*}-u \right)\left( 2-{2k}_{0} \right)+b_{10} \right)\left( \varphi\nabla_{VR}\left( R_{Lt-B}^{*}-R_{t} \right)+\varphi\nabla_{RR-C}\left( V_{Lt-B}^{*}-V_{t} \right) \right)$$

$$+\frac{2G\varphi k_{1}z_{B}^{*}\left( v_{14}-M_{0} \right)}{R^{4}}\left( V_{Lt-B}^{*}-V_{t} \right)\left( ({V_{Lt-B}^{*}-u+2\delta)}^{2}(1-k_{0})+k_{0}R(V_{Lt-B}^{*}-u+2\delta) \right)+b_{13}>0.$$

If $V_{Lt-B}^{*}\to0$, $\left( V_{Lt-B}^{*}-u \right)\to0$ and $\delta\to0$, then

$$\varphi\eta C_{PL-t-B}^{*2}-C_{PL-t-B}^{*}\left( b_{12}+\frac{\varphi\eta GV_{t}v_{14}b_{10}}{R^{4}} \right)+\frac{Gv_{14} b_{10}}{R^{4}}\left( \varphi\nabla_{VR}\left( R_{Lt-B}^{*}-R_{t} \right)-\varphi\nabla_{RR-C}V_{t} \right)+b_{13}>0.$$

*Condition 7:*$a_{8}>0$

$$\Leftrightarrow\frac{2Gk_{1}z_{B}^{*}\left( v_{14}-M_{0} \right)}{R^{4}}\left( ({V_{Lt-B}^{*}-u+2\delta)}^{2}(1-k_{0})+k_{0}R(V_{Lt-B}^{*}-u+2\delta) \right)\left( \eta\nabla_{VR}\left( R_{Lt-B}^{*}-R_{t} \right)-\varphi\left( V_{Lt-B}^{*}-V_{t} \right)\left( \nabla_{RR-C}-\eta C_{PL-t-B}^{*} \right) \right)>0$$

If $V_{Lt-B}^{*}\to0$, $\left( V_{Lt-B}^{*}-u \right)\to0$, then

$$\frac{2Gk_{1}z_{B}^{*}\left( v_{14}-M_{0} \right)}{R^{4}}\left( 4\delta^{2}(1-k_{0})+{2k}_{0}R\delta\right)\left( \eta\nabla_{VR}\left( R_{Lt-B}^{*}-R_{t} \right)+{\varphi V}_{t}\left( \nabla_{RR-C}-\eta C_{PL-t-B}^{*} \right) \right)>0.$$

*Condition 8 (Quartic condition):* $a_{5}a_{6}a_{7}-a_{5}^{2}a_{8}-\lambda a_{7}^{2}>0$

$$a_{5}a_{6}a_{7}=\left( C_{PL-t-B}^{*}\left( \varphi+\eta\right)-b_{8} \right)\left( \varphi\eta C_{PL-t-B}^{*2}+{b_{9} C}_{PL-t-B}^{*}+\frac{G{\varphi v}_{14}}{R^{4}}\left( V_{Lt-B}^{*}-V_{t} \right)\left( \left( V_{Lt-B}^{*}-u \right)\left( 2-{2k}_{0} \right)+b_{10} \right)+b_{11} \right)\times\left( \varphi\eta C_{PL-t-B}^{*2}-C_{PL-t-B}^{*}\left( b_{12}-\frac{\varphi\eta Gv_{14}}{R^{4}}\left( V_{Lt-B}^{*}-V_{t} \right)\left( \left( V_{Lt-B}^{*}-u \right)\left( 2-{2k}_{0} \right)+b_{10} \right) \right)+\frac{Gv_{14}}{R^{4}}\left( \left( V_{Lt-B}^{*}-u \right)\left( 2-{2k}_{0} \right)+b_{10} \right)\left( \varphi\nabla_{VR}\left( R_{Lt-B}^{*}-R_{t} \right)+\varphi\nabla_{RR-C}\left( V_{Lt-B}^{*}-V_{t} \right) \right)+\frac{2G\varphi k_{1}z_{B}^{*}\left( v_{14}-M_{0} \right)}{R^{4}}\left( V_{Lt-B}^{*}-V_{t} \right)\left( ({V_{Lt-B}^{*}-u+2\delta)}^{2}(1-k_{0})+k_{0}R(V_{Lt-B}^{*}-u+2\delta) \right)+b_{13} \right),$$

$$a_{5}^{2}a_{8}=\left( C_{PL-t-B}^{*}\left( \varphi+\eta\right)-b_{8} \right)^{2}$$

$$\times\left( a_{8}=\frac{2Gk_{1}z_{B}^{*}\left( v_{14}-M_{0} \right)}{R^{4}}\left( ({V_{Lt-B}^{*}-u+2\delta)}^{2}(1-k_{0})+k_{0}R(V_{Lt-B}^{*}-u+2\delta) \right)\left( \eta\nabla_{VR}\left( R_{Lt-B}^{*}-R_{t} \right)-\varphi\left( V_{Lt-B}^{*}-V_{t} \right)\left( \nabla_{RR-C}-\eta C_{PL-t-B}^{*} \right) \right) \right),$$

$$\lambda a_{7}^{2}=\lambda\left( \varphi\eta C_{PL-t-B}^{*2}-C_{PL-t-B}^{*}\left( b_{12}-\frac{\varphi\eta Gv_{14}}{R^{4}}\left( V_{Lt-B}^{*}-V_{t} \right)\left( \left( V_{Lt-B}^{*}-u \right)\left( 2-{2k}_{0} \right)+b_{10} \right) \right)+\frac{Gv_{14}}{R^{4}}\left( \left( V_{Lt-B}^{*}-u \right)\left( 2-{2k}_{0} \right)+b_{10} \right)\left( \varphi\nabla_{VR}\left( R_{Lt-B}^{*}-R_{t} \right)+\varphi\nabla_{RR-C}\left( V_{Lt-B}^{*}-V_{t} \right) \right)+\frac{2G\varphi k_{1}z_{B}^{*}\left( v_{14}-M_{0} \right)}{R^{4}}\left( V_{Lt-B}^{*}-V_{t} \right)\left( ({V_{Lt-B}^{*}-u+2\delta)}^{2}(1-k_{0})+k_{0}R(V_{Lt-B}^{*}-u+2\delta) \right)+b_{13} \right)^{2}.$$

So, the quartic condition is

$$\left( C_{PL-t-B}^{*}\left( \varphi+\eta\right)-b_{8} \right)\left( \varphi\eta C_{PL-t-B}^{*2}+{b_{9} C}_{PL-t-B}^{*}+\frac{G{\varphi v}_{14}}{R^{4}}\left( V_{Lt-B}^{*}-V_{t} \right)\left( \left( V_{Lt-B}^{*}-u \right)\left( 2-{2k}_{0} \right)+b_{10} \right)+b_{11} \right)\times\left( \varphi\eta C_{PL-t-B}^{*2}-C_{PL-t-B}^{*}\left( b_{12}-\frac{\varphi\eta Gv_{14}}{R^{4}}\left( V_{Lt-B}^{*}-V_{t} \right)\left( \left( V_{Lt-B}^{*}-u \right)\left( 2-{2k}_{0} \right)+b_{10} \right) \right)+\frac{Gv_{14}}{R^{4}}\left( \left( V_{Lt-B}^{*}-u \right)\left( 2-{2k}_{0} \right)+b_{10} \right)\left( \varphi\nabla_{VR}\left( R_{Lt-B}^{*}-R_{t} \right)+\varphi\nabla_{RR-C}\left( V_{Lt-B}^{*}-V_{t} \right) \right)+\frac{2G\varphi k_{1}z_{B}^{*}\left( v_{14}-M_{0} \right)}{R^{4}}\left( V_{Lt-B}^{*}-V_{t} \right)\left( ({V_{Lt-B}^{*}-u+2\delta)}^{2}(1-k_{0})+k_{0}R(V_{Lt-B}^{*}-u+2\delta) \right)+b_{13} \right)-\left( C_{PL-t-B}^{*}\left( \varphi+\eta\right)-b_{8} \right)^{2}$$

$$-\left( \frac{2Gk_{1}z_{B}^{*}\left( v_{14}-M_{0} \right)}{R^{4}}\left( ({V_{Lt-B}^{*}-u+2\delta)}^{2}(1-k_{0})+k_{0}R(V_{Lt-B}^{*}-u+2\delta) \right)\left( \eta\nabla_{VR}\left( R_{Lt-B}^{*}-R_{t} \right)-\varphi\left( V_{Lt-B}^{*}-V_{t} \right)\left( \nabla_{RR-C}-\eta C_{PL-t-B}^{*} \right) \right) \right)$$

$$-\lambda\left( \varphi\eta C_{PL-t-B}^{*2}-C_{PL-t-B}^{*}\left( b_{12}-\frac{\varphi\eta Gv_{14}}{R^{4}}\left( V_{Lt-B}^{*}-V_{t} \right)\left( \left( V_{Lt-B}^{*}-u \right)\left( 2-{2k}_{0} \right)+b_{10} \right) \right)+\frac{Gv_{14}}{R^{4}}\left( \left( V_{Lt-B}^{*}-u \right)\left( 2-{2k}_{0} \right)+b_{10} \right)\left( \varphi\nabla_{VR}\left( R_{Lt-B}^{*}-R_{t} \right)+\varphi\nabla_{RR-C}\left( V_{Lt-B}^{*}-V_{t} \right) \right)+\frac{2G\varphi k_{1}z_{B}^{*}\left( v_{14}-M_{0} \right)}{R^{4}}\left( V_{Lt-B}^{*}-V_{t} \right)\left( ({V_{Lt-B}^{*}-u+2\delta)}^{2}(1-k_{0})+k_{0}R(V_{Lt-B}^{*}-u+2\delta) \right)+b_{13} \right)^{2}>0.$$

If $V_{Lt-B}^{*}\to0$, $\left( V_{Lt-B}^{*}-u \right)\to0$ and $\delta\to0$, then

$$\left( C_{PL-t-B}^{*}\left( \varphi+\eta\right)-b_{8} \right)\left( \varphi\eta C_{PL-t-B}^{*2}+{b_{9} C}_{PL-t-B}^{*}-\frac{G{\varphi V_{t}v}_{14}b_{10}}{R^{4}}+b_{11} \right)\left( \varphi\eta C_{PL-t-B}^{*2}-C_{PL-t-B}^{*}\left( b_{12}+\frac{\varphi\eta GV_{t}v_{14}b_{10}}{R^{4}} \right)+\frac{Gv_{14}b_{10}}{R^{4}}\left( \varphi\nabla_{VR}\left( R_{Lt-B}^{*}-R_{t} \right)-\varphi\nabla_{RR-C}V_{t} \right)+b_{13} \right)>\left( C_{PL-t-B}^{*}\left( \varphi+\eta\right)-b_{8} \right)^{2}+\lambda\left( \varphi\eta C_{PL-t-B}^{*2}-C_{PL-t-B}^{*}\left( b_{12}+\frac{\varphi\eta G{V_{t}v}_{14} b_{10}}{R^{4}} \right)+\frac{Gv_{14}b_{10}}{R^{4}}\left( \varphi\nabla_{VR}\left( R_{Lt-B}^{*}-R_{t} \right)-\varphi\nabla_{RR-C}V_{t} \right)+b_{13} \right)^{2}.$$

### *3. Mathematical demonstrations related to cancer therapy with chemoresistance*

### 3.1 Model Linearization

The tumor growth model related to chemotherapy resistance was linearized, as follows:

| $\dot{N}_{1Lt}=\Lambda_{t10}+\nabla_{N1-N1}\left( N_{1Lt}-N_{1t} \right)+\nabla_{N1-N2}\left( N_{2Lt}-N_{2t} \right)+\nabla_{N1-R}\left( R_{Lt}-R_{t} \right),$ | (11) |
| --- | --- |
| $\dot{N}_{2Lt}=\Lambda_{t11}+\nabla_{N2-N2}\left( N_{2Lt}-N_{2t} \right)+\nabla_{N2-N1}\left( N_{1Lt}-N_{1t} \right)+\nabla_{N2-R}\left( R_{Lt}-R_{t} \right)$ | (12) |
| $\dot{R}_{Lt}=\Lambda_{t12}+\nabla_{R-N1}\left( N_{1Lt}-N_{1t} \right)+\nabla_{R-N2}\left( N_{2Lt}-N_{2t} \right)+\nabla_{RR}\left( R_{Lt}-R_{t} \right)$ | (13) |
| $\dot{C}_{Lt}=\dot{C}_{t}=-\lambda C_{t}+T_{t},$ | (14) |

where $\dot{N}_{1Lt}$, $\dot{N}_{2Lt}, C_{t}, T_{t}$ are the sensitive tumor tissue, the resistive tumor tissue, the vasculature capacity, the drug concentration and the drug administration, respectively, at instant time $t$,

$$\Lambda_{t10}=-\left( \tau_{1}+\xi_{1}\ln\left( \frac{N_{1t}+N_{2t}}{R_{t}} \right) \right)N_{1t} +\tau_{2}N_{2t}-\varphi N_{1t}C_{t};\Lambda_{t11}=-\left( \tau_{2}+\xi_{2}\ln\left( \frac{N_{1t}+N_{2t}}{R_{t}} \right) \right)N_{2t} +\tau_{1}N_{1t},$$

$$\Lambda_{t12}=b\left( N_{1t}+N_{2t} \right)-\left( \mu+d\left( N_{1t}+N_{2t} \right)^{\frac{2}{3}} \right)R_{t}-\eta R_{t}C_{t};$$

$$\nabla_{N1-N1}=-\left( \xi_{1}N_{1t}\frac{1}{\frac{N_{1t}+N_{2t}}{R_{t}}}\frac{1}{R_{t}}+\tau_{1}+\xi_{1}\ln\left( \frac{N_{1t}+N_{2t}}{R_{t}} \right) \right)-\varphi C_{t}=-\xi_{1}\left( \frac{N_{1t}}{N_{1t}+N_{2t}}+\ln\left( \frac{N_{1t}+N_{2t}}{R_{t}} \right) \right)-\tau_{1}-\varphi C_{t};$$

$$\nabla_{N1-N2}=-\xi_{1}N_{1t}\left( \frac{R_{t}}{N_{1t}+N_{2t}} \right)\frac{1}{R_{t}}+\tau_{2}=-\frac{\xi_{1}N_{1t}}{N_{1t}+N_{2t}}+\tau_{2}; \nabla_{N1-R}=\xi_{1}N_{1t}\left( \frac{R_{t}}{N_{1t}+N_{2t}} \right)\left( \frac{N_{1t}}{R_{t}^{2}} \right)=\frac{{\xi_{1}N}_{1t}^{2}}{R_{t}\left( N_{1t}+N_{2t} \right)};$$

$$\nabla_{N2-N2}=-\left( \xi_{2}N_{2t}\frac{1}{\frac{N_{1t}+N_{2t}}{R_{t}}}\frac{1}{R_{t}}+\tau_{1}+\xi_{2}\ln\left( \frac{N_{1t}+N_{2t}}{R_{t}} \right) \right)=-\xi_{1}\left( \frac{N_{2t}}{N_{1t}+N_{2t}}+\ln\left( \frac{N_{1t}+N_{2t}}{R_{t}} \right) \right)-\tau_{1};$$

$$\nabla_{N2-N1}=-\xi_{2}N_{2t}\left( \frac{R_{t}}{N_{1t}+N_{2t}} \right)\frac{1}{R_{t}}+\tau_{1}=-\frac{\xi_{2}N_{2t}}{N_{1t}+N_{2t}}+\tau_{1}; \nabla_{N2-R}=\xi_{2}N_{2t}\left( \frac{R_{t}}{N_{1t}+N_{2t}} \right)\left( \frac{N_{2t}}{R_{t}^{2}} \right)=\frac{{\xi_{2}N}_{2t}^{2}}{R_{t}\left( N_{1t}+N_{2t} \right)};$$

$$\nabla_{R-N1}=b-\frac{2}{3}{dR}_{t}{N_{1t}}^{-\frac{1}{3}}; \nabla_{R-N2}=b-\frac{2}{3}{dR}_{t}{N_{2t}}^{-\frac{1}{3}}; \nabla_{RR}=\mu+d\left( N_{1t}+N_{2t} \right)^{\frac{2}{3}}-\eta C_{t}.$$

Taking

$$\nabla_{N1-N2-C}=-\xi_{1}\left( \frac{N_{1t}}{N_{1t}+N_{2t}}+\ln\left( \frac{N_{1t}+N_{2t}}{R_{t}} \right) \right)-\tau_{1}$$

and

$$\nabla_{RR-C}= \mu+d\left( N_{1t}+N_{2t} \right)^{\frac{2}{3}},$$

and rewriting Eqs. (11), (12) and (13) then

| $\dot{N}_{1Lt}=\Lambda_{t10}+\nabla_{N1-N2-C}\left( N_{1Lt}-N_{1t} \right)+\nabla_{N1-N2}\left( N_{2Lt}-N_{2t} \right)+\nabla_{N1-R}\left( R_{Lt}-R_{t} \right)-\varphi\left( N_{1Lt}-N_{1t} \right)C_{t}$ | (15) |
| --- | --- |
| $\dot{N}_{2Lt}=\Lambda_{t11}+\nabla_{N2-N2}\left( N_{2Lt}-N_{2t} \right)+\nabla_{N2-N1}\left( N_{1Lt}-N_{1t} \right)+\nabla_{N2-R}\left( R_{Lt}-R_{t} \right)$ | (16) |
| $\dot{R}_{Lt}=\Lambda_{t12}+\nabla_{R-N1}\left( N_{1Lt}-N_{1t} \right)+\nabla_{R-N2}\left( N_{2Lt}-N_{2t} \right)+\nabla_{RR-C}\left( R_{Lt}-R_{t} \right)-\eta\left( R_{Lt}-R_{t} \right)C_{t}$ | (17) |
| $\dot{C}_{Lt}=\dot{C}_{t}=-\lambda C_{t}+T_{t},$ | (18) |

with

$$\Lambda_{t10}=-\left( \tau_{1}+\xi_{1}\ln\left( \frac{N_{1t}+N_{2t}}{R_{t}} \right) \right)N_{1t} +\tau_{2}N_{2t}-\varphi N_{1t}C_{t};\Lambda_{t11}=-\left( \tau_{2}+\xi_{2}\ln\left( \frac{N_{1t}+N_{2t}}{R_{t}} \right) \right)N_{2t} +\tau_{1}N_{1t};$$

$$\Lambda_{t12}=b\left( N_{1t}+N_{2t} \right)-\left( \mu+d\left( N_{1t}+N_{2t} \right)^{\frac{2}{3}} \right)R_{t}-\eta R_{t}C_{t}; \nabla_{N1-N1}=-\xi_{1}\left( \frac{N_{1t}}{N_{1t}+N_{2t}}+\ln\left( \frac{N_{1t}+N_{2t}}{R_{t}} \right) \right)-\tau_{1}-\varphi C_{t};$$

$$\nabla_{N1-N2}=-\frac{\xi_{1}N_{1t}}{N_{1t}+N_{2t}}+\tau_{2}; \nabla_{N1-N2-C}=-\xi_{1}\left( \frac{N_{1t}}{N_{1t}+N_{2t}}+\ln\left( \frac{N_{1t}+N_{2t}}{R_{t}} \right) \right)-\tau_{1}; \nabla_{N1-R}=\frac{{\xi_{1}N}_{1t}^{2}}{R_{t}\left( N_{1t}+N_{2t} \right)};$$

$$\nabla_{N2-N2}=-\xi_{1}\left( \frac{N_{2t}}{N_{1t}+N_{2t}}+\ln\left( \frac{N_{1t}+N_{2t}}{R_{t}} \right) \right)-\tau_{1}; \nabla_{N2-N1}=-\frac{\xi_{2}N_{2t}}{N_{1t}+N_{2t}}+\tau_{1}; \nabla_{N2-R}=\frac{{\xi_{2}N}_{2t}^{2}}{R_{t}\left( N_{1t}+N_{2t} \right)};$$

$$\nabla_{R-N1}=b-\frac{2}{3}{dR}_{t}{N_{1t}}^{-\frac{1}{3}}; \nabla_{R-N2}=b-\frac{2}{3}{dR}_{t}{N_{2t}}^{-\frac{1}{3}}; \nabla_{RR}=\mu+d\left( N_{1t}+N_{2t} \right)^{\frac{2}{3}}-\eta C_{t}; \nabla_{RR-C}= \mu+d\left( N_{1t}+N_{2t} \right)^{\frac{2}{3}}.$$

### 3.2 Closed-loop system for $\boldsymbol{r>R}$.

Taking $z_{A}=\int_{0}^{t} r dt=\int_{0}^{t} (N_{1Lt-A}+N_{2Lt-A}-u+\delta)dt$, where the subscript $A$ refers to the region where $r>R$, the PF dosing controller can be incorporated into a closed-loop system $\boldsymbol{f}_{\boldsymbol{t-A}}$ defined as (Figure S5B):

| ${\dot{\mathbf{x}}}_{\boldsymbol{t-A}}\mathbf{=}f_{t-A}\left( t\mathbf{,}\mathbf{x}_{\boldsymbol{t-A}} \right)\mathbf{=}\left[ \begin{matrix} f_{t10-A}(t,\mathbf{x}_{\boldsymbol{t-A}}) \\ f_{t11-A}(t,\mathbf{x}_{\boldsymbol{t-A}}) \\ f_{t12-A}(t,\mathbf{x}_{\boldsymbol{t-A}}) \\ f_{t13-A}(t,\mathbf{x}_{\boldsymbol{t-A}}) \\ f_{t14-A}(t,\mathbf{x}_{\boldsymbol{t-A}}) \end{matrix} \right]\mathbf{=}\left\{ \begin{matrix} \dot{N}_{1Lt-A}=\Lambda_{t10}+\nabla_{N1-N2-C}\left( N_{1Lt-A}-N_{1t} \right)+\nabla_{N1-N2}\left( N_{2Lt-A}-N_{2t} \right)+\nabla_{N1-R}\left( R_{Lt-A}-R_{t} \right)-\varphi\left( N_{1Lt-A}-N_{1t} \right)C_{PL-t-A}, \\ \dot{N}_{2Lt-A}=\Lambda_{t11}+\nabla_{N2-N2}\left( N_{2Lt-A}-N_{2t} \right)+\nabla_{N2-N1}\left( N_{1Lt-A}-N_{1t} \right)+\nabla_{N2-R}\left( R_{Lt-A}-R_{t} \right), \\ \dot{R}_{Lt-A}=\Lambda_{t12}+\nabla_{R-N1}\left( N_{1Lt-A}-N_{1t} \right)+\nabla_{R-N2}\left( N_{2Lt-A}-N_{2t} \right)+\nabla_{RR-C}\left( R_{Lt-A}-R_{t} \right)-\eta\left( R_{Lt-A}-R_{t} \right)C_{PL-t-A}, \\ \dot{C}_{PL-t-A}=-\lambda C_{PL-t-A}+T_{PL-t-A ,} \\ \dot{z}_{A}=N_{1Lt-A}+N_{2Lt-A}-u+\delta, \end{matrix}\mathbf{=}\left\{ \begin{matrix} \dot{N}_{1Lt-A}=\Lambda_{t10}+\nabla_{N1-N2-C}\left( N_{1Lt-A}-N_{1t} \right)+\nabla_{N1-N2}\left( N_{2Lt-A}-N_{2t} \right)+\nabla_{N1-R}\left( R_{Lt-A}-R_{t} \right)-\varphi\left( N_{1Lt-A}-N_{1t} \right)C_{PL-t-A}, \\ \dot{N}_{2Lt-A}=\Lambda_{t11}+\nabla_{N2-N2}\left( N_{2Lt-A}-N_{2t} \right)+\nabla_{N2-N1}\left( N_{1Lt-A}-N_{1t} \right)+\nabla_{N2-R}\left( R_{Lt-A}-R_{t} \right), \\ \dot{R}_{Lt-A}=\Lambda_{t12}+\nabla_{R-N1}\left( N_{1Lt-A}-N_{1t} \right)+\nabla_{R-N2}\left( N_{2Lt-A}-N_{2t} \right)+\nabla_{RR-C}\left( R_{Lt-A}-R_{t} \right)-\eta\left( R_{Lt-A}-R_{t} \right)C_{PL-t-A}, \\ \dot{C}_{PL-t-A}=-\lambda C_{PL-t-A}+\frac{G\left( M_{0}+e^{k_{1}z_{A}^{2}} \right)}{\left( N_{1Lt-A}+N_{2Lt-A}-u+2\delta\right)^{2}}, \\ \dot{z}_{A}=N_{1Lt-A}+N_{2Lt-A}-u+\delta. \end{matrix} \right. \right.$ | |
| --- | --- |
|  | (19) |

### 3.3 Closed-loop system for $\boldsymbol{|r|\leq R}$

Taking $z_{B}=\int_{0}^{t} r dt=\int_{0}^{t} (N_{1Lt-B}+N_{2Lt-B}-u+\delta)dt$, where the subscript $B$ refers to the region where $|r|\leq R$, the PF dosing controller can be incorporated into a closed-loop system $\boldsymbol{f}_{\boldsymbol{t-B}}$ defined as (Figure 5B):

| ${\dot{\mathbf{x}}}_{\boldsymbol{t-B}}\mathbf{=}f_{t-B}\left( t\mathbf{,}\mathbf{x}_{\boldsymbol{t-B}} \right)\mathbf{=}\left[ \begin{matrix} f_{t10-B}(t,\mathbf{x}_{\boldsymbol{t-B}}) \\ f_{t11-B}(t,\mathbf{x}_{\boldsymbol{t-B}}) \\ f_{t12-B}(t,\mathbf{x}_{\boldsymbol{t-B}}) \\ f_{t13-B}(t,\mathbf{x}_{\boldsymbol{t-B}}) \\ f_{t14-B}(t,\mathbf{x}_{\boldsymbol{t-B}}) \end{matrix} \right]\mathbf{=}\left\{ \begin{matrix} \dot{N}_{1Lt-B}=\Lambda_{t10}+\nabla_{N1-N2-C}\left( N_{1Lt-B}-N_{1t} \right)+\nabla_{N1-N2}\left( N_{2Lt-B}-N_{2t} \right)+\nabla_{N1-R}\left( R_{Lt-B}-R_{t} \right)-\varphi\left( N_{1Lt-B}-N_{1t} \right)C_{PL-t-B}, \\ \dot{N}_{2Lt-B}=\Lambda_{t11}+\nabla_{N2-N2}\left( N_{2Lt-B}-N_{2t} \right)+\nabla_{N2-N1}\left( N_{1Lt-B}-N_{1t} \right)+\nabla_{N2-R}\left( R_{Lt-B}-R_{t} \right), \\ \dot{R}_{Lt-B}=\Lambda_{t12}+\nabla_{R-N1}\left( N_{1Lt-B}-N_{1t} \right)+\nabla_{R-N2}\left( N_{2Lt-B}-N_{2t} \right)+\nabla_{RR-C}\left( R_{Lt-B}-R_{t} \right)-\eta\left( R_{Lt-B}-R_{t} \right)C_{PL-t-B}, \\ \dot{C}_{PL-t-B}=-\lambda C_{PL-t-B}+\frac{G\left( M_{0}+e^{k_{1}z_{B}^{2}} \right)}{R^{4}} \left( ({N_{1Lt-B}+N_{2Lt-B}-u+2\delta)}^{2}(1-k_{0})+k_{0}R\left( N_{1Lt-B}+N_{2Lt-B}-u+2\delta\right) \right). \\ \dot{z}_{B}=N_{1Lt-B}+N_{2Lt-B}-u+\delta. \end{matrix} \right.$ | |
| --- | --- |
|  | (20) |

### 3.4 Existence and uniqueness of solutions provided by the PF controller.

*Closed-loop system* $f_{t-A}\left( t,x_{t-A} \right):$

$$J_{f_{t}-A}=\left[ \begin{matrix} \frac{\partial f_{t10-A}}{\partial N_{1Lt-A}} & \frac{\partial f_{t10-A}}{\partial N_{2Lt-A}} & \frac{\partial f_{t10-A}}{\partial R_{Lt-A}} & \frac{\partial f_{t10-A}}{\partial C_{PL-t-A}} & \frac{\partial f_{t10-A}}{\partial z_{A}} \\ \frac{\partial f_{t11-A}}{\partial N_{1Lt-A}} & \frac{\partial f_{t11-A}}{\partial N_{2Lt-A}} & \frac{\partial f_{t11-A}}{\partial R_{Lt-A}} & \frac{\partial f_{t11-A}}{\partial C_{PL-t-A}} & \frac{\partial f_{t11-A}}{\partial z_{A}} \\ \frac{\partial f_{t12-A}}{\partial N_{1Lt-A}} & \frac{\partial f_{t12-A}}{\partial N_{2Lt-A}} & \frac{\partial f_{t12-A}}{\partial R_{Lt-A}} & \frac{\partial f_{t12-A}}{\partial C_{PL-t-A}} & \frac{\partial f_{t12-A}}{\partial z_{A}} \\ \frac{\partial f_{t13-A}}{\partial N_{1Lt-A}} & \frac{\partial f_{t13-A}}{\partial N_{2Lt-A}} & \frac{\partial f_{t13-A}}{\partial R_{Lt-A}} & \frac{\partial f_{t13-A}}{\partial C_{PL-t-A}} & \frac{\partial f_{t13-A}}{\partial z_{A}} \\ \frac{\partial f_{t14-A}}{\partial N_{1Lt-A}} & \frac{\partial f_{t14-A}}{\partial N_{2Lt-A}} & \frac{\partial f_{t14-A}}{\partial R_{Lt-A}} & \frac{\partial f_{t14-A}}{\partial C_{PL-t-A}} & \frac{\partial f_{t14-A}}{\partial z_{A}} \end{matrix} \right]=\left[ \begin{matrix} \frac{\partial f_{t10-A}}{\partial N_{1Lt-A}} & \nabla_{N1-N2} & \nabla_{N1-R} & \frac{\partial f_{t10-A}}{\partial C_{PL-t-A}} & 0 \\ \nabla_{N2-N1} & \nabla_{N2-N2} & \nabla_{N2-R} & 0 & 0 \\ \nabla_{R-N1} & \nabla_{R-N2} & \frac{\partial f_{t12-A}}{\partial R_{Lt-A}} & \frac{\partial f_{t12-A}}{\partial C_{PL-t-A}} & 0 \\ \frac{\partial f_{t13-A}}{\partial N_{1Lt-A}} & \frac{\partial f_{t13-A}}{\partial N_{2Lt-A}} & 0 & -\lambda& \frac{\partial f_{t13-A}}{\partial z_{A}} \\ 1 & 1 & 0 & 0 & 0 \end{matrix} \right].$$

Concerning $f_{t10-A}(t,\boldsymbol{x}_{\boldsymbol{t-A}})$***:***

$$\frac{\partial f_{t10-A}}{\partial N_{1Lt-A}}=\nabla_{N1-N2-C}-\varphi C_{PL-t-A}; \frac{\partial f_{t10-A}}{\partial N_{2Lt-A}}=\nabla_{N1-N2}; \frac{\partial f_{t10-A}}{\partial R_{Lt-A}}=\nabla_{N1-R};$$

$$\frac{\partial f_{t10-A}}{\partial C_{PL-t-A}}=-\varphi\left( N_{1Lt-A}-N_{1t} \right); \frac{\partial f_{t10-A}}{\partial z_{A}}=0.$$

Concerning $f_{t11-A}(t,\boldsymbol{x}_{\boldsymbol{t-A}})$***:***

$$\frac{\partial f_{t11-A}}{\partial N_{1Lt-A}}=\nabla_{N2-N1}; \frac{\partial f_{t11-A}}{\partial N_{2Lt-A}}=\nabla_{N2-N2}; \frac{\partial f_{t11-A}}{\partial R_{Lt-A}}=\nabla_{N2-R}; \frac{\partial f_{t11-A}}{\partial C_{PL-t-A}}=0; \frac{\partial f_{t11-A}}{\partial z_{A}}=0.$$

Concerning $f_{t12-A}(t,\boldsymbol{x}_{\boldsymbol{t-A}})$*:*

$$\frac{\partial f_{t12-A}}{\partial N_{1Lt-A}}=\nabla_{R-N1}; \frac{\partial f_{t12-A}}{\partial N_{2Lt-A}}=\nabla_{R-N2}; \frac{\partial f_{t12-A}}{\partial R_{Lt-A}}=\nabla_{RR-C}-\eta C_{PL-t-A};$$

$$\frac{\partial f_{t12-A}}{\partial C_{PL-t-A}}=-\eta\left( R_{Lt-A}-R_{t} \right); \frac{\partial f_{t12-A}}{\partial z_{A}}=0.$$

Concerning $f_{t13-A}(t,\boldsymbol{x}_{\boldsymbol{t-A}})$***:***

$$\frac{\partial f_{t13-A}}{\partial N_{1Lt-A}}=\frac{2G\left( M_{0}+e^{k_{1}z_{A}^{2}} \right)}{\left( N_{1Lt-A}+N_{2Lt-A}-u+2\delta\right)^{3}}; \frac{\partial f_{t13-A}}{\partial N_{2Lt-A}}=\frac{\partial f_{t13-A}}{\partial N_{1Lt-A}}=\frac{2G\left( M_{0}+e^{k_{1}z_{A}^{2}} \right)}{\left( N_{1Lt-A}+N_{2Lt-A}-u+2\delta\right)^{3}};$$

$$\frac{\partial f_{t13-A}}{\partial R_{Lt-A}}=0;$$

$$\frac{\partial f_{t13-A}}{\partial C_{PL-t-A}}=-\lambda; \frac{\partial f_{t13-A}}{\partial z_{A}}=\frac{2Gk_{1}z_{A} e^{k_{1}z_{A}^{2}}}{\left( N_{1Lt-A}+N_{2Lt-A}-u+2\delta\right)^{2}}.$$

Concerning $f_{t14-A}(t,\boldsymbol{x}_{\boldsymbol{t-A}})$***:***

$$\frac{\partial f_{t14-A}}{\partial N_{1Lt-A}}=1 ; \frac{\partial f_{t14-A}}{\partial N_{2Lt-A}}=1; \frac{\partial f_{t14-A}}{\partial R_{Lt-A}}=0; \frac{\partial f_{t14-A}}{\partial C_{PL-t-A}}=0; \frac{\partial f_{t14-A}}{\partial z_{A}}=0.$$

All partial derivatives of $f_{t-A}\left( t\mathbf{,}\mathbf{x}_{\boldsymbol{t-A}} \right)$ are continuous and bounded $\forall t \in[t_{0},t_{1}]$. Notice that: $\frac{\partial f_{t13-A}}{\partial N_{1Lt-A}} ,$ $\frac{\partial f_{t13-A}}{\partial N_{2Lt-A}}$and $\frac{\partial f_{t13-A}}{\partial z_{A}}$ contain state-dependent denominators, but such denominators are nonzero even if $\left( N_{1Lt-A}+N_{2Lt-A}-u \right)\to0$, as $\delta$ establishes a boundary; exponential terms included in $\frac{\partial f_{t13-A}}{\partial N_{1Lt-A}} ,$ $\frac{\partial f_{t13-A}}{\partial N_{2Lt-A}}$and $\frac{\partial f_{t13-A}}{\partial z_{A}}$ are bounded, as $z_{A}$ is finite for remission states $\left( N_{1Lt-A}+N_{2Lt-A}-u \right)\to0$. Therefore, $f_{t-A}\left( t\mathbf{,}\mathbf{x}_{\boldsymbol{t-A}} \right)$ is globally Lipschitz $\forall t\in[t_{0},t_{1}]$. As $f_{t-A}\left( t\mathbf{,}\mathbf{x}_{\boldsymbol{t-A}} \right)$ is continuous, then they are also piece-wise continuous. Since$f_{t-A}\left( t\mathbf{,}\mathbf{x}_{\boldsymbol{t-A}} \right)$ is globally Lipschitz and piece-wise continuous, then $\mathbf{x}_{\boldsymbol{t-A}}\mathbf{=}f_{t-A}\left( t\mathbf{,}\mathbf{x}_{\boldsymbol{t-A}} \right)$ has a unique solution $\forall t\in[t_{0},t_{1}]$ for each set of initial conditions $\mathbf{x}_{\boldsymbol{0}\boldsymbol{t-A}}$and for each trajectory $u$.

*Closed-loop system* $f_{t-B}\left( t,x_{t-B} \right):$

$$J_{f_{t}-B}=\left[ \begin{matrix} \frac{\partial f_{t10-B}}{\partial N_{1Lt-B}} & \frac{\partial f_{t10-B}}{\partial N_{2Lt-B}} & \frac{\partial f_{t10-B}}{\partial R_{Lt-B}} & \frac{\partial f_{t10-B}}{\partial C_{PL-t-B}} & \frac{\partial f_{t10-B}}{\partial z_{B}} \\ \frac{\partial f_{t11-B}}{\partial N_{1Lt-B}} & \frac{\partial f_{t11-B}}{\partial N_{2Lt-B}} & \frac{\partial f_{t11-B}}{\partial R_{Lt-B}} & \frac{\partial f_{t11-B}}{\partial C_{PL-t-B}} & \frac{\partial f_{t11-B}}{\partial z_{B}} \\ \frac{\partial f_{t12-B}}{\partial N_{1Lt-B}} & \frac{\partial f_{t12-B}}{\partial N_{2Lt-B}} & \frac{\partial f_{t12-B}}{\partial R_{Lt-B}} & \frac{\partial f_{t12-B}}{\partial C_{PL-t-B}} & \frac{\partial f_{t12-B}}{\partial z_{B}} \\ \frac{\partial f_{t13-B}}{\partial N_{1Lt-B}} & \frac{\partial f_{t13-B}}{\partial N_{2Lt-B}} & \frac{\partial f_{t13-B}}{\partial R_{Lt-B}} & \frac{\partial f_{t13-B}}{\partial C_{PL-t-B}} & \frac{\partial f_{t13-B}}{\partial z_{B}} \\ \frac{\partial f_{t14-B}}{\partial N_{1Lt-B}} & \frac{\partial f_{t14-B}}{\partial N_{2Lt-B}} & \frac{\partial f_{t14-B}}{\partial R_{Lt-B}} & \frac{\partial f_{t14-B}}{\partial C_{PL-t-B}} & \frac{\partial f_{t14-B}}{\partial z_{B}} \end{matrix} \right]=\left[ \begin{matrix} \frac{\partial f_{t10-B}}{\partial N_{1Lt-B}} & \nabla_{N1-N2} & \nabla_{N1-R} & \frac{\partial f_{t10-B}}{\partial C_{PL-t-B}} & 0 \\ \nabla_{N2-N1} & \nabla_{N2-N2} & \nabla_{N2-R} & 0 & 0 \\ \nabla_{R-N1} & \nabla_{R-N2} & \frac{\partial f_{t12-B}}{\partial R_{Lt-B}} & \frac{\partial f_{t12-B}}{\partial C_{PL-t-B}} & 0 \\ \frac{\partial f_{t13-B}}{\partial N_{1Lt-B}} & \frac{\partial f_{t13-B}}{\partial N_{2Lt-B}} & 0 & -\lambda& \frac{\partial f_{t13-B}}{\partial z_{B}} \\ 1 & 1 & 0 & 0 & 0 \end{matrix} \right].$$

Concerning $f_{t10-B}(t,\boldsymbol{x}_{\boldsymbol{t-B}})$***:***

$$\frac{\partial f_{t10-B}}{\partial N_{1Lt-B}}=\nabla_{N1-N2-C}-\varphi C_{PL-t-C}; \frac{\partial f_{t10-B}}{\partial N_{2Lt-B}}=\nabla_{N1-N2}; \frac{\partial f_{t10-B}}{\partial R_{Lt-B}}=\nabla_{N1-R};$$

$$\frac{\partial f_{t10-B}}{\partial C_{PL-t-B}}=-\varphi\left( N_{1Lt-C}-N_{1t} \right); \frac{\partial f_{t10-B}}{\partial z_{B}}=0.$$

Concerning $f_{t11-B}(t,\boldsymbol{x}_{\boldsymbol{t-B}})$***:***

$$\frac{\partial f_{t11-B}}{\partial N_{1Lt-B}}=\nabla_{N2-N1}; \frac{\partial f_{t11-B}}{\partial N_{2Lt-B}}=\nabla_{N2-N2}; \frac{\partial f_{t11-B}}{\partial R_{Lt-B}}=\nabla_{N2-R}; \frac{\partial f_{t11-B}}{\partial C_{PL-t-B}}=0; \frac{\partial f_{t11-B}}{\partial z_{B}}=0.$$

Concerning $f_{t12-B}(t,\boldsymbol{x}_{\boldsymbol{t-B}})$***:***

$$\frac{\partial f_{t12-B}}{\partial N_{1Lt-B}}=\nabla_{R-N1}; \frac{\partial f_{t12-B}}{\partial N_{2Lt-B}}=\nabla_{R-N2}; \frac{\partial f_{t12-B}}{\partial R_{Lt-B}}=\nabla_{RR-C}-\eta C_{PL-t-C};$$

$$\frac{\partial f_{t12-B}}{\partial C_{PL-t-B}}=-\eta\left( R_{Lt-C}-R_{t} \right); \frac{\partial f_{t12-B}}{\partial z_{B}}=0.$$

Concerning $f_{t13-B}(t,\boldsymbol{x}_{\boldsymbol{t-B}})$***:***

$$\frac{\partial f_{t13-B}}{\partial N_{1Lt-B}}=\frac{G\left( M_{0}+e^{k_{1}z_{B}^{2}} \right)}{R^{4}}\left( 2\left( 1-k_{0} \right)\left( N_{1Lt-B}+N_{2Lt-B}-u+2\delta\right)+k_{0}R \right);$$

$$\frac{\partial f_{t13-B}}{\partial N_{2Lt-B}}=\frac{\partial f_{t13-B}}{\partial N_{1Lt-B}}=\frac{G\left( M_{0}+e^{k_{1}z_{B}^{2}} \right)}{R^{4}}\left( 2\left( 1-k_{0} \right)\left( N_{1Lt-B}+N_{2Lt-B}-u+2\delta\right)+k_{0}R \right);$$

$$\frac{\partial f_{t13-B}}{\partial R_{Lt-B}}=0; \frac{\partial f_{t13-B}}{\partial C_{PL-t-B}}=-\lambda.$$

$$\frac{\partial f_{t13-B}}{\partial z_{B}}=\frac{2Gk_{1}z_{B} e^{k_{1}z_{B}^{2}}}{R^{4}}\left( ({N_{1Lt-B}+N_{2Lt-B}-u+2\delta)}^{2}(1-k_{0})+k_{0}R(N_{1Lt-B}+N_{2Lt-B}-u+2\delta) \right)\boldsymbol{.}$$

Concerning $f_{t14-B}(t,\boldsymbol{x}_{\boldsymbol{t-B}})$***:***

$$\frac{\partial f_{t14-B}}{\partial N_{1Lt-B}}=1 ; \frac{\partial f_{t14-B}}{\partial N_{2Lt-B}}=1; \frac{\partial f_{t14-B}}{\partial R_{Lt-B}}=0; \frac{\partial f_{t14-B}}{\partial C_{PL-t-B}}=0; \frac{\partial f_{t14-B}}{\partial z_{B}}=0.$$

All partial derivatives of $f_{t-B}\left( t\mathbf{,}\mathbf{x}_{\boldsymbol{t-B}} \right)$ is continuous and bounded $\forall t \in\left[ t_{0},t_{1} \right].$ Notice that: $\frac{\partial f_{t13-B}}{\partial N_{1Lt-B}} ,$ $\frac{\partial f_{t13-B}}{\partial N_{2Lt-B}}$and $\frac{\partial f_{t13-B}}{\partial z_{B}}$ contain state-dependent denominators, but such denominators are nonzero even if $\left( N_{1Lt-B}+N_{2Lt-B}-u \right)\to0$, as $\delta$ establishes a boundary; exponential terms included in $\frac{\partial f_{t13-B}}{\partial N_{1Lt-B}} ,$ $\frac{\partial f_{t13-B}}{\partial N_{2Lt-B}}$and $\frac{\partial f_{t13-B}}{\partial z_{B}}$ are bounded, as $z_{B}$ is finite for remission states $\left( N_{1Lt-B}+N_{2Lt-B}-u \right)\to0$. Therefore, $f_{t-B}\left( t\mathbf{,}\mathbf{x}_{\boldsymbol{t-B}} \right)$ is globally Lipschitz $\forall t\in[t_{0},t_{1}]$. As $f_{t-B}\left( t\mathbf{,}\mathbf{x}_{\boldsymbol{t-B}} \right)$ is continuous, then they are also piece-wise continuous. Since$f_{t-B}\left( t\mathbf{,}\mathbf{x}_{\boldsymbol{t-B}} \right)$ is globally Lipschitz and piece-wise continuous, then $\mathbf{x}_{\boldsymbol{t-B}}\mathbf{=}f_{t-B}\left( t\mathbf{,}\mathbf{x}_{\boldsymbol{t-B}} \right)$ has a unique solution $\forall t\in[t_{0},t_{1}]$ for each set of initial conditions $\mathbf{x}_{\boldsymbol{0}\boldsymbol{t-B}}$and for each trajectory $u$. So, all partial derivatives of $f_{t-A}\left( t\mathbf{,}\mathbf{x}_{\boldsymbol{t-A}} \right)$ and $f_{t-B}\left( t\mathbf{,}\mathbf{x}_{\boldsymbol{t-B}} \right)$ are continuous and bounded $\forall t \in[t_{0},t_{1}]$. Therefore, $f_{t-A}\left( t\mathbf{,}\mathbf{x}_{\boldsymbol{t-A}} \right)$ and $f_{t-B}\left( t\mathbf{,}\mathbf{x}_{\boldsymbol{t-B}} \right)$ are globally Lipschitz $\forall t\in[t_{0},t_{1}]$. As $f_{t-A}\left( t\mathbf{,}\mathbf{x}_{\boldsymbol{t-A}} \right)$ and $f_{t-B}\left( t\mathbf{,}\mathbf{x}_{\boldsymbol{t-B}} \right)$ are continuous, then they are also piece-wise continuous. Since$f_{t-A}\left( t\mathbf{,}\mathbf{x}_{\boldsymbol{t-A}} \right)$ and $f_{t-B}\left( t\mathbf{,}\mathbf{x}_{\boldsymbol{t-B}} \right)$ are globally Lipschitz and piece-wise continuous, then $\mathbf{x}_{\boldsymbol{t-A}}\mathbf{=}f_{t-A}\left( t\mathbf{,}\mathbf{x}_{\boldsymbol{t-A}} \right)$ and $\mathbf{x}_{\boldsymbol{t-B}}\mathbf{=}f_{t-B}\left( t\mathbf{,}\mathbf{x}_{\boldsymbol{t-B}} \right)$ have a unique solution $\forall t\in[t_{0},t_{1}]$ for each set of initial conditions and for each trajectory $u$.

### 3.5 Equilibrium tumor states for $\boldsymbol{r>R}$*.*

The state $\mathbf{x}_{A}^{*}=\left[ \begin{matrix} N_{1Lt-A}^{*} & N_{2Lt-A}^{*} & R_{Lt-A}^{*} & C_{PL-t-A}^{*} & z_{A}^{*} \end{matrix} \right]^{T}$ for $f_{t-A}\left( t\mathbf{,}\mathbf{x}_{\boldsymbol{t-A}} \right)=0$*,* such that $N_{1Lt-A}^{*}+N_{2Lt-A}^{*}-u=0$ is ensured, is an equilibrium tumor state. Then,

$$\left\{ \begin{matrix} \dot{N}_{1Lt-A}^{*}=\Lambda_{t10}+\nabla_{N1-N2-C}\left( N_{1Lt-A}^{*}-N_{1t} \right)+\nabla_{N1-N2}\left( N_{2Lt-A}^{*}-N_{2t} \right)+\nabla_{N1-R}\left( R_{Lt-A}^{*}-R_{t} \right)-\varphi\left( N_{1Lt-A}^{*}-N_{1t} \right)C_{PL-t-A}^{*}=0, \\ \dot{N}_{2Lt-A}^{*}=\Lambda_{t11}+\nabla_{N2-N2}\left( N_{2Lt-A}^{*}-N_{2t} \right)+\nabla_{N2-N1}\left( N_{1Lt-A}^{*}-N_{1t} \right)+\nabla_{N2-R}\left( R_{Lt-A}^{*}-R_{t} \right)=0, \\ \dot{R}_{Lt-A}^{*}=\Lambda_{t12}+\nabla_{R-N1}\left( N_{1Lt-A}^{*}-N_{1t} \right)+\nabla_{R-N2}\left( N_{2Lt-A}^{*}-N_{2t} \right)+\nabla_{RR-C}\left( R_{Lt-A}^{*}-R_{t} \right)-\eta\left( R_{Lt-A}^{*}-R_{t} \right)C_{PL-t-A}^{*}=0, \\ \dot{C}_{PL-t-A}^{*}=-\lambda C_{PL-t-A}^{*}+\frac{G\left( M_{0}+e^{k_{1}z_{A}^{*2}} \right)}{\left( N_{1Lt-A}^{*}+N_{2Lt-A}^{*}-u+2\delta\right)^{2}}=0, \\ \dot{z}_{A}^{*}=N_{1Lt-A}^{*}+N_{2Lt-A}^{*}-u+\delta=0. \end{matrix} \right.$$

Concerning $\dot{N}_{1Lt-A}^{*}$:

$$N_{1Lt-A}^{*}+N_{2Lt-A}^{*}=u-\delta\Leftrightarrow N_{1Lt-A}^{*}+N_{2Lt-A}^{*}-u=-\delta$$

| $\Lambda_{t10}+\nabla_{N1-N2-C}\left( N_{1Lt-A}^{*}-N_{1t} \right)+\nabla_{N1-N2}\left( N_{2Lt-A}^{*}-N_{2t} \right)+\nabla_{N1-R}\left( R_{Lt-A}^{*}-R_{t} \right)=\varphi\left( N_{1Lt-A}^{*}-N_{1t} \right)C_{PL-t-A}^{*}$ | (21) |
| --- | --- |
| $\Lambda_{t12}+\nabla_{R-N1}\left( N_{1Lt-A}^{*}-N_{1t} \right)+\nabla_{R-N2}\left( N_{2Lt-A}^{*}-N_{2t} \right)+\nabla_{RR-C}\left( R_{Lt-A}^{*}-R_{t} \right)=\eta\left( R_{Lt-A}^{*}-R_{t} \right)C_{PL-t-A}^{*}$ | (22) |

Calculating (21)/(22), we obtain

$$\frac{\varphi\left( N_{1Lt-A}^{*}-N_{1t} \right)}{\eta\left( R_{Lt-A}^{*}-R_{t} \right)}=\frac{\Lambda_{t10}+\nabla_{N1-N2-C}\left( N_{1Lt-A}^{*}-N_{1t} \right)+\nabla_{N1-N2}\left( N_{2Lt-A}^{*}-N_{2t} \right)+\nabla_{N1-R}\left( R_{Lt-A}^{*}-R_{t} \right)}{\Lambda_{t12}+\nabla_{R-N1}\left( N_{1Lt-A}^{*}-N_{1t} \right)+\nabla_{R-N2}\left( N_{2Lt-A}^{*}-N_{2t} \right)+\nabla_{RR-C}\left( R_{Lt-A}^{*}-R_{t} \right)}$$

$$\Longleftrightarrow N_{1Lt-A}^{*2}\left( \nabla_{R-N1}\varphi-\nabla_{R-N2}\varphi\right)+N_{1Lt-A}^{*}\left( \Lambda_{t12}\varphi-2\nabla_{R-N1}\varphi N_{1t}+\nabla_{R-N2}\varphi\left( -N_{2t}+u-\delta\right)+\nabla_{R-N2}\varphi N_{1t}-\nabla_{RR-C}\varphi R_{t}+\nabla_{N1-N2-C}\eta R_{t}-\nabla_{N1-N2}\eta R_{t}-\nabla_{N1-N2}\eta R_{t} \right)-\nabla_{N1-R}\eta R_{Lt-A}^{*2}+R_{Lt-A}^{*}\left( -\nabla_{RR-C}\varphi N_{1t}-\Lambda_{t10}+\nabla_{N1-N2-C}\eta N_{1t}-\nabla_{N1-N2}\eta\left( -N_{2t}+u-\delta\right)+2\nabla_{N1-R}\eta R_{t} \right)+\left( -\Lambda_{t12}\varphi N_{1t}+\nabla_{R-N1}\varphi{N_{1t}}^{2}-\nabla_{R-N2}\varphi N_{1t}\left( -N_{2t}+u-\delta\right)+\nabla_{RR-C}\varphi N_{1t}R_{t}+\Lambda_{t10}R_{t}-\nabla_{N1-N2-C}\eta N_{1t}R_{t}+\nabla_{N1-N2}\eta R_{t}\left( -N_{2t}+u-\delta\right)-\nabla_{N1-R}\eta{R_{t}}^{2}-\nabla_{N1-R}\eta{R_{t}}^{2} \right)=N_{1Lt-A}^{*}R_{Lt-A}^{*}\left( -\nabla_{RR-C}\varphi+\nabla_{N1-N2-C}\eta-\nabla_{N1-N2}\eta\right).$$

Using (22):

$$\nabla_{N2-N2}\left( -N_{1Lt-A}^{*}{-N}_{2t}+u-\delta\right)+\nabla_{N2-N1}\left( N_{1Lt-A}^{*}-N_{1t} \right)+\nabla_{N2-R}\left( R_{Lt-A}^{*}-R_{t} \right)=-\Lambda_{t11}$$

$$\Longleftrightarrow{R_{Lt-A}^{*}\nabla}_{N2-R}=-\Lambda_{t11}-\nabla_{N2-N2}\left( {-N}_{2t}+u-\delta\right)+\nabla_{N2-N1}N_{1t}+\nabla_{N2-R}R_{t}-N_{1Lt-A}^{*}\left( {-\nabla}_{N2-N2}+\nabla_{N2-N1} \right).$$

Taking

$$v_{20}=\nabla_{N2-N2}-\nabla_{N2-N1},$$

$$v_{21}=-\Lambda_{t11}-\nabla_{N2-N2}\left( {-N}_{2t}+u-\delta\right)+\nabla_{N2-N1}N_{1t}+\nabla_{N2-R}R_{t},$$

then,

$${R_{Lt-A}^{*}\nabla}_{N2-R}={v_{20}N}_{1Lt-A}^{*}+r_{21}\Longleftrightarrow R_{Lt-A}^{*}=\frac{v_{20}}{\nabla_{N2-R}}N_{1Lt-A}^{*}+\frac{r_{21}}{\nabla_{N2-R}}.$$

If we also take

$$v_{22}=\varphi\left( \nabla_{R-N1}-\nabla_{R-N2} \right),$$

$$v_{23}=\Lambda_{t12}\varphi-2\nabla_{R-N1}\varphi N_{1t}+\nabla_{R-N2}\varphi\left( -N_{2t}+u-\delta\right)+\nabla_{R-N2}\varphi N_{1t}-\nabla_{RR-C}\varphi R_{t}+\nabla_{N1-N2-C}\eta R_{t}-\nabla_{N1-N2}\eta R_{t}-\nabla_{N1-N2}\eta R_{t},$$

$$v_{24}=-\nabla_{RR-C}\varphi N_{1t}-\Lambda_{t10}+\nabla_{N1-N2-C}\eta N_{1t}-\nabla_{N1-N2}\eta\left( -N_{2t}+u-\delta\right)+2\nabla_{N1-R}\eta R_{t},$$

$$v_{25}=-\Lambda_{t12}\varphi N_{1t}+\nabla_{R-N1}\varphi{N_{1t}}^{2}-\nabla_{R-N2}\varphi N_{1t}\left( -N_{2t}+u-\delta\right)+\nabla_{RR-C}\varphi N_{1t}R_{t}+\Lambda_{t10}R_{t}-\nabla_{N1-N2-C}\eta N_{1t}R_{t}+\nabla_{N1-N2}\eta R_{t}\left( -N_{2t}+u-\delta\right)-\nabla_{N1-R}\eta{R_{t}}^{2}-\nabla_{N1-R}\eta{R_{t}}^{2},$$

$$v_{26}=-\nabla_{RR-C}\varphi+\nabla_{N1-N2-C}\eta-\nabla_{N1-N2}\eta,$$

then,

$$r_{22}N_{1Lt-A}^{*2}+r_{23}N_{1Lt-A}^{*}-\nabla_{N1-R}\eta\left( \frac{v_{20}}{\nabla_{N2-R}}N_{1Lt-A}^{*}+\frac{v_{21}}{\nabla_{N2-R}} \right)^{2}+\frac{v_{20}v_{24}}{\nabla_{N2-R}}N_{1Lt-A}^{*}+\frac{v_{21}v_{24}}{\nabla_{N2-R}}{+v}_{25}={r_{26}N}_{1Lt-A}^{*}\left( \frac{v_{20}}{\nabla_{N2-R}}N_{1Lt-A}^{*}+\frac{v_{21}}{\nabla_{N2-R}} \right)\Longleftrightarrow N_{1Lt-A}^{*2}\left( v_{22}-\nabla_{N1-R}\eta\left( \frac{v_{20}}{\nabla_{N2-R}} \right)^{2}-\frac{v_{20}v_{26}}{\nabla_{N2-R}} \right)+N_{1Lt-A}^{*}\left( v_{23}+\frac{v_{20}v_{24}}{\nabla_{N2-R}}-\frac{v_{21}v_{26}}{\nabla_{N2-R}} \right)=2\nabla_{N1-R}\eta\left( \frac{v_{20}}{\nabla_{N2-R}} \right)\left( \frac{v_{21}}{\nabla_{N2-R}} \right)+\nabla_{N1-R}\eta\left( \frac{v_{21}}{\nabla_{N2-R}} \right)^{2}-\frac{v_{21}v_{24}}{\nabla_{N2-R}}{-v}_{25}.$$

Talking also

$$v_{27}=v_{22}-\nabla_{N1-R}\eta\left( \frac{v_{20}}{\nabla_{N2-R}} \right)^{2}-\frac{v_{20}v_{26}}{\nabla_{N2-R}}, v_{28}=v_{23}+\frac{v_{20}v_{24}}{\nabla_{N2-R}}-\frac{v_{21}v_{26}}{\nabla_{N2-R}},$$

$$v_{29}=-2\nabla_{N1-R}\eta\left( \frac{v_{20}}{\nabla_{N2-R}} \right)\left( \frac{v_{21}}{\nabla_{N2-R}} \right)-\nabla_{N1-R}\eta\left( \frac{v_{21}}{\nabla_{N2-R}} \right)^{2}-\frac{v_{21}v_{24}}{\nabla_{N2-R}}{-v}_{25},$$

then we obtain

$$v_{27}N_{1Lt-A}^{*2}+v_{28}N_{1Lt-A}^{*}+v_{29}=0\Leftrightarrow N_{1Lt-A}^{*}=-\frac{v_{28}}{2v_{27}}\pm\frac{\sqrt{v_{28}^{2}-4v_{27}v_{29}}}{2v_{27}},$$

with ($\delta\longrightarrow0$and $u=0$)

$v_{20}=\nabla_{N2-N2}-\nabla_{N2-N1}; v_{21}=-\Lambda_{t11}+\nabla_{N2-N2}N_{2t}+\nabla_{N2-N1}N_{1t}+\nabla_{N2-R}R_{t},$;

$$v_{22}=\varphi\left( \nabla_{R-N1}-\nabla_{R-N2} \right);$$

$$v_{23}=\Lambda_{t12}\varphi-2\nabla_{R-N1}\varphi N_{1t}-\nabla_{R-N2}\varphi N_{2t}+\nabla_{R-N2}\varphi N_{1t}-\nabla_{RR-C}\varphi R_{t}+\nabla_{N1-N2-C}\eta R_{t}$$

$$-\nabla_{N1-N2}\eta R_{t}-\nabla_{N1-N2}\eta R_{t};$$

$$v_{24}=-\nabla_{RR-C}\varphi N_{1t}-\Lambda_{t10}+\nabla_{N1-N2-C}\eta N_{1t}+\nabla_{N1-N2}\eta N_{2t}+2\nabla_{N1-R}\eta R_{t};$$

$$r_{25}=-\Lambda_{t12}\varphi N_{1t}+\nabla_{R-N1}\varphi{N_{1t}}^{2}+\nabla_{R-N2}\varphi N_{1t}N_{2t}+\nabla_{RR-C}\varphi N_{1t}R_{t}+\Lambda_{t10}R_{t}-\nabla_{N1-N2-C}\eta N_{1t}R_{t}$$

$$-\nabla_{N1-N2}\eta R_{t}N_{2t}-\nabla_{N1-R}\eta{R_{t}}^{2}-\nabla_{N1-R}\eta{R_{t}}^{2};$$

$$v_{26}=-\nabla_{RR-C}\varphi+\nabla_{N1-N2-C}\eta-\nabla_{N1-N2}\eta.$$

The equilibrium states demand: $v_{28}^{2}-4v_{27}v_{29}>0$ and $v_{27}>0.$

Concerning $\dot{N}_{2Lt-A}^{*}$:

$$N_{2Lt}^{*}=-N_{1Lt}^{*}= \frac{v_{28}}{2v_{27}}\pm\frac{\sqrt{v_{28}^{2}-4v_{27}v_{29}}}{2v_{27}},$$

which also demands: $v_{28}^{2}-4v_{27}v_{29}>0$ and $v_{27}>0.$

Concerning $\dot{R}_{Lt-A}^{*}$:

$$R_{Lt-A}^{*}=\frac{v_{20}}{\nabla_{N2-R}}N_{1Lt-A}^{*}+\frac{v_{21}}{\nabla_{N2-R}}=\frac{v_{20}}{\nabla_{N2-R}}\left( -\frac{v_{28}}{2v_{27}}\pm\frac{\sqrt{v_{28}^{2}-4v_{27}v_{29}}}{2v_{27}} \right)+\frac{v_{21}}{\nabla_{N2-R}}$$

$$\Longleftrightarrow R_{Lt-A}^{*}=\left( \frac{v_{20}v_{28}}{{2\nabla}_{N2-R}v_{27}}+\frac{v_{21}}{\nabla_{N2-R}} \right)\pm\frac{v_{20}\sqrt{v_{28}^{2}-4v_{27}v_{29}}}{{2\nabla}_{N2-R}v_{27}},$$

which demands: $v_{28}^{2}-4v_{27}v_{29}>0$ and $\nabla_{N2-R}v_{27}>0.$

Concerning $C_{BH-t-A}^{*}$:

Calculating (21)+(22), we obtain

$$\Lambda_{t10}+\nabla_{N1-N2-C}\left( N_{1Lt-A}^{*}-N_{1t} \right)+\nabla_{N1-N2}\left( -N_{1Lt-A}^{*}-N_{2t}+u-\delta\right)+\nabla_{N1-R}\left( R_{Lt-A}^{*}-R_{t} \right)-\varphi\left( N_{1Lt-A}^{*}-N_{1t} \right)C_{PL-t-A}^{*}+\Lambda_{t12}+\nabla_{R-N1}\left( N_{1Lt-A}^{*}-N_{1t} \right)+\nabla_{R-N2}\left( -N_{1Lt-A}^{*}-N_{2t}+u-\delta\right)+\nabla_{RR-C}\left( R_{Lt-A}^{*}-R_{t} \right)-\eta\left( R_{Lt-A}^{*}-R_{t} \right)C_{PL-t-A}^{*}=0$$

$$\Longleftrightarrow-C_{PL-t-A}^{*}\left( \varphi N_{1Lt-A}^{*}-\varphi N_{1t}+\frac{v_{20}\eta}{\nabla_{N2-R}}N_{1Lt-A}^{*}+\frac{v_{21}\eta}{\nabla_{N2-R}}-\eta R_{t} \right)+N_{1Lt-A}^{*}\left( \nabla_{N1-N2-C}-\nabla_{N1-N2}++\frac{\nabla_{N1-R}v_{20}}{\nabla_{N2-R}}+\nabla_{R-N1}-\nabla_{R-N2}+\frac{\nabla_{RR-C}v_{20}}{\nabla_{N2-R}} \right)+\left( \Lambda_{t10}-\nabla_{N1-N2-C}N_{1t}+\nabla_{N1-N2}\left( -N_{2t}+u-\delta\right)+\frac{\nabla_{N1-R}v_{21}}{\nabla_{N2-R}}-\nabla_{N1-R}R_{t}+\Lambda_{t12}-\nabla_{R-N1}N_{1t}+\nabla_{R-N2}\left( -N_{2t}+u-\delta\right)+\frac{\nabla_{RR-C}v_{21}}{\nabla_{N2-R}}-\nabla_{RR-C}R_{t} \right)=0.$$

Taking

$$v_{27}=-\varphi N_{1t}+\frac{v_{21}\eta}{\nabla_{N2-R}}-\eta R_{t},v_{28}=\nabla_{N1-N2-C}-\nabla_{N1-N2}+\frac{\nabla_{N1-R}v_{20}}{\nabla_{N2-R}}+\nabla_{R-N1}-\nabla_{R-N2}+\frac{\nabla_{RR-C}v_{20}}{\nabla_{N2-R}},$$

$$v_{29}=\Lambda_{t10}-\nabla_{N1-N2-C}N_{1t}+\nabla_{N1-N2}\left( -N_{2t}+u-\delta\right)+\frac{\nabla_{N1-R}v_{21}}{\nabla_{N2-R}}-\nabla_{N1-R}R_{t}+\Lambda_{t12}-\nabla_{R-N1}N_{1t}$$

$$+\nabla_{R-N2}\left( -N_{2t}+u-\delta\right)+\frac{\nabla_{RR-C}v_{21}}{\nabla_{N2-R}}-\nabla_{RR-C}R_{t},$$

then,

$$-C_{PL-t-A}^{*}\left( N_{1Lt-A}^{*}\left( \varphi+\frac{v_{20}\eta}{\nabla_{N2-R}} \right)+v_{27} \right)+v_{28}N_{1Lt-A}^{*}+v_{29}=0$$

$$\Longleftrightarrow C_{PL-t-A}^{*}=\frac{v_{28}N_{1Lt-A}^{*}+v_{29}}{N_{1Lt-A}^{*}\left( \varphi+\frac{v_{20}\eta}{\nabla_{N2-R}} \right)+v_{27}}=\frac{-\frac{v_{28}^{2}}{2v_{27}}\pm\frac{v_{28}\sqrt{v_{28}^{2}-4v_{27}v_{29}}}{2v_{27}}+v_{29}}{\left( -\frac{v_{28}}{2v_{27}}\pm\frac{\sqrt{v_{28}^{2}-4v_{27}v_{29}}}{2v_{27}} \right)\left( \varphi+\frac{v_{20}\eta}{\nabla_{N2-R}} \right)+v_{27}}=\frac{v_{30}}{v_{31}},$$

with

$$v_{27}=-\varphi N_{1t}+\frac{v_{21}\eta}{\nabla_{N2-R}}-\eta R_{t}; v_{28}=\nabla_{N1-N2-C}-\nabla_{N1-N2}+\frac{\nabla_{N1-R}v_{20}}{\nabla_{N2-R}}+\nabla_{R-N1}-\nabla_{R-N2}+\frac{\nabla_{RR-C}v_{20}}{\nabla_{N2-R}};$$

$$v_{29}=\Lambda_{t10}-\nabla_{N1-N2-C}N_{1t}-\nabla_{N1-N2}N_{2t}+\frac{\nabla_{N1-R}v_{21}}{\nabla_{N2-R}}-\nabla_{N1-R}R_{t}+\Lambda_{t12}-\nabla_{R-N1}N_{1t}-\nabla_{R-N2}N_{2t}$$

$$+\frac{\nabla_{RR-C}v_{21}}{\nabla_{N2-R}}-\nabla_{RR-C}R_{t};$$

$$v_{30}=-\frac{v_{28}^{2}}{2v_{27}}\pm\frac{v_{28}\sqrt{v_{28}^{2}-4v_{27}v_{29}}}{2v_{27}}+v_{29}; v_{31}=\left( -\frac{v_{28}}{2v_{27}}\pm\frac{\sqrt{v_{28}^{2}-4v_{27}v_{29}}}{2v_{27}} \right)\left( \varphi+\frac{v_{20}\eta}{\nabla_{N2-R}} \right)+v_{27},$$

and ensuring

$v_{28}^{2}-4v_{27}v_{29}>0, v_{27}>0,$and $v_{31}>0.$

Concerning $z_{A}^{*}$:

$$-\lambda C_{PL-t-A}^{*}+\frac{G\left( M_{0}+e^{k_{1}z_{A}^{*2}} \right)}{\left( N_{1Lt-A}^{*}+N_{2Lt-A}^{*}-u+2\delta\right)^{2}}=0\Leftrightarrow z_{A}^{*}=\pm\sqrt{\frac{1}{k_{1}}\ln\left( \frac{{\lambda\delta}^{2}v_{30}}{Gv_{31}}-M_{0} \right).}$$

Therefore, the state

$$\mathbf{x}_{A}^{*}=\left[ \begin{matrix} N_{1Lt-A}^{*} & N_{2Lt-A}^{*} & R_{Lt-A}^{*} & C_{PL-t-A}^{*} & z_{A}^{*} \end{matrix} \right]^{T}$$

$$=\left[ \begin{matrix} -\frac{v_{28}}{2v_{27}}\pm\frac{\sqrt{v_{28}^{2}-4v_{27}v_{29}}}{2v_{27}} & \frac{v_{28}}{2v_{27}}\pm\frac{\sqrt{v_{28}^{2}-4v_{27}v_{29}}}{2v_{27}} & \left( \frac{v_{20}v_{28}}{{2\nabla}_{N2-R}v_{27}}+\frac{v_{21}}{\nabla_{N2-R}} \right)\pm\frac{v_{20}\sqrt{v_{28}^{2}-4v_{27}v_{29}}}{{2\nabla}_{N2-R}v_{27}} & \frac{v_{30}}{v_{31}} & \pm\sqrt{\frac{1}{k_{1}}\ln\left( \frac{{\lambda\delta}^{2}v_{30}}{Gv_{31}}-M_{0} \right)} \end{matrix} \right]$$

is an equilibrium tumor state.

### 3.6 Equilibrium tumor states for $\boldsymbol{|r|\leq R}$

The state $\mathbf{x}_{B}^{*}=\left[ \begin{matrix} N_{1Lt-B}^{*} & N_{2Lt-B}^{*} & R_{Lt-B}^{*} & C_{PL-t-B}^{*} & z_{B}^{*} \end{matrix} \right]^{T}$ for $f_{t-B}\left( t\mathbf{,}\mathbf{x}_{\boldsymbol{t-B}} \right)=0$*,* such that $N_{1Lt-B}^{*}+N_{2Lt-B}^{*}-u=0$ is ensured, is an equilibrium tumor state. Then,

$$\left\{ \begin{matrix} \dot{N}_{1Lt-B}^{*}=\Lambda_{t10}+\nabla_{N1-N2-C}\left( N_{1Lt-B}^{*}-N_{1t} \right)+\nabla_{N1-N2}\left( N_{2Lt-B}^{*}-N_{2t} \right)+\nabla_{N1-R}\left( R_{Lt-B}^{*}-R_{t} \right)-\varphi\left( N_{1Lt-B}^{*}-N_{1t} \right)C_{PL-t-B}^{*}=0, \\ \dot{N}_{2Lt-B}^{*}=\Lambda_{t11}+\nabla_{N2-N2}\left( N_{2Lt-B}^{*}-N_{2t} \right)+\nabla_{N2-N1}\left( N_{1Lt-B}^{*}-N_{1t} \right)+\nabla_{N2-R}\left( R_{Lt-B}^{*}-R_{t} \right)=0, \\ \dot{R}_{Lt-B}^{*}=\Lambda_{t12}+\nabla_{R-N1}\left( N_{1Lt-B}^{*}-N_{1t} \right)+\nabla_{R-N2}\left( N_{2Lt-B}^{*}-N_{2t} \right)+\nabla_{RR-C}\left( R_{Lt-B}^{*}-R_{t} \right)-\eta\left( R_{Lt-B}^{*}-R_{t} \right)C_{PL-t-B}^{*}=0, \\ \dot{C}_{PL-t-B}^{*}=-\lambda C_{PL-t-B}^{*}+\frac{G\left( M_{0}+e^{k_{1}z_{B}^{*2}} \right)}{R^{4}} \left( ({N_{1Lt-B}^{*}+N_{2Lt-B}^{*}-u+2\delta)}^{2}(1-k_{0})+k_{0}R(N_{1Lt-B}^{*}+N_{2Lt-B}^{*}-u+2\delta) \right)=0, \\ \dot{z}_{B}^{*}=N_{1Lt-B}^{*}+N_{2Lt-B}^{*}-u+\delta=0, \end{matrix} \right.$$

Concerning $N_{1Lt-B}^{*}$:

$$N_{1Lt-B}^{*}=N_{1Lt-A}^{*}=-\frac{v_{28}}{2v_{27}}\pm\frac{\sqrt{v_{28}^{2}-4v_{27}v_{29}}}{2v_{27}},$$

with $v_{28}^{2}-4v_{27}v_{29}>0$ and $v_{27}>0.$

Concerning $N_{2Lt-B}^{*}$:

$$N_{2Lt-B}^{*}=N_{2Lt-A}^{*}=\frac{v_{28}}{2v_{27}}\pm\frac{\sqrt{v_{28}^{2}-4v_{27}v_{29}}}{2v_{27}},$$

also ensuring $v_{28}^{2}-4v_{27}v_{29}>0$ and $v_{27}>0.$

Concerning $R_{Lt-B}^{*}$:

$$R_{Lt-B}^{*}=R_{Lt-A}^{*}=\left( \frac{v_{20}r_{28}}{{2\nabla}_{N2-R}v_{27}}+\frac{v_{21}}{\nabla_{N2-R}} \right)\pm\frac{v_{20}\sqrt{v_{28}^{2}-4v_{27}v_{29}}}{{2\nabla}_{N2-R}v_{27}},$$

with $v_{28}^{2}-4v_{27}v_{29}>0$ and $\nabla_{N2-R}v_{27}>0.$

Concerning $C_{BH-t-B}^{*}$:

$$C_{PL-t-B}^{*}=C_{PL-t-A}^{*}=\frac{v_{30}}{v_{31}},$$

with $v_{28}^{2}-4v_{27}v_{29}>0,$and $v_{31}>0$

Concerning $z_{B}^{*}$*:*

$$-\lambda C_{BH-t-B}^{*}+\frac{G\left( M_{0}+e^{k_{1}z_{B}^{*2}} \right)}{R^{4}} \left( ({N_{1Lt-B}^{*}+N_{2Lt-B}^{*}-u+2\delta)}^{2}(1-k_{0})+k_{0}R(N_{1Lt-B}^{*}+N_{2Lt-B}^{*}-u+2\delta) \right)=0.$$

When $\delta\longrightarrow0$and $u=0$, then

$$\frac{G\left( M_{0}+e^{k_{1}z_{B}^{*2}} \right)\left( \delta^{2} (1-k_{0})+k_{0}R\delta\right)}{R^{4}}=\lambda C_{PL-t-B}^{*}{\Leftrightarrow z}_{B}^{*}=\pm\sqrt{\frac{1}{k_{1}}\ln\left( \frac{{\lambda R}^{4}v_{30}}{G\left( \delta^{2} \left( 1-k_{0} \right)+k_{0}R\delta\right)v_{31}}-M_{0} \right)}$$

$$=\pm\sqrt{\frac{1}{k_{1}}\ln\left( v_{32}-M_{0} \right)},$$

with

$$v_{32}=\frac{{\lambda R}^{4}v_{30}}{G\left( \delta^{2} \left( 1-k_{0} \right)+k_{0}R\delta\right)v_{31}}$$

and $\frac{1}{k_{1}}\ln\left( v_{32}-M_{0} \right)>0.$

Therefore, the state

$$\mathbf{x}_{B}^{*}=\left[ \begin{matrix} -\frac{v_{28}}{2v_{27}}\pm\frac{\sqrt{v_{28}^{2}-4v_{27}v_{29}}}{2v_{27}} & \frac{v_{28}}{2v_{27}}\pm\frac{\sqrt{v_{28}^{2}-4v_{27}v_{29}}}{2v_{27}} & \left( \frac{v_{20}v_{28}}{{2\nabla}_{N2-R}v_{27}}+\frac{v_{21}}{\nabla_{N2-R}} \right)\pm\frac{v_{20}\sqrt{v_{28}^{2}-4v_{27}v_{29}}}{{2\nabla}_{N2-R}v_{27}} & \frac{v_{30}}{v_{31}} & \pm\sqrt{\frac{1}{k_{1}}\ln\left( v_{32}-M_{0} \right)} \end{matrix} \right]$$

is an equilibrium tumor state.

### 3.7 Stability of detumorization trajectories

The eigenvalues of the linearization about the equilibrium point $\mathbf{x}_{A}^{*}$can be founded solving:

$$\left. \det\left( J_{f_{t-A}}-\psi I \right) \right|_{x=x_{A}^{*}}=0\Leftrightarrow\left[ \begin{matrix} \left( \frac{\partial f_{t10-A}}{\partial N_{1Lt-A}}-\psi I \right) & \nabla_{N1-N2} & \nabla_{N1-R} & \frac{\partial f_{t10-A}}{\partial C_{PL-t-A}} & 0 \\ \nabla_{N2-N1} & \left( \nabla_{N2-N2}-\psi I \right) & \nabla_{N2-R} & 0 & 0 \\ \nabla_{R-N1} & \nabla_{R-N2} & \left( \frac{\partial f_{t12-A}}{\partial R_{Lt-A}}-\psi I \right) & \frac{\partial f_{t12-A}}{\partial C_{PL-t-A}} & 0 \\ \frac{\partial f_{t13-A}}{\partial N_{1Lt-A}} & \frac{\partial f_{t13-A}}{\partial N_{2Lt-A}} & 0 & \left( -\lambda-\psi I \right) & \frac{\partial f_{t13-A}}{\partial z_{A}} \\ 1 & 1 & 0 & 0 & -\psi I \end{matrix} \right]=0$$

$\Leftrightarrow\psi^{5}+d_{1}\psi^{4}+d_{2}\psi^{3}+d_{3}\psi^{2}+d_{4}\psi$+$d_{5}=0,$

with

$$d_{1}=\nabla_{N2-N2}-1+\left. \frac{\partial f_{t12-A}}{\partial R_{Lt-A}} \right|_{x=x_{A}^{*}};$$

$$d_{2}=\nabla_{N2-N2}\lambda-\nabla_{R-N1}-\nabla_{N2-R}\nabla_{R-N2}-\nabla_{N1-N2}\nabla_{N2-N1}+\lambda\left. \frac{\partial f_{t10-A}}{\partial N_{1Lt-A}} \right|_{x=x_{A}^{*}}-\nabla_{N2-N2}\left. \frac{\partial f_{t10-A}}{\partial N_{1Lt-A}} \right|_{x=x_{A}^{*}}+\lambda\left. \frac{\partial f_{t12-A}}{\partial R_{Lt-A}} \right|_{x=x_{A}^{*}}-\nabla_{N2-N2}\left. \frac{\partial f_{t12-A}}{\partial R_{Lt-A}} \right|_{x=x_{A}^{*}}-\left. \frac{\partial f_{t13-A}}{\partial N_{1Lt-A}} \right|_{x=x_{A}^{*}}\left. \frac{\partial f_{t10-A}}{\partial C_{PL-t-A}} \right|_{x=x_{A}^{*}};$$

$$d_{3}={-\nabla_{R-N1}\lambda-\nabla_{N2-R}\nabla_{R-N2}\lambda-\nabla_{N1-N2}\nabla_{N2-N1}\lambda+\nabla_{R-N1}\nabla_{N2-N2}+\nabla_{N2-R}\nabla_{N1-N2}\nabla_{R-N1}+\nabla_{N1-R}\nabla_{R-N2}\nabla_{N2-N1}-\nabla}_{N2-N2}\lambda\left. \frac{\partial f_{t10-A}}{\partial N_{1Lt-A}} \right|_{x=x_{A}^{*}}-\nabla_{N2-N2}\lambda\left. \frac{\partial f_{t12-A}}{\partial R_{Lt-A}} \right|_{x=x_{A}^{*}}+\nabla_{N2-R}\nabla_{R-N2}\left. \frac{\partial f_{t10-A}}{\partial N_{1Lt-A}} \right|_{x=x_{A}^{*}}+\nabla_{N1-N2}\nabla_{N2-N1}\left. \frac{\partial f_{t12-A}}{\partial R_{Lt-A}} \right|_{x=x_{A}^{*}}-\lambda\left. \frac{\partial f_{t10-A}}{\partial N_{1Lt-A}} \right|_{x=x_{A}^{*}}\left. \frac{\partial f_{t12-A}}{\partial R_{Lt-A}} \right|_{x=x_{A}^{*}}+\nabla_{N2-N2}\left. \frac{\partial f_{t10-A}}{\partial N_{1Lt-A}} \right|_{x=x_{A}^{*}}\left. \frac{\partial f_{t12-A}}{\partial R_{Lt-A}} \right|_{x=x_{A}^{*}}+\nabla_{N2-R}\lambda\left. \frac{\partial f_{t13-A}}{\partial N_{1Lt-A}} \right|_{x=x_{A}^{*}}\left. \frac{\partial f_{t12-A}}{\partial C_{PL-t-A}} \right|_{x=x_{A}^{*}}+\nabla_{N2-N1}\left. \frac{\partial f_{t13-A}}{\partial N_{1Lt-A}} \right|_{x=x_{A}^{*}}\left. \frac{\partial f_{t10-A}}{\partial C_{PL-t-A}} \right|_{x=x_{A}^{*}}+\nabla_{N1-R}\left. \frac{\partial f_{t13-A}}{\partial N_{1Lt-A}} \right|_{x=x_{A}^{*}}\left. \frac{\partial f_{t12-A}}{\partial C_{PL-t-A}} \right|_{x=x_{A}^{*}}+\nabla_{N2-N2}\left. \frac{\partial f_{t13-A}}{\partial N_{1Lt-A}} \right|_{x=x_{A}^{*}}\left. \frac{\partial f_{t10-A}}{\partial C_{PL-t-A}} \right|_{x=x_{A}^{*}}+\left. \frac{\partial f_{t10-A}}{\partial C_{PL-t-A}} \right|_{x=x_{A}^{*}}\left. \frac{\partial f_{t13-A}}{\partial z_{A}} \right|_{x=x_{A}^{*}}+\left. \frac{\partial f_{t13-A}}{\partial N_{1Lt-A}} \right|_{x=x_{A}^{*}}\left. \frac{\partial f_{t12-A}}{\partial R_{Lt-A}} \right|_{x=x_{A}^{*}}\left. \frac{\partial f_{t10-A}}{\partial C_{PL-t-A}} \right|_{x=x_{A}^{*}};$$

$$d_{4}=\nabla_{R-N1}\nabla_{N2-N2}\lambda{+\nabla_{N1-R}\nabla_{R-N2}\nabla_{N2-N1}\lambda+\nabla}_{N2-N2}\lambda\left. \frac{\partial f_{t10-A}}{\partial N_{1Lt-A}} \right|_{x=x_{A}^{*}}\left. \frac{\partial f_{t12-A}}{\partial R_{Lt-A}} \right|_{x=x_{A}^{*}}+\nabla_{N2-R}\nabla_{R-N2}\lambda\left. \frac{\partial f_{t10-A}}{\partial N_{1Lt-A}} \right|_{x=x_{A}^{*}}+\nabla_{N1-N2}\nabla_{N2-N1}\lambda\left. \frac{\partial f_{t12-A}}{\partial R_{Lt-A}} \right|_{x=x_{A}^{*}}-\nabla_{N2-R}\left. \frac{\partial f_{t12-A}}{\partial C_{PL-t-A}} \right|_{x=x_{A}^{*}}\left. \frac{\partial f_{t13-A}}{\partial z_{A}} \right|_{x=x_{A}^{*}}-\nabla_{N1-R}\nabla_{N2-N1}\left. \frac{\partial f_{t13-A}}{\partial N_{1Lt-A}} \right|_{x=x_{A}^{*}}\left. \frac{\partial f_{t12-A}}{\partial C_{PL-t-A}} \right|_{x=x_{A}^{*}}-\nabla_{N2-N1}\left. \frac{\partial f_{t10-A}}{\partial C_{PL-t-A}} \right|_{x=x_{A}^{*}}\left. \frac{\partial f_{t13-A}}{\partial z_{A}} \right|_{x=x_{A}^{*}}-\nabla_{N2-R}\nabla_{R-N1}\left. \frac{\partial f_{t13-A}}{\partial N_{1Lt-A}} \right|_{x=x_{A}^{*}}\left. \frac{\partial f_{t10-A}}{\partial C_{PL-t-A}} \right|_{x=x_{A}^{*}}-\nabla_{N2-R}\nabla_{N1-N2}\left. \frac{\partial f_{t13-A}}{\partial N_{1Lt-A}} \right|_{x=x_{A}^{*}}\left. \frac{\partial f_{t12-A}}{\partial C_{PL-t-A}} \right|_{x=x_{A}^{*}}-\nabla_{N1-R}\nabla_{N2-N2}\left. \frac{\partial f_{t13-A}}{\partial N_{1Lt-A}} \right|_{x=x_{A}^{*}}\left. \frac{\partial f_{t12-A}}{\partial C_{PL-t-A}} \right|_{x=x_{A}^{*}}-\nabla_{N2-R}\nabla_{R-N2}\left. \frac{\partial f_{t13-A}}{\partial N_{1Lt-A}} \right|_{x=x_{A}^{*}}\left. \frac{\partial f_{t10-A}}{\partial C_{PL-t-A}} \right|_{x=x_{A}^{*}}{-\nabla}_{N1-R}\left. \frac{\partial f_{t12-A}}{\partial C_{PL-t-A}} \right|_{x=x_{A}^{*}}\left. \frac{\partial f_{t13-A}}{\partial z_{A}} \right|_{x=x_{A}^{*}}-\nabla_{N2-N2}\left. \frac{\partial f_{t10-A}}{\partial C_{PL-t-A}} \right|_{x=x_{A}^{*}}\left. \frac{\partial f_{t13-A}}{\partial z_{A}} \right|_{x=x_{A}^{*}}{-\nabla}_{N2-R}\left. \frac{\partial f_{t10-A}}{\partial N_{1Lt-A}} \right|_{x=x_{A}^{*}}\left. \frac{\partial f_{t13-A}}{\partial N_{1Lt-A}} \right|_{x=x_{A}^{*}}\left. \frac{\partial f_{t12-A}}{\partial C_{PL-t-A}} \right|_{x=x_{A}^{*}}-\nabla_{N2-N2}\left. \frac{\partial f_{t13-A}}{\partial N_{1Lt-A}} \right|_{x=x_{A}^{*}}\left. \frac{\partial f_{t12-A}}{\partial R_{Lt-A}} \right|_{x=x_{A}^{*}}\left. \frac{\partial f_{t10-A}}{\partial C_{PL-t-A}} \right|_{x=x_{A}^{*}}+\nabla_{N2-N1}\left. \frac{\partial f_{t13-A}}{\partial N_{1Lt-A}} \right|_{x=x_{A}^{*}}\left. \frac{\partial f_{t12-A}}{\partial R_{Lt-A}} \right|_{x=x_{A}^{*}}\left. \frac{\partial f_{t10-A}}{\partial C_{PL-t-A}} \right|_{x=x_{A}^{*}}-\left. \left. \frac{\partial f_{t12-A}}{\partial R_{Lt-A}} \right|_{x=x_{A}^{*}}\frac{\partial f_{t10-A}}{\partial C_{PL-t-A}} \right|_{x=x_{A}^{*}}\left. \frac{\partial f_{t13-A}}{\partial z_{A}} \right|_{x=x_{A}^{*}};$$

$$d_{5}=\nabla_{N2-R}\nabla_{N1-N2}\nabla_{R-N1}\lambda+\nabla_{N1-R}\nabla_{N2-N1}\left. \frac{\partial f_{t12-A}}{\partial C_{PL-t-A}} \right|_{x=x_{A}^{*}}\left. \frac{\partial f_{t13-A}}{\partial z_{A}} \right|_{x=x_{A}^{*}}+\nabla_{N2-R}\nabla_{R-N1}\left. \frac{\partial f_{t10-A}}{\partial C_{PL-t-A}} \right|_{x=x_{A}^{*}}\left. \frac{\partial f_{t13-A}}{\partial z_{A}} \right|_{x=x_{A}^{*}}+\nabla_{N2-R}\nabla_{N1-N2}\left. \frac{\partial f_{t12-A}}{\partial C_{PL-t-A}} \right|_{x=x_{A}^{*}}\left. \frac{\partial f_{t13-A}}{\partial z_{A}} \right|_{x=x_{A}^{*}}+\nabla_{N1-R}\nabla_{N2-N2}\left. \frac{\partial f_{t12-A}}{\partial C_{PL-t-A}} \right|_{x=x_{A}^{*}}\left. \frac{\partial f_{t13-A}}{\partial z_{A}} \right|_{x=x_{A}^{*}}+\nabla_{N2-R}\nabla_{R-N2}\left. \frac{\partial f_{t10-A}}{\partial C_{PL-t-A}} \right|_{x=x_{A}^{*}}\left. \frac{\partial f_{t13-A}}{\partial z_{A}} \right|_{x=x_{A}^{*}}+\left. \frac{\partial f_{t12-A}}{\partial R_{Lt-A}} \right|_{x=x_{A}^{*}}\left. \frac{\partial f_{t10-A}}{\partial C_{PL-t-A}} \right|_{x=x_{A}^{*}}\left. \frac{\partial f_{t13-A}}{\partial z_{A}} \right|_{x=x_{A}^{*}}+\nabla_{N2-R}\left. \frac{\partial f_{t10-A}}{\partial N_{1Lt-A}} \right|_{x=x_{A}^{*}}\left. \frac{\partial f_{t12-A}}{\partial C_{PL-t-A}} \right|_{x=x_{A}^{*}}\left. \frac{\partial f_{t13-A}}{\partial z_{A}} \right|_{x=x_{A}^{*}}+\nabla_{N2-N2}\left. \frac{\partial f_{t12-A}}{\partial R_{Lt-A}} \right|_{x=x_{A}^{*}}\left. \frac{\partial f_{t10-A}}{\partial C_{PL-t-A}} \right|_{x=x_{A}^{*}}\left. \frac{\partial f_{t13-A}}{\partial z_{A}} \right|_{x=x_{A}^{*}}.$$

Using the Routh-Hurwitz criterion, the fifth-order polynomial $P\left( \psi\right)={\psi^{5}+d_{1}\psi}^{4}+d_{2}\psi^{3}+d_{3}\psi^{2}+d_{4}\psi++d_{5}$has all roots in the left half-plane if and only if:

*Condition 1:* $d_{1}>0.$

*Condition 2:* $d_{2}>0.$

*Condition 3:* $d_{3}>0.$

*Condition 4:* $d_{4}>0\boldsymbol{.}$

*Condition 5:* $d_{5}>0.$

*Condition 6:* $d_{1}d_{2}d_{3}-d_{1}^{2}d_{4}-d_{3}^{2}>0.$

*Condition 7:* $\left( d_{1}d_{4}-d_{5} \right)\left( d_{1}d_{2}d_{3}-d_{3}^{2}-d_{1}^{2}d_{4} \right)-d_{5}\left( d_{1}d_{2}-d_{3} \right)^{2}-d_{1}d_{5}^{2}>0.$

The eigenvalues of the linearization about the equilibrium tumor state $\mathbf{x}_{B}^{*}$can be found solving:

$$\left. \det\left( J_{f_{t-B}}-\psi I \right) \right|_{x=\mathbf{x}_{B}^{*}}=0\Leftrightarrow\left[ \begin{matrix} \left( \frac{\partial f_{t10-B}}{\partial N_{1Lt-B}}-\psi I \right) & \nabla_{N1-N2} & \nabla_{N1-R} & \frac{\partial f_{t10-B}}{\partial C_{PL-t-B}} & 0 \\ \nabla_{N2-N1} & \left( \nabla_{N2-N2}-\psi I \right) & \nabla_{N2-R} & 0 & 0 \\ \nabla_{R-N1} & \nabla_{R-N2} & \left( \frac{\partial f_{t12-B}}{\partial R_{Lt-B}}-\psi I \right) & \frac{\partial f_{t12-B}}{\partial C_{PL-t-B}} & 0 \\ \frac{\partial f_{t13-B}}{\partial N_{1Lt-B}} & \frac{\partial f_{t13-B}}{\partial N_{2Lt-B}} & 0 & \left( -\lambda-\psi I \right) & \frac{\partial f_{t13-B}}{\partial z_{B}} \\ 1 & 1 & 0 & 0 & -\psi I \end{matrix} \right]=0$$

$\Leftrightarrow\psi^{5}+d_{6}\psi^{4}+d_{7}\psi^{3}+d_{8}\psi^{2}+d_{9}\psi$+$d_{10}=0,$

with

$$d_{6}=\nabla_{N2-N2}-1+\left. \frac{\partial f_{t12-B}}{\partial R_{Lt-B}} \right|_{x=x_{B}^{*}};$$

$$d_{7}=\nabla_{N2-N2}\lambda-\nabla_{R-N1}-\nabla_{N2-R}\nabla_{R-N2}-\nabla_{N1-N2}\nabla_{N2-N1}+\lambda\left. \frac{\partial f_{t10-B}}{\partial N_{1Lt-B}} \right|_{x=x_{B}^{*}}-\nabla_{N2-N2}\left. \frac{\partial f_{t10-B}}{\partial N_{1Lt-B}} \right|_{x=x_{B}^{*}}+\lambda\left. \frac{\partial f_{t12-B}}{\partial R_{Lt-B}} \right|_{x=x_{B}^{*}}-\nabla_{N2-N2}\left. \frac{\partial f_{t12-B}}{\partial R_{Lt-B}} \right|_{x=x_{B}^{*}}-\left. \frac{\partial f_{t13-B}}{\partial N_{1Lt-B}} \right|_{x=x_{B}^{*}}\left. \frac{\partial f_{t10-B}}{\partial C_{PL-t-B}} \right|_{x=x_{B}^{*}};$$

$$d_{8}={-\nabla_{R-N1}\lambda-\nabla_{N2-R}\nabla_{R-N2}\lambda-\nabla_{N1-N2}\nabla_{N2-N1}\lambda+\nabla_{R-N1}\nabla_{N2-N2}+\nabla_{N2-R}\nabla_{N1-N2}\nabla_{R-N1}+\nabla_{N1-R}\nabla_{R-N2}\nabla_{N2-N1}-\nabla}_{N2-N2}\lambda\left. \frac{\partial f_{t10-B}}{\partial N_{1Lt-B}} \right|_{x=x_{B}^{*}}-\nabla_{N2-N2}\lambda\left. \frac{\partial f_{t12-B}}{\partial R_{Lt-B}} \right|_{x=x_{B}^{*}}+\nabla_{N2-R}\nabla_{R-N2}\left. \frac{\partial f_{t10-B}}{\partial N_{1Lt-B}} \right|_{x=x_{B}^{*}}+\nabla_{N1-N2}\nabla_{N2-N1}\left. \frac{\partial f_{t12-B}}{\partial R_{Lt-B}} \right|_{x=x_{B}^{*}}-\lambda\left. \frac{\partial f_{t10-B}}{\partial N_{1Lt-B}} \right|_{x=x_{B}^{*}}\left. \frac{\partial f_{t12-B}}{\partial R_{Lt-B}} \right|_{x=x_{B}^{*}}+\nabla_{N2-N2}\left. \frac{\partial f_{t10-B}}{\partial N_{1Lt-B}} \right|_{x=x_{B}^{*}}\left. \frac{\partial f_{t12-B}}{\partial R_{Lt-B}} \right|_{x=x_{B}^{*}}+\nabla_{N2-R}\lambda\left. \frac{\partial f_{t13-B}}{\partial N_{1Lt-B}} \right|_{x=x_{B}^{*}}\left. \frac{\partial f_{t12-B}}{\partial C_{PL-t-B}} \right|_{x=x_{B}^{*}}+\nabla_{N2-N1}\left. \frac{\partial f_{t13-B}}{\partial N_{1Lt-B}} \right|_{x=x_{B}^{*}}\left. \frac{\partial f_{t10-B}}{\partial C_{PL-t-B}} \right|_{x=x_{B}^{*}}+\nabla_{N1-R}\left. \frac{\partial f_{t13-B}}{\partial N_{1Lt-B}} \right|_{x=x_{B}^{*}}\left. \frac{\partial f_{t12-B}}{\partial C_{PL-t-B}} \right|_{x=x_{B}^{*}}+\nabla_{N2-N2}\left. \frac{\partial f_{t13-B}}{\partial N_{1Lt-B}} \right|_{x=x_{B}^{*}}\left. \frac{\partial f_{t10-B}}{\partial C_{PL-t-B}} \right|_{x=x_{B}^{*}}+\left. \frac{\partial f_{t10-B}}{\partial C_{PL-t-B}} \right|_{x=x_{B}^{*}}\left. \frac{\partial f_{t13-B}}{\partial z_{B}} \right|_{x=x_{B}^{*}}+\left. \frac{\partial f_{t13-B}}{\partial N_{1Lt-B}} \right|_{x=x_{B}^{*}}\left. \frac{\partial f_{t12-B}}{\partial R_{Lt-B}} \right|_{x=x_{B}^{*}}\left. \frac{\partial f_{t10-B}}{\partial C_{PL-t-B}} \right|_{x=x_{B}^{*}};$$

$$d_{9}=\nabla_{R-N1}\nabla_{N2-N2}\lambda{+\nabla_{N1-R}\nabla_{R-N2}\nabla_{N2-N1}\lambda+\nabla}_{N2-N2}\lambda\left. \frac{\partial f_{t10-B}}{\partial N_{1Lt-B}} \right|_{x=x_{B}^{*}}\left. \frac{\partial f_{t12-B}}{\partial R_{Lt-B}} \right|_{x=x_{B}^{*}}+\nabla_{N2-R}\nabla_{R-N2}\lambda\left. \frac{\partial f_{t10-B}}{\partial N_{1Lt-B}} \right|_{x=x_{B}^{*}}+\nabla_{N1-N2}\nabla_{N2-N1}\lambda\left. \frac{\partial f_{t12-B}}{\partial R_{Lt-B}} \right|_{x=x_{B}^{*}}-\nabla_{N2-R}\left. \frac{\partial f_{t12-B}}{\partial C_{PL-t-B}} \right|_{x=x_{B}^{*}}\left. \frac{\partial f_{t13-B}}{\partial z_{B}} \right|_{x=x_{B}^{*}}-\nabla_{N1-R}\nabla_{N2-N1}\left. \frac{\partial f_{t13-B}}{\partial N_{1Lt-B}} \right|_{x=x_{B}^{*}}\left. \frac{\partial f_{t12-B}}{\partial C_{PL-t-B}} \right|_{x=x_{B}^{*}}-\nabla_{N2-N1}\left. \frac{\partial f_{t10-B}}{\partial C_{PL-t-B}} \right|_{x=x_{B}^{*}}\left. \frac{\partial f_{t13-B}}{\partial z_{B}} \right|_{x=x_{B}^{*}}-\nabla_{N2-R}\nabla_{R-N1}\left. \frac{\partial f_{t13-B}}{\partial N_{1Lt-B}} \right|_{x=x_{B}^{*}}\left. \frac{\partial f_{t10-B}}{\partial C_{PL-t-B}} \right|_{x=x_{B}^{*}}-\nabla_{N2-R}\nabla_{N1-N2}\left. \frac{\partial f_{t13-B}}{\partial N_{1Lt-B}} \right|_{x=x_{B}^{*}}\left. \frac{\partial f_{t12-B}}{\partial C_{PL-t-B}} \right|_{x=x_{B}^{*}}-\nabla_{N1-R}\nabla_{N2-N2}\left. \frac{\partial f_{t13-B}}{\partial N_{1Lt-B}} \right|_{x=x_{B}^{*}}\left. \frac{\partial f_{t12-B}}{\partial C_{PL-t-B}} \right|_{x=x_{B}^{*}}-\nabla_{N2-R}\nabla_{R-N2}\left. \frac{\partial f_{t13-B}}{\partial N_{1Lt-B}} \right|_{x=x_{B}^{*}}\left. \frac{\partial f_{t10-B}}{\partial C_{PL-t-B}} \right|_{x=x_{B}^{*}}{-\nabla}_{N1-R}\left. \frac{\partial f_{t12-B}}{\partial C_{PL-t-B}} \right|_{x=x_{B}^{*}}\left. \frac{\partial f_{t13-B}}{\partial z_{B}} \right|_{x=x_{B}^{*}}-\nabla_{N2-N2}\left. \frac{\partial f_{t10-B}}{\partial C_{PL-t-B}} \right|_{x=x_{B}^{*}}\left. \frac{\partial f_{t13-B}}{\partial z_{B}} \right|_{x=x_{B}^{*}}{-\nabla}_{N2-R}\left. \frac{\partial f_{t10-B}}{\partial N_{1Lt-B}} \right|_{x=x_{B}^{*}}\left. \frac{\partial f_{t13-B}}{\partial N_{1Lt-B}} \right|_{x=x_{B}^{*}}\left. \frac{\partial f_{t12-B}}{\partial C_{PL-t-B}} \right|_{x=x_{B}^{*}}-\nabla_{N2-N2}\left. \frac{\partial f_{t13-B}}{\partial N_{1Lt-B}} \right|_{x=x_{B}^{*}}\left. \frac{\partial f_{t12-B}}{\partial R_{Lt-B}} \right|_{x=x_{B}^{*}}\left. \frac{\partial f_{t10-B}}{\partial C_{PL-t-B}} \right|_{x=x_{B}^{*}}+\nabla_{N2-N1}\left. \frac{\partial f_{t13-B}}{\partial N_{1Lt-B}} \right|_{x=x_{B}^{*}}\left. \frac{\partial f_{t12-B}}{\partial R_{Lt-B}} \right|_{x=x_{B}^{*}}\left. \frac{\partial f_{t10-B}}{\partial C_{PL-t-B}} \right|_{x=x_{B}^{*}}-\left. \left. \frac{\partial f_{t12-B}}{\partial R_{Lt-B}} \right|_{x=x_{B}^{*}}\frac{\partial f_{t10-B}}{\partial C_{PL-t-B}} \right|_{x=x_{B}^{*}}\left. \frac{\partial f_{t13-B}}{\partial z_{B}} \right|_{x=x_{B}^{*}};$$

$$d_{10}=\nabla_{N2-R}\nabla_{N1-N2}\nabla_{R-N1}\lambda+\nabla_{N1-R}\nabla_{N2-N1}\left. \frac{\partial f_{t12-B}}{\partial C_{PL-t-B}} \right|_{x=x_{B}^{*}}\left. \frac{\partial f_{t13-B}}{\partial z_{B}} \right|_{x=x_{B}^{*}}+\nabla_{N2-R}\nabla_{R-N1}\left. \frac{\partial f_{t10-B}}{\partial C_{PL-t-B}} \right|_{x=x_{B}^{*}}\left. \frac{\partial f_{t13-B}}{\partial z_{B}} \right|_{x=x_{B}^{*}}+\nabla_{N2-R}\nabla_{N1-N2}\left. \frac{\partial f_{t12-B}}{\partial C_{PL-t-B}} \right|_{x=x_{B}^{*}}\left. \frac{\partial f_{t13-B}}{\partial z_{B}} \right|_{x=x_{B}^{*}}+\nabla_{N1-R}\nabla_{N2-N2}\left. \frac{\partial f_{t12-B}}{\partial C_{PL-t-B}} \right|_{x=x_{B}^{*}}\left. \frac{\partial f_{t13-B}}{\partial z_{B}} \right|_{x=x_{B}^{*}}+\nabla_{N2-R}\nabla_{R-N2}\left. \frac{\partial f_{t10-B}}{\partial C_{PL-t-B}} \right|_{x=x_{B}^{*}}\left. \frac{\partial f_{t13-B}}{\partial z_{B}} \right|_{x=x_{B}^{*}}+\left. \frac{\partial f_{t12-B}}{\partial R_{Lt-B}} \right|_{x=x_{B}^{*}}\left. \frac{\partial f_{t10-B}}{\partial C_{PL-t-B}} \right|_{x=x_{B}^{*}}\left. \frac{\partial f_{t13-B}}{\partial z_{B}} \right|_{x=x_{B}^{*}}+\nabla_{N2-R}\left. \frac{\partial f_{t10-B}}{\partial N_{1Lt-B}} \right|_{x=x_{B}^{*}}\left. \frac{\partial f_{t12-B}}{\partial C_{PL-t-B}} \right|_{x=B}\left. \frac{\partial f_{t13-B}}{\partial z_{B}} \right|_{x=x_{B}^{*}}+\nabla_{N2-N2}\left. \frac{\partial f_{t12-B}}{\partial R_{Lt-B}} \right|_{x=x_{B}^{*}}\left. \frac{\partial f_{t10-B}}{\partial C_{PL-t-B}} \right|_{x=x_{B}^{*}}\left. \frac{\partial f_{t13-B}}{\partial z_{B}} \right|_{x=x_{B}^{*}}.$$

Using the Routh-Hurwitz criterion,

*Condition 8 :*$d_{6}>0.$

*Condition 9:* $d_{7}>0.$

*Condition 10:* $d_{8}>0.$

*Condition 11:* $d_{9}>0.$

*Condition 12:* $d_{10}>0.$

*Condition 13:* $d_{6}d_{7}d_{8}-d_{6}^{2}d_{9}-d_{8}^{2}>0.$

*Condition 14:* $\left( d_{6}d_{9}-d_{10} \right)\left( d_{6}d_{7}d_{8}-d_{8}^{2}-d_{6}^{2}d_{9} \right)-d_{10}\left( d_{6}d_{7}-d_{8} \right)^{2}-d_{6}d_{10}^{2}>0.$
